# Phylobetadiversity among Forest Types in the Brazilian Atlantic Forest Complex

**DOI:** 10.1371/journal.pone.0105043

**Published:** 2014-08-14

**Authors:** Leandro Da Silva Duarte, Rodrigo Scarton Bergamin, Vinícius Marcilio-Silva, Guilherme Dubal Dos Santos Seger, Márcia Cristina Mendes Marques

**Affiliations:** 1 Departamento de Ecologia, Universidade Federal do Rio Grande do Sul, Porto Alegre, Brazil; 2 Departamento de Botânica, Universidade Federal do Paraná, Curitiba, Brazil; Institute of Botany, Chinese Academy of Sciences, China

## Abstract

Phylobetadiversity is defined as the phylogenetic resemblance between communities or biomes. Analyzing phylobetadiversity patterns among different vegetation physiognomies within a single biome is crucial to understand the historical affinities between them. Based on the widely accepted idea that different forest physiognomies within the Southern Brazilian Atlantic Forest constitute different facies of a single biome, we hypothesize that more recent phylogenetic nodes should drive phylobetadiversity gradients between the different forest types within the Atlantic Forest, as the phylogenetic divergence among those forest types is biogeographically recent. We compiled information from 206 checklists describing the occurrence of shrub/tree species across three different forest physiognomies within the Southern Brazilian Atlantic Forest (Dense, Mixed and Seasonal forests). We analyzed intra-site phylogenetic structure (phylogenetic diversity, net relatedness index and nearest taxon index) and phylobetadiversity between plots located at different forest types, using five different methods differing in sensitivity to either basal or terminal nodes (phylogenetic fuzzy weighting, COMDIST, COMDISTNT, UniFrac and Rao’s H). Mixed forests showed higher phylogenetic diversity and overdispersion than the other forest types. Furthermore, all forest types differed from each other in relation phylobetadiversity patterns, particularly when phylobetadiversity methods more sensitive to terminal nodes were employed. Mixed forests tended to show higher phylogenetic differentiation to Dense and Seasonal forests than these latter from each other. The higher phylogenetic diversity and phylobetadiversity levels found in Mixed forests when compared to the others likely result from the biogeographical origin of several taxa occurring in these forests. On one hand, Mixed forests shelter several temperate taxa, like the conifers *Araucaria* and *Podocarpus*. On the other hand, tropical groups, like Myrtaceae, are also very representative of this forest type. We point out to the need of more attention to Mixed forests as a conservation target within the Brazilian Atlantic Forest given their high phylogenetic uniqueness.

## Introduction

Phylobetadiversity can be defined as the phylogenetic resemblance between communities or biomes [1]. The distribution of species belonging to different phylogenetic clades across the biogeographic space is often explained by major climatic conditions [2], [3]. On the other hand, the geographical distribution of different taxa also depends on historical processes promoting speciation and dispersal [4], [5]. Indeed, in some cases past ecological conditions can explain the diversity patterns of different lineages better than current environmental gradients [6]. Thus, environmental, evolutionary, historical and neutral factors likely interact to determine species composition patterns [5]. In this context, any biome might be thought as a snapshot of multiple interactions among those factors molding the distribution of taxa.

The resemblance between different phylogenetic lineages along a phylogenetic tree expresses a temporal accumulation of evolutionary divergence among clades. Evolutionary divergence near the root node of the phylogenetic tree reflects events occurred in remote past, while divergence near the terminal tips indicates recent evolutionary events. Further, we are likely to find contrasting phylobetadiversity patterns, depending on the analytical approach used to assess them [3]. Therefore, using different phylobetadiversity measures might help us to investigate whether the phylogenetic divergence between an array of sites has occurred more recently or deeper in the past. Nothing else being different, two sites located in the same biome are expected to vary more in relation to the occurrence of more recent nodes (e.g. families, genera), than in relation to more basal nodes (*e.g.* superorders, classes). On the other hand, sites located in different biomes might be expected to differ more in relation to more basal phylogenetic nodes than local sites within the same biome, as the respective biomes diverged earlier in terms of historical development than local sites within the same biome.

The Atlantic Forest is one of the most widely distributed tropical forests in Southern America, occupying almost all Brazilian Eastern coast besides inland areas. It is considered a hotspot for biodiversity conservation due to its high endemism and threatened areas [7], [8]. It shelters about 15,000 vascular plants, from which 48% of species are endemic [9]. Actually, endemism levels in Atlantic Forest are among the highest observed in the world [10], [11]. The Atlantic Forest biota is composed by taxa from different biogeographic origins, notoriously from the Amazonian Forest, the gallery forests of Cerrado, and the Andean areas in the austral portion of the biome [12], [13]. Based on species distribution, the vegetation of the Atlantic Forest is recognized as composed by three forest types resulting from the differential influence of bordering floras: dense, mixed and seasonal forests [14]–[16]. In Material and Methods we provide a more detailed description of these different forest types. Floristic variation within and among different forest types within the Brazilian Atlantic Forest is strongly determined by environmental gradients [15], [17], [18]. On the other hand, it is widely recognized the biogeographically common origin of the different vegetation types within the Atlantic Forest [15], [19]. Climate in South-America had been wetter and hotter by the beginning of the Eocene, and the Atlantic and the Amazonian Forest formed a unique large forest from Pacific to Atlantic oceans [20], [21]. However, from the Pliocene, with the global climatic cooling and drying, an expansion of open vegetation types of Cerrado (Brazilian savanna), Caatinga and Chaco had occurred, which have disrupted the connection between the Atlantic Forest from other South-American forests. Since then, the Atlantic Forest is likely to have evolved as a single biogeographic unit [20].

To our knowledge, no attempts of analyzing a possible phylogenetic differentiation among these floras have yet been done. In this study we aim at carrying out such analysis, focusing mainly on phylobetadiversity patterns. Analyzing phylogenetic gradients among different forest physiognomies within the Atlantic Forest is crucial to understand the historical affinities between them. Based on the widely accepted idea that different forest physiognomies within the Atlantic Forest constitute different facies of a single eco-evolutionary entity, we hypothesize that recent nodes should drive phylobetadiversity gradients between the different forest types within the Southern Brazilian Atlantic Forest, as the phylogenetic divergence among them is biogeographically recent. To test this hypothesis, we compiled information from 206 floristic checklists describing the occurrence of shrub/tree species across the Southern Brazilian Atlantic Forest. Based on that da we evaluated the phylogenetic structure of different Atlantic Forest types and compared those forest types in relation to phylobetadiversity using five distinct analytical methods, which captured phylobetadiversity patterns more related to either basal or recent phylogenetic nodes. A second goal of this study is methodological. Although we have previously employed phylogenetic fuzzy weighting [22] to evaluate phylogenetic gradients across sets of communities or ecoregions [18], [23], [24], we have never compared the patterns we found with those generated by other methods for phylobetadiversity analysis. Given the first goal of the study, we think we have an excellent opportunity of providing such comparison, which can improve the general understanding on the method.

## Materials and Methods

### The Southern Brazilian Atlantic Forest

The Atlantic Forest extends along the Brazilian coast and inwards to eastern Paraguay and Northeastern Argentina, across variable climatic conditions with elevations ranging from sea level to 2,900 m [14]. This includes, approximately, latitudes ranging from 5° N to 33° S, longitudes from 35° W to 52° W and altitudes from 0 to 2,200 m [14]. Such broad geographical variation determines a climatic gradient related to annual rainfall (approximately from 800 to 4,000 mm) and mean annual temperatures (averages from 15° to 25°C), which influence species distributions [25]–[27]. In the south and southeast Brazil the Atlantic Forest is marked by the occurrence of three forest types [15], the Dense Rain Forest (hereafter Dense forests), the Mixed Rain Forest (hereafter Mixed forests) and the Seasonal Deciduous and Semideciduous Forest (hereafter Seasonal forests).

### The Dense forests

Dense forests are associated with the Atlantic coast and include a large area of lowland (until ∼50 m a.s.l.) and slope (∼50 to 2,200 m a.s.l.) forests from the Northeastern to the Southern regions of Brazil. The climate is variable, but generally hot and wet in lowlands and cold and wetter in slopes [14], [15]. This biome shows floristic affinities with the Amazon Forest and Caatinga in the North [26], [28], [29] and it is influenced by the flora of other regions, such as the Andes and elements of the ancient southern Gondwana in the South [30]. The vegetation in lowlands comprises forests and scrubs that occur in drier climates (restingas) and in wetter climates (rain forests), determined by rainfall and soil sandiness [27]. Among species that determine vegetation in the coastal plain are *Maytenus obtusifolia*, *Byrsonima sericea*, *Ilex theazans, Calophyllum brasiliense*, *Ocotea pulchella* and *Myrcia multiflora* [27]. In the slopes, forests are highly differentiated by altitude, and species such as *Drimys brasiliensis, Ilex microdonta, Weinmannia paulliniifolia* characterize the vegetation [31].

### Mixed forests

Mixed forests, also known as *Araucaria* forests, constitute the main forest type on the highland plateau in southern Brazil at elevations above 500 m a.s.l. [32]. Its northern distribution limit is in the Serra da Mantiqueira in south-eastern Brazil (latitude 20°S), where it occurs as vegetation patches or as isolated individuals in high-altitude grasslands, above 1,000 m. Southwards, mixed forests extend to latitude 29° S [32]. These forests are subjected to tropical and sub-tropical humid climates without pronounced dry periods. The annual rainfall ranges from 1400 to 2200 mm, and the annual mean temperature ranges mainly from 12°C to 18°C [33]. The presence of species phytogeographically related to temperate Austral-Antarctic and Andean floras distinguishes communities within the Mixed Forest from more tropical facies of Brazilian Atlantic forests [34]. Besides *Araucaria angustifolia*, some other typical species found in those forests are *Podocarpus lamberti* (conifer), *Dicksonia sellowiana* (tree fern), *Drimys* spp. (Winteraceae), and several species of Myrtaceae, Melastomataceae and Lauraceae.

### Seasonal forests

Seasonal forests are related to the hinterland Parana River basin in the south and southeast Brazil. These forests are characterized by two distinct seasons with marked alternation from tropical with intense summer rainfalls to subtropical with low winter temperatures and scarce precipitation. During the cold and dry period, 20% to 50% of the canopy trees fall their leaves (deciduous) [14]. The mean temperature in the winter is lower than 15°C. The flora of Seasonal forests is often influenced by taxa typical from Cerrado (Brazilian savannah) and the alternation between wet/hot summers and dry/cold winters influences the leaf longevity causing leaf fall on winter [15]. This forest type has a dominance of species of *Parapiptadenia, Peltophrum, Cariniana, Lecythis, Tabebuia, Astronium* among others [14].

### Species occurrences in floristic plots

We compiled information from 206 floristic checklists (Appendix S1) describing the occurrence of shrub/tree species across the geographic range of the Southern Brazilian Atlantic Forest biome (63 Dense forests, 50 Mixed forests, and 96 Seasonal forests). Floristic data were obtained by employing several distinct methodologies (Appendix S1). For instance, some authors used quadrats while others had no pre-defined surveying area; some used diameter at breast height as inclusion criteria while others used plant height. For this reason we only considered species presence/absence in sites. We checked for recent synonyms in the Missouri Botanical Garden (http://www.tropicos.org), The Plant List (http://www.theplantlist.org/), and Flora do Brasil databases (http://floradobrasil.jbrj.gov.br). Undetermined species, which represented in average less than 4% of the number of species in each checklist, were not included in the floristic dataset. Clade names followed Smith et al. [35] and Chase & Reveal [36]. Thus, the complete floristic data set was arranged in sites-by-species matrix of 206 sites described by 1,916 species, which was used for the analyses.

We compared the forest types in relation to the logarithmic number of species recorded in each plot by using one-way ANOVA. *P*-values were obtained by a permutation test with 999 iterations [37]. *P-*values were calculated based on the number of times the observed F-value was lower than the random F-values computed at each permutation procedure. We also compared forest types in relation to the occurrence of species in the plots. For this, we performed a PERMANOVA with permutation test (999 iterations), using Jaccard index as resemblance measure [37], [38]. For both analyses, whenever a significant *P*-value was obtained, we performed pairwise contrast analysis to test which group differed from others. The significance of contrasts was also evaluated by permutation, in a similar way as in ANOVA and PERMANOVA [37]. Analyses were performed in the R environment (available at http://www.r-project.org), using package *vegan* 2.0–10 ([39], available at http://cran.r-project.org/web/packages/vegan/).

### Building a phylogenetic tree for Atlantic Forest plants

To define phylogenetic affinities among plant species we used the phylogenetic hypothesis of APG III [40] for angiosperms and the hypothesis of Burleigh *et al*. [41] for gymnosperms, which solve phylogenetic relationships to the family level. For this, we used the megatree R20120829 (available at https://github.com/camwebb/tree-of-trees/blob/master/megatrees/R20120829.new), removed outdated intrafamilial resolution and included the gymnosperms tree into the megatree. Since phylogenetic uncertainties influence different phylogenetic metrics, to reach intrafamilial node resolution we also included 51 constructed angiosperms families’ trees based on recent studies (families with more than one species and for which reliable phylogenetic hypotheses are available) (references in Appendix S2). This procedure solved genera relationships for 84% of the species in the database. We defined branch lengths using node age estimates proposed by Bell *et al.* [42] and the age estimates of Magallón et al. [43] for clades older than angiosperms, using only clade age estimates that were consistent with the APG III tree topology. We also included clade age estimates within some of the 51 families added to the megatree (references in Appendix S2). Undated nodes were adjusted using the BLADJ algorithm of Phylocom 4.2 software [44] and the phylogenetic tree was obtained using the Phylomatic 2 module of Phylocom 4.2 software [45]. Then we computed the phylogenetic pairwise patristic distances between species.

### Analyzing phylogenetic structure within Atlantic Forest types

We analyzed the phylogenetic structure of forest plots using different methods, in order to capture distinct properties of the phylogenetic structure of the plots. Since our species-by-sites matrix had only occurrences, no methods employed took into account species abundances. Phylogenetic diversity (PD) was computed as the total sum of branch lengths for species occurring in each plot [46]. Phylogenetic clustering/overdispersion was measured using the two metrics proposed by Webb et al. [47]: mean phylogenetic distances (MPD) between the species present in each plot, and mean phylogenetic distance between each species and its phylogenetically nearest species (MNTD). For PD, MPD and MNTD values we computed standardized effect sizes (SES) based on 999 null values obtained from a null model that keeps the species composition of the plot while the position of each species in the phylogenetic tree for the regional species pool (defined by all species present in the dataset) is freely shuffled (“taxa.label” model), as follows:
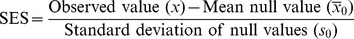



Hereafter, we refer only to the standardized values of theses methods, respectively SES.PD, NRI (net relatedness index) and NTI (nearest taxon index). Positive or negative SES.PD values indicate, respectively, phylogenetic diversity higher or lower than expected by the null model. Positive or negative NRI/NTI values indicate, respectively, phylogenetic clustering or overdispersion of species in the plot. While NRI captures the influence of deeper phylogenetic nodes to the phylogenetic structure of the plot, NTI characterizes the effect of shallower phylogenetic nodes [47]. Phylogenetic structure measures were computed in the R environment (available at http://www.r-project.org), using the package *picante* 1.6–2 ([48], available at http://cran.at.r-project.org/web/packages/picante/).

We compared the forest types in relation to phylogenetic structure methods (SES.PD, NRI and NTI) by using one-way ANOVA. *P*-values were obtained by a permutation test with 999 iterations [37]. For both analyses, whenever a significant *P*-value was obtained, we performed pairwise contrast analysis to test which group differed from others [37]. The significance of contrasts was also evaluated by permutation, in a similar way as in ANOVA [37]. Analyses were performed in the R environment (available at http://www.r-project.org), using package *vegan* 2.0–10 ([39], available at http://cran.r-project.org/web/packages/vegan/).

### Analyzing phylobetadiversity among Atlantic Forest types

We compared the different forest types in relation to phylobetadiversity patterns using five methods: phylogenetic fuzzy weighting [22], COMDIST [44], COMDISTNT [44], UniFrac [49] and Rao’s H [50]. As our species-by-sites matrix contained only species occurrences, all phylobetadiversity metrics were defined to do not consider species abundances. As some methods are more sensitive to variation in deeper phylogenetic nodes (COMDIST) while others capture variation mostly associated with shallower nodes (COMDISTNT, UniFrac and Rao’s H), using several indices to analyze phylobetadiversity patterns might help us to understand to what extent phylobetadiversity levels are explained by more basal or recent nodes [3]. On the other hand, phylogenetic fuzzy weighting is likely to capture phylobetadiversity patterns associated with both basal and more terminal nodes [18]. Therefore, using these five different methods enabled us to test our hypothesis on the phylogenetic relationships of different forest types within the Southern Brazilian Atlantic Forest.

Phylogenetic fuzzy weighting is a method developed to analyze phylobetadiversity patterns across metacommunities, based on fuzzy set theory [22]. The method is based on the computation of matrix **P** from the species-by-sites incidence matrix [22], [24]. The procedure consists of using pairwise phylogenetic similarities between species to weight their occurrence in the plots. The first step involves transforming pairwise phylogenetic distances into similarities ranging from 0 to 1. For this, each distance value *d_ij_* is converted into a similarity *s_ij_* using.
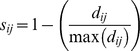
where max (*d_ij_*) is the maximum observed distance between two species in the tree.

Each phylogenetic similarity between a pair of species (*s_ij_*) is then divided by the sum of similarities between the species *i* and all other *k* species. This procedure generates phylogenetic weights for each species in relation to all others, expressed as.
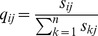



Such phylogenetic weights (*q_ij_*) expresses the degree of phylogenetic belonging of each taxon *i* in relation to all others [22]. The degree of phylogenetic belonging reflects the amount of evolutionary history shared between a given species and all others in the dataset. The second analytical step consists of incorporating those standardized phylogenetic weights into the species-by-sites matrix. The occurrence of each species *i* in a plot *k* (*w_ik_*) is distributed among all other *j* species occurring in that plot, proportionally to the degree of phylogenetic belonging between each pair of species as follows:
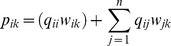



This procedure generates a matrix describing phylogeny-weighted species composition for each plot (matrix **P**), which expresses the representativeness of different lineages across the sites (see Duarte *et al.* [24] for a detailed description). Phylogenetic fuzzy weighting was performed in the R environment (available at http://www.r-project.org), using the package *SYNCSA* 1.3.2 ([51], available at http://cran.r-project.org/web/packages/SYNCSA/). Pairwise phylobetadiversity between plots was obtained by computing squared-rooted Bray-Curtis dissimilarities (or other appropriate resemblance measure, see Legendre & Anderson [52]) for every pair of plots in matrix **P** (Table 1).

**Table 1:** Phylobetadiversity methods used to compare different forest types within the Southern Brazilian Atlantic Forest.

Method	Formula	Description	Reference
Phylogeneticfuzzy weighting	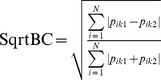	Computes the square-rooted Bray-Curtisdissimilarity between plots *k*_1_ and *k*_2_ based onphylogenetically weighted incidence (*p_ik_*) of*N* species *i*.	[22]
COMDIST	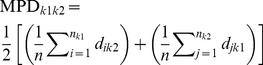	Computes the mean pairwise phylogenetic distance*d* between each species *i* of plot *k*_1_ and all *n* speciesof plot *k*_2_.	[44]
COMDISTNT	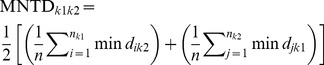	Computes the mean pairwise phylogenetic distance*d* between each species *i* of plot *k*_1_ and thephylogenetically nearest species of plot *k*_2_(min *d*_ik2_).	[44]
Rao’s H	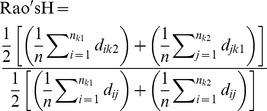	Standardized measure of phylogenetic distinctness.The numerator is similar to COMDIST. Thedenominator is the mean phylogenetics distancewithin-plots.	[50]
UniFrac	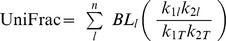	Computes the fraction of total branch lengthlinking the species occurring in two plots, which isexclusive to each plot.	[49]

We adopted this method to analyze phylobetadiversity because it allows to decompose phylogenetic gradients across an array of plots into orthogonal eigenvectors and, more importantly, to evaluate which clades are related to each phylogenetic eigenvector [24]. We achieved this by performing a PCoA [53] based on the square-rooted Bray-Curtis dissimilarities between pairs of plots previously computed on matrix **P**. Such procedure generated principal coordinates of phylogenetic structure (PCPS) for each floristic plot. Each PCPS is a vector describing an orthogonal phylogenetic gradient in the dataset [18], [23]. The PCPS with the highest eigenvalue describes broader phylogenetic gradients related to the split of the deepest tree nodes across the dataset, such as that connecting conifers and angiosperms. As the eigenvalues of the other PCPS decrease, finer phylogenetic gradients related to splits of shallower nodes (*e.g.* families, genera) are described [18]. By relating the correlation between species from major clades and the PCPS eigenvectors, we can draw a scatterplot relating directly sites and species grouped in clades. PCPS analysis was performed using the package *PCPS* (available at http://cran.r-project.org/web/packages/PCPS/) of the R environment (available at http://www.r-project.org). Further, we compared the forest types in relation to the PCPS eigenvectors containing more than 5% of total variation in matrix **P** using one-way ANOVA. *P*-values were obtained by a permutation test with 999 iterations [37]. Such analysis allowed us to define which phylogenetic gradients were mostly related to different Atlantic forest types. ANOVA was performed in the R environment (available at http://www.r-project.org), using package *vegan* 2.0–10 ([39], available at http://cran.r-project.org/web/packages/vegan/).

Furthermore, we employed other four well-known phylobetadiversity measures to compare the forest types within the Southern Brazilian Atlantic Forest (see Table 1). COMDIST is a phylobetadiversity measure that computes the mean phylogenetic distance among species occurring in two different sites [44]. For this reason, this phylobetadiversity measure captures variation associated with the more basal nodes linking species [3]. Computing COMDIST values without considering the variation in species abundances is equivalent to compute the phylogenetic distinctness (Rao’s D) proposed by Hardy & Senterre [50]. Thus, we opted for using only the former in this study. On the other hand, by standardizing Rao’s D values by the mean within-site phylogenetic diversity it is possible to obtain another phylobetadiversity measure (Rao’s H, [50]), which captures phylobetadiversity patterns related to more terminal nodes in the tree [3]. COMDISTNT [44] measures the mean phylogenetic distance between every species in a plot and the nearest phylogenetic neighbor in another site (Table 1). It is, therefore, a “terminal node” metric [3]. The last phylobetadiversity method used in this study was UniFrac [49], which measures, for each pair of sites, the fraction of the total branch length of phylogenetic tree that is exclusive to each site (Table 1). Since more basal nodes are likely to be shared by most species, UniFrac captures phylobetadiversity patterns related to more terminal nodes [3]. This method is mathematically equivalent to the Jaccard index when a star phylogeny is considered [49]. UniFrac gives very similar (but not exactly similar) results when compared to PhyloSor [3], which is another well-known phylobetadiversity measure [54]. For this reason, we opted for using only the former. COMDIST, COMDISTNT and Rao’s H were computed in the R environment (available at http://www.r-project.org), using the package *picante* 1.6–2 ([48], available at http://cran.at.r-project.org/web/packages/picante/). UniFrac was computed using the R package *GUniFrac* 1.0 (available at http://cran.r-project.org/web/packages/GUniFrac/index.html).

We carried out Mantel tests [53] based on Pearson correlations (999 permutations) to evaluate the association between pairwise phylobetadiversity values obtained from matrix **P** and all the other methods (COMDIST, COMDISTNT, UniFrac and Rao’s H). Furthermore, we performed PERMANOVA with permutation test (999 iterations) [37], [38] using each pairwise phylobetadiversity method as resemblance measures, to compare different forest types in relation to phylobetadiversity levels. Whenever a significant *P*-value was obtained for the general model, we performed pairwise contrast analysis to test which group differed from the others [34]. The significance of contrasts was also evaluated by permutation, in a similar way as in PERMANOVA [34]. Analyses were performed in the R environment (available at http://www.r-project.org), using package *vegan* 2.0–10 ([39], available at http://cran.r-project.org/web/packages/vegan/).

## Results

From the 1,916 species occurring across the Southern Brazilian Atlantic Forest, eurosids (superorder Rosanae) comprised 58% of total number of species, asterids (superorder Asteranae) were represented by 25% of species in the dataset, and magnoliids (superorder Magnolinae) by 10%. Other phylogenetic clades occurring in the dataset were Caryophyllales and monocots (superorder Lilianae) (each comprising 2% of total richness), and, Proteanae, Santalanae, conifers (superorder Pinidae), Dillenianae, Chloranthanae and Ranunculanae, each with ≤1% of total number of species. The 10 more frequent species in the dataset were, in decreasing order, *Casearia sylvestris* (Salicaceae), *Myrsine umbellata* (Myrsinaceae), *Cupania vernalis* (Sapindaceae), *Allophylus edulis* (Sapindaceae), *Matayba elaeagnoides* (Sapindaceae), *Casearia decandra* (Salicaceae), *Zanthoxylum rhoifolium* (Rutaceae), *Campomanesia xanthocarpa* (Myrtaceae), *Guapira opposita* (Nyctaginaceae) and *Prunus myrtifolia* (Rosaceae).

**Figure 1:**
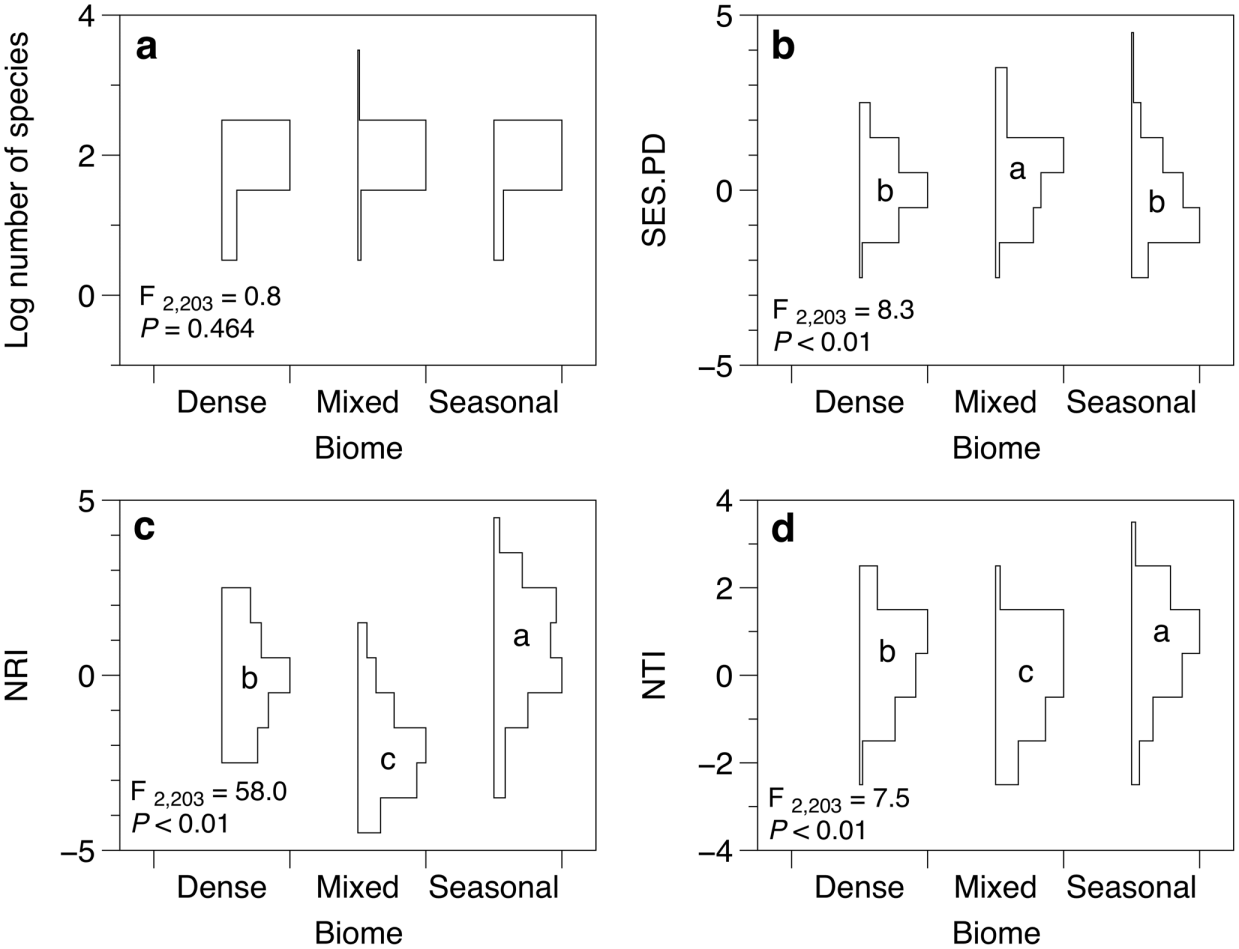
ANOVA with permutation tests for a) logarithmized species number, b) standardized phylogenetic diversity (SES.PD), c) net relatedness index (NRI) and d) nearest taxon index (NTI) for floristic plots occurring in different forest types within the Southern Brazilian Atlantic Forest. Probability plots drawn for each forest type define the relative frequency of values for each response variable. *P*-values obtained using 999 permutations. Different letters within the probability plots indicate significant difference between forest types (*P*≤0.05).

We found 946 species in Mixed forests, 1,136 in Dense forests and 1,187 in Seasonal forests. ANOVA results showed that different forest types did not show significant variation in relation the number of species (Fig. 1a). This finding gives support to the significant variation found in relation to the three phylogenetic structure metrics analyzed. Mixed forests showed higher standardized phylogenetic diversity (Fig. 1b) and lower NRI values, indicating phylogenetic overdispersion, than the other forest types (Fig. 1c). By its turn, Seasonal forests showed lower standardized phylogenetic diversity and higher NRI values, indicating phylogenetic clustering. Dense forests presented intermediary values between Mixed and Seasonal forests. In relation to NTI, Seasonal forests showed higher values than the other two forest types, indicating phylogenetic clustering (Fig. 1d), while Mixed and Dense forests did not vary in relation to each other.

Mantel tests showed that dissimilarities computed based on matrix **P** had significant Mantel correlations with all other phylobetadiversity methods. The highest correlation was between phylogenetic fuzzy weighting and COMDIST (ρ = 0.59; *P* = 0.001), followed by Rao’s H (ρ = 0.48; *P* = 0.001), COMDISTNT (ρ = 0.48; *P* = 0.001) and UniFrac (ρ = 0.39; *P* = 0.001).

MANOVA indicated that species composition of floristic plots varied significantly (*P*<0.001) between all forest types (Table 2). Nonetheless, the model fit for species composition was worse than for almost all phylobetadiversity methods (exception for COMDIST, see Table 2), indicating that phylobetadiversity patterns observed in this study were robust, and not merely an artifact of the variation in species composition between forest types. Among the phylobetadiversity methods, phylogenetic fuzzy weighting showed the best model fit (R^2^ = 0.42; F = 73.4). Although PERMANOVA showed significant results for the other four methods, their model fit varied according to the properties of the method. COMDIST, a phylobetadiversity method that captures patterns related to more basal nodes, showed a very poor (although statistically significant) fit, while the other three metrics, which capture phylobetadiversity patterns related to terminal nodes showed better fit, especially Rao’ H. Taking into account only the two methods with best model fit (phylogenetic fuzzy weighting and Rao’s H), we found that most phylobetadiversity variation (higher F-value) was observed between Mixed and Seasonal forests. On the other hand, while phylogenetic fuzzy weighting showed a higher phylogenetic similarity between Dense and Seasonal forests (lower F-value), Rao’s H showed a higher similarity between Mixed and Dense (Table 2).

**Table 2:** PERMANOVA with permutation tests comparing species composition and five different phylobetadiversity methods between different forest types within the Southern Brazilian Atlantic Forest.

Response variable	Overall PERMANOVA model		F-values for pairwise contrasts		
	R^2^	F_2,203_	Mixed - Dense	Mixed - Seasonal	Dense - Seasonal
Species composition	0.081	9.0	9.5	8.3	9.3
Phylogenetic fuzzy weighting	0.420	73.4	65.8	128.8	16.2
COMDIST	0.019	2.0	1.4	2.2	2.1
COMDISTNT	0.230	30.3	32.5	37.9	23.1
Rao’s H	0.340	52.2	18.2	68.6	58.1
UniFrac	0.135	15.9	19.1	18.6	11.6

All F-values showed *P*-values <0.001. *P*-values obtained by 999 permutations.

**Figure 2:**
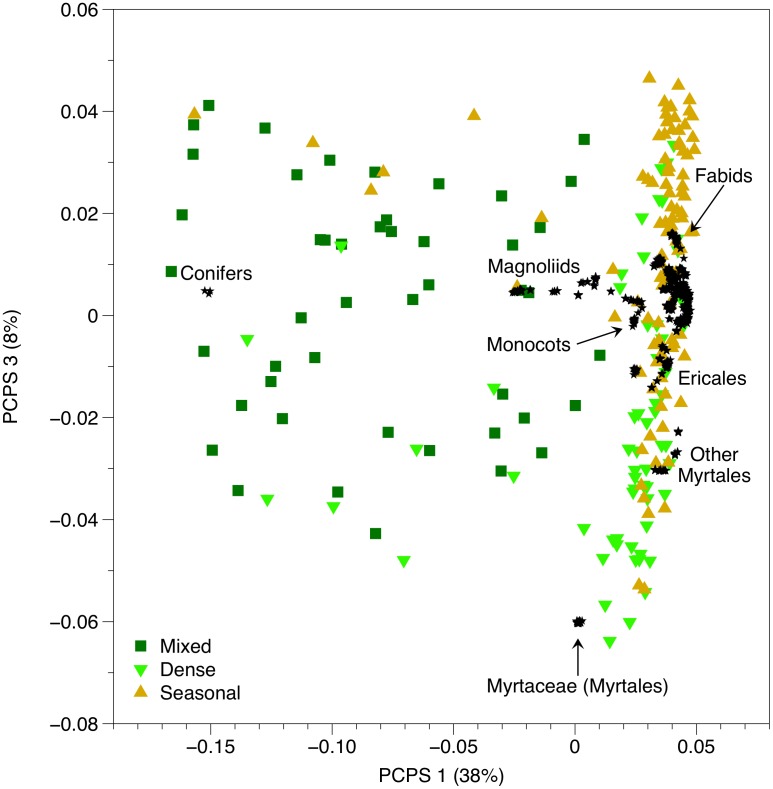
Scatter plots of the PCPS 1 and 3 generated from the ordination of matrix P describing phylogenetic weighted species composition of floristic plots located in different forest types (Mixed, Dense and Seasonal) within the Southern Brazilian Atlantic Forest.

The ordination of matrix **P** enabled us to explore the phylogenetic clades underlying phylobetadiversity patterns (Fig. 2). The four first PCPS axes contained more than 5% of total information in matrix **P** (explained together 59% of the total variation in matrix **P**). These four PCPS were then submitted to ANOVA. The test comparing the scores of PCPS 1 between forest types showed the best fit (F_2,203_ = 129.5; *P*<0.001), followed by PCPS 3 (F_2,203_ = 35.5; *P*<0.001). The first PCPS (38% of total variation in matrix **P**) captured phylobetadiversity patterns related to the most basal node, *i.e.* the node separating conifers from angiosperms drove the variation between forest types, with Mixed forests (related to conifers) splitting from Dense and Seasonal forests (related to angiosperms). The phylogenetic gradient along the third PCPS axis (8% of total variation in matrix **P**) was mostly driven by rosids (Fig. 2). While Dense forests were positively related to the occurrence of Myrtaceae and other Myrtales groups, Seasonal forests were positively associated with the occurrence of fabid rosids. PCPS 2 and 4 contained 9% and 5% of total variation in matrix **P**, respectively. ANOVA for these two PCPS showed poorer fit when compared to the former ones (F_2,203_ = 8.1 and F_2,203_ = 22.6, respectively).

## Discussion

The classification of the Brazilian Atlantic Forest into different forest types was demonstrated here to have a phylogenetic basis. Except for COMDIST, all other phylobetadiversity metrics captured the variation between forest types within the Brazilian Atlantic Forest better than species composition alone (see also [24]). Actually, the most frequent species in the dataset are widely distributed across the Atlantic Forest, occurring in different forest types and under variable habitat conditions. Those species show high ecological plasticity, as they are capable to live under contrasting environmental conditions and soil types, are all dispersed by the fauna and show high tolerance to sunny environments. It is also noteworthy that none of these species are endemic from Brazil [55]–[57]. Considering the common biogeographic origin of different Atlantic Forest types [15], [19], we hypothesized that more terminal phylogenetic nodes should drive phylobetadiversity patterns between different forest types within the Southern Brazilian Atlantic Forest. Indeed, the phylobetadiversity methods sensitive to phylogenetic gradients related to more terminal nodes (COMDISTNT, UniFrac and Rao’s H, see [3]) captured phylobetadiversity variation between the forest types better than the “basal metric” (COMDIST). On the other hand, phylogenetically fuzzy weighting, which is likely to capture both the variation at basal and terminal nodes [18], showed the best model fit when we compared the different forest types. In general, all methods showed that Mixed forests differed more in relation to Dense and Seasonal forests than these latter from each other. The first PCPS captured phylogenetic gradient splitting conifers from other angiosperms (a basal node-driven gradient), which separated Mixed forests (related to conifers) from the other forest types (related to angiosperms), while the third PCPS captured a phylogenetic gradient related to more intermediary nodes (Myrtales related to Dense forests, fabids related to Seasonal forests). In general, the results from phylobetadiversity analysis showed that Mixed forests present a distinctive phylogenetic signature when compared to other Atlantic forests. To some extent, such patterns might be generated by the higher intra-site phylogenetic diversity found in Mixed forests when compared to other forest types. Nonetheless, the second phylobetadiversity method with higher fit in the comparison between forest types was Rao’s H, which standardize phylobetadiversity by the mean intra-site phylogenetic diversity [3], [50], reinforcing the patterns found here.

Mixed forests not only differed more in relation to phylobetadiversity from Dense and Seasonal forests than these latter from each other, but also showed higher intra-site phylogenetic diversity/overdispersion. This finding might be explained, on the one hand, by the importance of temperate conifers (*Araucaria*, *Podocarpus*) and magnoliids (e.g. *Drimys*, *Cinnamodendron*) for the flora of Mixed forests [34]. On the other hand, tropical Myrtaceae also constitutes an important eudicot group in Mixed forests, especially in those areas more directly connected with Dense forests [58]. Myrtaceae is the fourth largest plant family in Brazil [59], being the richest family in terms of tree species in several vegetation types, specially in Dense and Mixed Atlantic forests [60]. The floristic mixture found in Mixed forests is possibly influenced by the phylogenetic niche conservatism of the species occurring at more tropical sites of the Atlantic Forest, which precludes the advance of tropical species over the subtropical sites, allowing the permanence of several temperate taxa in Mixed forests [61]. As a consequence, Mixed forests is likely to show higher phylogenetic diversity and also higher degree of phylobetadiversity in relation to other Atlantic Forest types. The South American biota is formed by a northern tropical component and a southern temperate component, each with different biogeographic affinities [62]–[64]. The northern and southern portions of South America have always been connected, except during a brief period during the Cretaceous (100–80 Mya) when an epicontinental sea separated both halves of the American continent. The temperate taxa present in the Mixed forests had origin in the Southern Temperate Gondwana Province, namely Australia, New Zealand and New Caledonia [30]. The land connections between South America and West Antarctic continent allowed floristic exchanges between Australia and South America until the late Eocene (∼35 million years) or even the early Oligocene (30–28 million years). Such floristic connections provided a stock of subtropical taxa in South America [13]. On the other hand, the tropical taxa widely distributed across the Brazilian Atlantic Forest derived from Northern hemisphere ancestors through Laurasian migrations [13], [65]. Thus, Mixed forests represent nowadays a unique mix of floras with distinct biogeographic and phylogenetic origins. The biogeographic features of Mixed forests increase the need of more effective conservation efforts to preserve that forest type, which has been suffered over the last century intensive human-made degradation due to logging, cattle-grazing and, more recently, silviculture [66]. Mixed forests need more attention from conservationists and decision-makers, as they have been often neglected as a conservation hotspot [67].

Our second goal in this study was to evaluate to what extent phylogenetic fuzzy weighting provides comparable values in relation to other phylobetadiversity methods. Swenson [3] showed that the association between different phylobetadiversity methods was due to the sensitivity of each method to more basal or terminal nodes of the phylogenetic tree. In this study, the highest correlation was observed between phylogenetic fuzzy weighting and COMDIST, which is a “basal” method [3]. Nonetheless, phylogenetic fuzzy weighting was also well correlated with the “terminal” methods (COMDISTNT, UniFrac and Rao’s H), which reinforces the fact that phylogenetic fuzzy weighting captures phylobetadiversity from both basal and terminal nodes [18]. Such property of the method can be verified by means of the computation of principal coordinates of phylogenetic structure (PCPS), which provides independent phylogenetic gradients between a set of sites where each gradient captures node splits from basal to more terminal nodes [18], [23], [24]. Moreover, the possibility of exploring the identity of the different clades driving phylobetadiversity among the sites represents an advantage offered by phylogenetic fuzzy weighting in relation to other widely employed methods. Given the diversity of methods for assessing phylobetadiversity patterns, we think that employing different approaches simultaneously improves our capability to explore phylogenetic gradients across a set of communities, ecoregions or biomes.

## Supporting Information

Appendix S1 **Characteristics of Southern Brazilian Atlantic Forest sites that were used in the analysis.**
(DOC)

Appendix S2 **Time-calibrated phylogenetic tree used to carry out phylogenetic structure and phylobetadiversity analyses.**
(DOCX)
