# Supplementary material for: Phylobetadiversity among Forest Types in the Brazilian Atlantic Forest Complex
Source: PLoS One. 2014 Aug 14;9(8):e105043. doi: 10.1371/journal.pone.0105043 (PMC4133375; doi:10.1371/journal.pone.0105043)
Supplement: Appendix S1 — Characteristics of Southern Brazilian Atlantic Forest sites that were used in the analysis. (DOC) [file pone.0105043.s001.doc]

**Appendix S1**: Characteristics of the Southern Brazilian Atlantic Forest sites that were used in the analysis. The complete database is available under request at http://www.ufrgs.br/leff/resources_english.html

| Vegetation type | Site | State | Coordinates | | Inclusion criteria | Method | Effort | Source |
| --- | --- | --- | --- | --- | --- | --- | --- | --- |
|  |  |  | Latitude | Longitude |  |  |  |  |
| Mixed Forest | Encruzilhada do Sul | RS | 30º 30' S | 52º 42' W | †DBH ≥ 5 cm | Plot | 3000 m² | Giongo & Waechter (2007) |
|  | São Francisco de Paula | RS | 29^o^ 28' S | 50^o^ 13' W | DBH ≥ 10 cm | Plot | 6300 m² | Mello (2006) |
|  | São Francisco de Paula | RS | 29^o^ 28' S | 50^o^ 13' W | All shrub/tree spp. | Plot | 1200 m² | ** |
|  | São Francisco de Paula | RS | 29^o^ 23' S | 50^o^ 23' W | ≥ 2 m tall | Plot | 972 m² | Rosário (2001) |
|  | São Francisco de Paula | RS | 29^o^ 23' S | 50^o^ 23' W | *GBH ≥ 30 cm | Plot | 9990 m² | Longui *et al.* (2006) |
|  | São Francisco de Paula | RS | 29^o^ 23' S | 50^o^ 23' W | ≥ 50 cm tall | Plot | 1050 m² | Fontoura et al. (2006) |
|  | São Francisco de Paula | RS | 29^o^ 23' S | 50^o^ 23' W | DBH ≥ 5 cm | Plot | 2900 m² | Sonego et al. (2007) |
|  | Aratinga | RS | 29^o^ 23' S | 50^o^ 14' W | All shrub/tree spp. | Plot | 1200 m² | ** |
|  | Nova Petrópolis | RS | 29° 22’ S | 51° 05’ W | All shrub/tree spp. with DBH ≥ 5 cm and > 4 m tall | Floristic inventory | ****Na | Grings & Brack (2009) |
|  | Cambará do Sul | RS | 29^o^ 10' S | 50^o^ 07' W | All shrub/tree spp. | Plot | 1200 m² | ** |
|  | Caxias do Sul | RS | 29^o^ 00' S | 50^o^ 55' W | DBH† ≥ 5 cm | Plot | 8000 m² | Rondon Neto *et al.* (2002) |
|  | Nova Prata | RS | 28^o^ 56' S | 51^o^ 53' W | GBH ≥ 30 cm | Plot | 10000 m² | Nascimento *et al.* (2001) |
|  | Vacaria | RS | 28^o^ 40' S | 50^o^ 56' W | >2 m tall | Plot | 2500 m² | Mauhs & Backes (2002) |
|  | Bom Jardim da Serra | SC | 28^o^ 19' S | 49^o^ 33' W | All shrub/tree spp. | Floristic inventory | Na | Eskuche (2007) |
|  | Muitos Capões | RS | 28^o^ 13' S | 51^o^ 10' W | DBH ≥ 5 cm | Plot | 4800 m² | Jarenkow & Baptista (1987) |
|  | Campo Belo do Sul | SC | 28^o^ 00' S | 50^o^ 49' W | DBH ≥ 10 cm | Plot | 9600 m² | Formento *et al.* (2004) |
|  | Faxinalzinho | SC | 27^o^ 20' S | 52^o^ 40' W | GBH ≥ 15 cm | Plot | 10000 m² | Leyser et al. (2009) |
|  | Caçador | SC | 26^o^ 50' S | 50^o^ 55' W | > 60 cm tall | Plot | 10000 m² | Silva *et al.* (1997) |
|  | Caçador | SC | 26^o^ 47' S | 51^o^ 01' W | DBH ≥ 5 cm | Point centered quarter method | 600 points | Negrelle & Silva (1992) |
|  | Misiones | MIS | 26^o^ 30' S | 53^o^ 56' W | DBH ≥ 4.8 cm | Plot | 16400 m² | Ríos et al. (2008) |
|  | Rio do Rastro | SC | 26^o^ 12' S | 51^o^ 06' W | GBH ≥ 15 cm | Plot | 1200 m² | Curcio et al. (2007) |
|  | Araucária | PR | 25^o^ 35' S | 49^o^ 20' W | GBH ≥ 15 cm | Plot | 2000 m² | Carvalho et al. (2009) |
|  | Rio do Rastro | SC | 25^o^ 35' S | 49^o^ 25' W | GBH ≥ 15 cm | Plot | 1200 m² | Curcio et al. (2007) |
|  | São João do Triunfo | PR | 25^o^ 34' S | 50^o^ 05' W | DBH ≥ 10 cm | Plot | 35000 m² | Sanquetta *et al.* (2000) |
|  | São João do Triunfo | PR | 25^o^ 34' S | 50^o^ 05' W | DBH ≥20 cm | Plot | 9000 m² | Schaaf *et al.* (2005) |
|  | Irati | PR | 25^o^ 28' S | 50^o^ 38' W | All shrub/tree spp. | Floristic inventory | Na | Carvalho (1980) |
|  | Fernandes Pinheiro | PR | 25^o^ 27' S | 50^o^ 38' W | DBH ≥ 10 cm | Plot | 18000 m² | Galvão *et al.* (1989) |
|  | Fernandes Pinheiro | PR | 25^o^ 27' S | 50^o^ 38' W | All shrub/tree spp. | Floristic inventory | Na | Carvalho (1980) |
|  | Guarapuava | PR | 25^o^ 21' S | 51^o^ 28' W | DBH ≥ 4.8 cm | Plot | 3200 m² | Cordeiro & Rodrigues (2007) |
|  | Colombo | PR | 25^o^ 20' S | 49^o^ 14' W | DBH ≥ 15 cm | Point centered quarter method | 79 points | Silva & Marconi (1990) |
|  | Colombo | PR | 25^o^ 20' S | 49^o^ 14' W | DBH ≥ 5 cm | Plot | 7200 m² | Oliveira & Rotta (1982) |
|  | Ponta Grossa | PR | 25^o^ 13' S | 50^o^ 15' W | DBH ≥ 15 cm | Point centered quarter method | 75 points | Negrelle & Leuchtenberger (2001) |
|  | Campos Gerais | PR | 25^o^ 02' S | 50^o^ 04' W | All shrub/tree spp. along 15 transects (31-76 m long) | Floristic inventory | Na | Moro et al. (2007) |
|  | Tibagi | PR | 24^o^ 31' S | 50^o^ 25' W | DBH ≥ 5 cm | Plot | 10000 m² | Dias *et al.* (1998) |
|  | Serra de Paranapiacaba | SP | 23^o^ 43' S | 46^o^ 57' W | DBH ≥ 5 cm | Plot | 600 points | Catharino et al. (2006) |
|  | Iperó | SP | 23^o^ 25' S | 47^o^ 35' W | GBH ≥ 15 cm | Plot | 11200 m² | Albuquerque & Rodrigues (2000) |
|  | Campos do Jordão | SP | 22^o^ 42' S | 45^o^ 30' W | All shrub/tree spp. | Floristic inventory | Na | *** |
|  | Camanducaia | MG | 22^o^ 44' S | 45^o^ 55' W | All shrub/tree spp. | Floristic inventory | Na | *** |
|  | Visconde de Mauá | MG | 22^o^ 20' S | 44^o^ 36' W | All shrub/tree spp. | Floristic inventory | Na | Pereira *et al.* (2006) |
|  | Bocaina de Minas | MG | 22^o^ 14' S | 44^o^ 34' W | > 300 cm tall | Plot | 10400 m² | Pereira *et al.* (2006) |
|  | Bocaina de Minas | MG | 22^o^ 13' S | 44^o^ 34' W | DBH ≥ 5 cm | Plot | 10400 m² | Carvalho *et al.* (2005) |
|  | Bocaina de Minas | MG | 22^o^ 10' S | 44^o^ 28' W | > 300 cm tall | Plot | 4000 m² | Pereira *et al.* (2006) |
|  | Santa Rita de Caldas | MG | 22^o^ 05' S | 46^o^ 21' W | DBH ≥ 5 cm | Plot | 10000 m² | Loures et al. (2007) |
|  | Aiuruoca | MG | 22^o^ 01' S | 44^o^ 36' W | All shrub/tree spp. | Floristic inventory | Na | Pereira *et al.* (2006) |
|  | Lavras | MG | 21^o^ 20' S | 44^o^ 58' W | DBH ≥ 5 cm | Plot | 24000 m² | Dalanesi *et al.* (2004) |
|  | Lages | SC | 27° 47' S | 50° 21' W | DBH ≥ 5cm | Plot | 1600m² | Klauberg et al. (2010) |
|  | Urupema | SC | 27° 52' S | 49° 55' W | All shrub/tree spp. | Floristic inventory | Na | Martins-ramos et al. (2010) |
| Dense Forest | Conceição da Barra | ES | 18º 10' S | 39º 53' W | All shrub/tree spp. | Floristic inventory | Na | Pereira & Gomes (1994) |
|  | Linhares | ES | 19º 181 S | 40º 04' W | All shrub/tree spp. | Floristic inventory | Na | Pereira et al. (1998) |
|  | Serra | ES | 20º 07' S | 40º 17' W | All shrub/tree spp. | Floristic inventory | Na | Pereira et al. (2000) |
|  | Vitória | ES | 20º 19' S | 40º 21' W | All shrub/tree spp. | Floristic inventory | Na | Pereira & Assis (2000) |
|  | Vila Velha | ES | 20º 19' S | 40º 21' W | All shrub/tree spp. | Floristic inventory | Na | Pereira & Zambom (1998) |
|  | Guarapari | ES | 20º 36' S | 40º 24' W | DBH ≥ 4.8 cm | Plot | 10000 m² | Assis et al. (2004) |
|  | São João da Barra | RJ | 21º 44' S | 41º 02' W | DHS ≥ 2.5 cm | Plot | 900 m² | Assumpção & Nascimento (2000) |
|  | Campo dos Goytacazes | RJ | 21º 48' S | 44º 40' W | DBH ≥ 3.18 cm | Plot | 6000 m² | Moreno et al. (2003) |
|  | Cachoeira do Macacu | RJ | 22º 28' S | 42º 53' W | DBH ≥ 5 cm | Plot | 150 points | Kurtz & Araújo (2000) |
|  | Silva Jardim | RJ | 22º 32' S | 42º 15' W | DBH ≥ 5 cm | Plot | 10000 m² | Guedes-Bruni et al. (2006) |
|  | Silva Jardim | RJ | 22º 32' S | 42º 16' W | DBH ≥ 5 cm | Plot | 6500 m² | Pessoa & Oliveira (2006) |
|  | Silva Jardim | RJ | 22º 33' S | 42º 15' W | DBH ≥ 3.18 cm | Plot | 7200 m² | Carvalho et al. (2006a) |
|  | Silva Jardim | RJ | 22º 37' S | 42º 28' W | DBH ≥ 5 cm | Plot | 10000 m² | Carvalho et al. (2006b) |
|  | Rio Bonito | RJ | 22º 40' S | 42º 35' W | DBH ≥ 5 cm | Plot | 4000 m² | Carvalho et al. (2007) |
|  | Armação de Buzios | RJ | 22º 46' S | 41º 52' W | DBH ≥ 2.5 cm | Plot | 1000 m² | Lobão & Kurtz (2000) |
|  | Saquarema | RJ | 22º 52' S | 42º 31' W | Na | Floristic inventory | Na | Sá (1992 ) |
|  | Ilha Grande | RJ | 23º 08' S | 44º 11' W | DBH ≥ 2.5 cm | Plot | 2600 m² | Oliveira (2002) |
|  | Ilha Grande | RJ | 23º 10' S | 44º 17' W | DBH ≥ 2.5 cm | Plot | 7500 m² | Araújo et al (1997) |
|  | Picinguaba | SP | 23º 22' S | 44º 48' W | DBH ≥ 4.8 cm | Plot | 5200 m² | Cesar & Monteiro (1995) |
|  | Ubatuba | SP | 23º 22' S | 44º 48' W | DBH ≥ 4.8 cm | Plot | 1800 m² | Lacerda (2001) |
|  | Ubatuba | SP | 23º 22' S | 44º 48' W | DBH ≥ 4.8 cm | Plot | 1800 m² | Lacerda (2001) |
|  | Ubatuba | SP | 23º 22' S | 44º 48' W | DBH ≥ 4.8 cm | Plot | 1000 m² | Assis (1999) |
|  | Ubatuba | SP | 23º 22' S | 44º 48' W | DBH ≥ 4.8 cm | Plot | 1000 m² | Assis (1999) |
|  | Ilha Anchieta, Ubatuba | SP | 23º 33' S | 45º 03' W | DBH ≥ 1.6 cm | Plot | 560 m² | Reis-Duarte (2004) |
|  | Bertioga | SP | 23º 51' S | 46º 08' W | DBH ≥ 3.18 cm | Plot | 4800 m² | Guedes et al. (2006) |
|  | Bertioga | SP | 23º 51' S | 46º 08' W | DBH ≥ 3.18 cm | Plot | 4800 m² | Guedes et al. (2006) |
|  | Sete Barras | SP | 24º 14' S | 48º 04' W | DBH ≥ 5 cm | Plot | 9900 m² | Guilherme et al. (2004) |
|  | Peruíbe | SP | 24º 17' S | 47º 00' W | DBH ≥ 5 cm | Plot | 2000 m² | Oliveira et al. (2001) |
|  | Iguapé | SP | 24º 25' S | 47º 15' W | DBH ≥ 5 cm | Plot | 1000 m² | Carvalhaes (1997) |
|  | Estação Ecológica da Juréia | SP | 24º 30' S | 47º 15' W | DBH ≥ 5 cm | Plot | 10000 m² | Melo et al. (2000) |
|  | Iporanga | SP | 24º 36' S | 48º 37' W | ≥ 2 m tall | Plot | 980 m² | Torezan (1995) |
|  | Pariquera-Açu | SP | 24º 36' S | 47º 53' W | DBH ≥ 4.8 cm | Plot | 12100 m² | Ivanauskas (1997) |
|  | Ilha Comprida | SP | 24º 53' S | 47º 47' W | GBH ≥ 3 cm | Plot | 5000 m² | Silva (2006) |
|  | Cananéia | SP | 25º 03' S | 47º 53' W | DBH ≥ 2.5 cm | Plot | 10000 m² | Melo & Mantovani (1994) |
|  | Quatro Barras | PR | 25º 03' S | 49º 00' W | DBH ≥3.18 cm | Plot | 1000 m² | Portes et al. (2001) |
|  | Ilha do cardoso | SP | 25º 10' S | 47º 58' W | DBH ≥ 2.5 cm | Plot | 2700 m² | Sugiyama (1998) |
|  | Ilha do cardoso | SP | 25º 10' S | 47º 58' W | DBH ≥ 1.5 cm | Plot | 1000 m² | Sugiyama (1998) |
|  | Antonina | PR | 25º 19' S | 48º 42' W | DBH ≥ 4.8 cm | Plot | 1500 m² | Liebsch et al. (2007) |
|  | Superagüi | PR | 25º 22' S | 48º 13' W | DBH ≥ 3.18 cm | Plot | 8100 m² | Jaster (1995) |
|  | Superagüi | PR | 25º 22' S | 48º 13' W | DBH ≥ 3.18 cm | Plot | 7500 m² | Jaster (1995) |
|  | Superagüi | PR | 25º 22' S | 48º 13' W | DBH ≥ 3.18 cm | Plot | 4500 m² | Jaster (19950 |
|  | Morretes | PR | 25º 24' S | 48º 45' W | DBH ≥ 3.18 cm | Plot | 8000 m² | Rocha (1999) |
|  | Morretes | PR | 25º 30' S | 48º 30' W | DBH ≥ 6.3 | Plot | 3800 m² | Guapyassú (1994) |
|  | Ilha do Mel | PR | 25º 30' S | 48º 22' W | DBH ≥ 5 cm | Plot | 3000 m² | Silva (1998) |
|  | Ilha do Mel | PR | 25º 30' S | 48º 22' W | DBH ≥ 5 cm | Floristic inventory | Na | Silva (1998) |
|  | Parque do Palmito | PR | 25º 35' S | 48º 32' W | DBH ≥ 5 cm | Plot | 2800 m² | Rotta et al. (1997) |
|  | Morretes | PR | 25º 36' S | 48º 42' W | DBH ≥ 10 cm | Plot | 4000 m² | Blum (2006) |
|  | Itapoá | SC | 26º 04' S | 48º 38' W | DBH ≥ 5 cm | Plot | 10000 m² | Negrelle (2006) |
|  | Blumenau | SC | 27º 03' S | 49º 04' W | DBH ≥ 4.8 cm | Plot | 4000 m² | Schorn (2005) |
|  | Governador Celso Ramos | SC | 27º 18' S | 48º 33' W | ≥ 1 m tall | Plot | 2500 m² | Citadini-Zanette et al. (2001) |
|  | São Pedro de Alcântara | SC | 27º 34' S | 48º 48' W | ≥ 1 m tall | Plot | 6000 m² | Siminiski et al. (2004) |
|  | Siderópolis | SC | 28º 34' S | 49º 24' W | DBH ≥ 5 cm | Plot | 10000 m² | Martins (2005) |
|  | Criciúma | SC | 28º 48' S | 49º 25' W | DBH ≥ 5 cm | Plot | 10000 m² | Silva (2006) |
|  | Viamao | RS | 30º 05' S | 50º 50' W | DBH ≥ 10 | Point centered quarter method | 60 points | Waechter et al. (2000) |
|  | Palmares | RS | 30º 21' S | 50º 20' W | DBH ≥ 5 cm | Point centered quarter method | 30 points | Moraes & Mondin (2001) |
|  | Lagoa peixe | RS | 31º 10' S | 50º 47' W | DBH ≥ 5 cm | Point centered quarter method | 60 points | Dorneles & Waechter (2004) |
|  | Dom Pedro de alcântara | RS | 29º 23' S | 49º 05' W | GBH ≥ 15 cm | Point centered quarter method | 125 points | Nunes (2001) |
|  | Morrinhos do Sul | RS | 29º 21' S | 49º 58' W | DBH ≥ 5 cm | Plot | 10000 m² | Jarenkow (1994) |
|  | Parque Estadual de Itapeva | RS | 29º 21' S | 49º 45' W | DBH ≥ 2.5 cm | Plot | 1000 m² | Kindel (2002) |
|  | Riozinho | RS | 29º 36' S | 50º 22' W | DBH ≥ 5 cm | Plot | 10000 m² | Brack (2002) |
|  | Maquiné | RS | 29º 42' S | 50º 09' W | DBH ≥ 5 cm | Plot | 10000 m² | Brack (2002) |
|  | São Francisco de Paula | RS | 29º 29' S | 50º 01' W | DBH ≥ 10 cm | Plot | 4200 m² | Mello (2006) |
| Seasonal Forest | Martinho campos | MG | 19° 17'S | 45° 08' W | DBH ≥ 5cm | Plot | 9000m² | Oliveira-Filho et al. (2001) |
|  | Viçosa | MG | 20° 45' S | 42° 07' W | DBH ≥ 5cm | Plot | 10000m² | de Paula et al. (2004) |
|  | Viçosa | MG | 20° 45' S | 42° 52' W | DBH ≥ 5cm | Point centered quarter method | 158 points | da Silva et al. (2003) |
|  | Tiradentes | MG | 21°02' S | 44° 15' W | DBH ≥ 5cm | Plot | 9500m² | Oliveira-Filho (1993) |
|  | Viçosa | MG | 20° 48' S | 42° 51' W | DBH ≥ 5cm | Plot | 2000m² | Ribas et al. (2003) |
|  | fenix | MG | 23° 55' S | 51° 57' W | All shrub/tree spp. | Floristic inventory | Na | Mikich & Silva (2000) |
|  | Viçosa | MG | 20° 45' S | 42° 55' W | All shrub/tree spp. DBH ≥ 5cm | Floristic inventory | Na | Silva et al. (2004) |
|  | Itapeúna | SP | 22° 26' S | 47° 43' W | DBH ≥ 5 cm | Plot | 7850m² | Bertani et al. (2001) |
|  | Bom Sucesso | MG | 21° 09' S | 44° 53' W | DBH ≥ 5 cm | Plot | 11250m² | Carvalho et al. (2005) |
|  | Três Marias | MG | 18° 05' S | 45° 10' W | DBH ≥ 5 cm | Plot | 11250m² | Carvalho et al. (2005) |
|  | Taruma | SP | 22° 42' S | 50° 31' W | DBH ≥ 5 cm | Plot | 3000m² | Durigan (1994) |
|  | Marilha | SP | 22° 01' S | 49° 55' W | DBH ≥ 5 cm | Plot | 3000m² | Durigan (1994) |
|  | Taruma | SP | 22° 49' S | 50° 40' W | DBH ≥ 5 cm | Plot | 3000m² | Durigan (1994) |
|  | Itutinga | MG | 21° 21' S | 44° 36' W | DBH ≥ 5 cm | Plot | 8400m² | Van den Berg & Oliveira-Filho (2000) |
|  | Rio Claro | SP | 22° 35' S | 47° 40' W | GBH ≥15 cm | Plot | 4500 m² | Cardoso-Leite et al. (2004) |
|  | Itutinga | MG | 21° 21' S | 44° 37' W | DBH ≥ 5 cm | Plot | 9000m² | Vilela et al. (1995) |
|  | Araguari | MG | 18° 47' S | 48° 06' W | DBH ≥ 5 cm | Plot | 12000m² | Siqueira et al. (2009) |
|  | Riberão Preto | SP | 21° 13' S | 47° 50' W | DBH≥ 4.8 cm | Plot | 380 points | Tanaka (dissetação) |
|  | Diamante do Norte | PR | 22° 34' S | 54° 49' W | DBH ≥ 5 cm | Plot | 4350m² | Quiqui et al. (2007) |
|  | Viçosa | MG | 20° 45' S | 42° 52' W | All shrub/tree spp. DBH ≥ 3 cm | Floristic inventory | Na | Sobrinho et al. (2009) |
|  | Ilha Solteira | SP | 20° 23' S | 51° 20' W | DBS ≥ 3.2 cm | Plot | 800m² | Silva et al. |
|  | Itatinga | SP | 23° 17' S | 48° 33' W | DBH ≥ 4.8 cm | Plot | 4200m² | Ivanauskas et al. (1999) |
|  | Luminárias | MG | 21° 29' S | 44° 55' W | DBH ≥ 5cm | Plot | 12800m² | Rodrigues et al. (2002) |
|  | Lavras | MG | 21° 13' S | 44° 58' W | DBH ≥ 5 cm | Plot | 11600m² | Machado et al. (2004) |
|  | Jardim | MS | 21° 24' S | 56° 22' W | DBH ≥ 10 cm | Plot | 9000m² | Battilani et al. (2005) |
|  | Viçosa | MG | 20° 45' S | 42° 55' W | DBH ≥ 10 cm | Plot | 10000m² | Meira-Neto & Martins (2002) |
|  | Lavras | MG | 21° 18' S | 44° 20' W | DBH ≥ 5 cm | Plot | 11200m² | Souza et al. (2003) |
|  | Dourados | MS | 22° 13' S | 54° 48' W | GBH ≥ 15 cm | Wandering quarter | 1680 m linear | Arruda & Daniel (2007) |
|  | Pedreira | SP | 22° 47' S | 46° 52' W | All shrub/tree spp. DBH ≥ 3cm | Floristic inventory | Na | Yamamoto et al. (2007) |
|  | Glória de Dourados | MS | 22° 43' S | 53° 18' W | DBH ≥ 5 cm | Plot | 5400m² | Campos et al. (2000) |
|  | Marechal Cândido Rondon | PR | 24° 33' S | 54° 04' W | DBH ≥ 10 cm | Plot | 600m² | Kipper et al. (2010) |
|  | Tomazina | PR | 23° 46' S | 49° 57' W | DBH ≥ 10 cm | Plot | 5600m² | Blum et al. 2003 |
|  | São Domingos | GO | 13° 49' S | 46° 41' W | DBH ≥ 5 cm | Plot | 10000m² | Silva & Scariot (2003) |
|  | São Domingos | GO | 13° 41' S | 46° 44' W | DBH ≥ 5 cm | Plot | 10000m² | Silva & Scariot (2004) |
|  | Uberlândia | MG | 18° 56' S | 48° 12' W | All shrub/tree spp. | Floristic inventory | Na | Rodrigues & Araujo (1997) |
|  | Viçosa | MG | 20° 45' S | 42° 55' W | All shrub/tree spp. | Floristic inventory | Na | Marangon et al. (2003) |
|  | São Martinho da Serra | RS | 29° 32' S | 53° 48' W | GBH ≥ 30 cm | Plot | 2800m² | Scipioni et al. (2009) |
|  | Bauru | SP | 22° 20' S | 49° 00' W | All shrub/tree spp. | Floristic inventory | Na | Pinheiro & Monteiro (2008) |
|  | Brotas | SP | 22° 16' S | 48° 06' W | DBH ≥ 5 cm | Plot | 3600m² | Marques et al. (2003) |
|  | Uberlândia | MG | 18° 56' S | 48° 12' W | DBH≥ 10 cm | Plot | 5000m² | Silva & Araujo (2009) |
|  | Corumbá | MS | 19° 01' S | 57° 40' W | DBH ≥ 5 cm | Point centered quarter method | 78 points | Lima et al. (2010) |
|  | Itatinga | SP | 23° 17' S | 48° 38' W | GBH ≥ 15 cm | Plot | 10000m² | Ivanauskas et al. (1997) |
|  | Piracicaba | SP | 22° 39' S | 47° 39' W | GBH ≥ 15 cm | Plot | 4300m² | Ivanauskas & Rodrigues (2000) |
|  | Itambé do Mato Dentro | MG | 19° 26' S | 43° 14' W | DBH ≥ 5 cm | Plot | 7875m² | Carvalho et al. (2000) |
|  | Viçosa | MG | 20° 45' S | 42° 55' W | DBH ≥ 5 cm | Plot | 5000m² | Silva et al. (2004) |
|  | São Roque | SP | 23° 31' S | 47° 06' W | DBH ≥ 5 cm | Plot | 9450m² | Leite & Rodrigues (2008) |
|  | Cachoeira do Sul | RS | 30° 04' S | 52° 53' W | DBH ≥ 5 cm | Plot | 5800m² | Araujo et al. (2004) |
|  | Piracicaba | SP | 22° 47' S | 47° 49' W | DBH ≥ 5 cm | Plot | 5805m² | Nascimento et al. (1999) |
|  | Dionísio | MG | 19° 48' S | 42° 28' W | DBH ≥ 5 cm | Plot | 10000m² | Camargos et al. (2008) |
|  | Ingaí | MG | 21° 24' S | 44° 55' W | DBH ≥ 5 cm | Plot | 10000m² | Botrel et al. (2002) |
|  | Campinas | SP | 22° 53' S | 47° 04' W | All shrub/tree spp. GBH≥ 15 cm | Floristic inventory | Na | Filho & Santin (2002) |
|  | Santa Teresa | RS | 29° 11’ S | 51° 43' W | DBH ≥ 9.5 cm | Plot | 4000m² | Vaccaro (1991) |
|  | Campinas | SP | 22° 49' S | 47° 06' W | all shrub/tree spp. GBH≥ 15 cm | Floristic inventory | Na | Martins & Rodrigues (2002) |
|  | Corumbá | MS | 19° 05' S | 57° 40' W | DBH ≥ 3 cm | Point centered quarter method | 110 points | Salis et al. (2004) |
|  | Bonito | MS | 21° 21' S | 56° 56' W | All shrub/tree spp. | Floristic inventory | Na | Baptista-Maria et al. (2009) |
|  | Piedade do Rio Grande | MG | 21° 29' S | 44° 06' W | DBH ≥ 5 cm | Plot | 12000m² | Warley et al. (2007) |
|  | Lavras | MG | 21° 13' S | 44° 57' W | DBH ≥ 5 cm | Plot | 50400m² | Nunes et al. (2003) |
|  | Parobé | RS | 29° 41’ S | 50° 51' W | DBH≥ 10 cm | Point centered quarter method | 30 points | Daniel (1991 ) |
|  | Santa Maria | RS | 29° 45' S | 53° 43' W | DBH≥ 10 cm | Plot | 10000m² | Machado & Longhi (1990) |
|  | Turvo | RS | 27° 11' S | 53° 51' W | DBH ≥ 5 cm | Point centered quarter method | 140 pontos quadrante | Ruschel et al. (2007) |
|  | Cristal | RS | 31° 01' S | 51° 56' W | DBH ≥ 5 cm | Plot | 10000m² | Marchi & Jarenkow (2008) |
|  | Jaguari | RS | 29° 30' S | 54° 40' W | DBH≥ 10 cm | Plot | 14000m² | Longhi et al. (2005) |
|  | Parobé | RS | 29° 42' S | 50° 51' W | DBH ≥ 5 cm | Plot | 2600m² | Porto et al. (2008) |
|  | Santa Maria | RS | 29° 43' S | 53° 43' W | DBH ≥ 9.5 cm | Plot | 10500m² | Farias et al. (1994) |
|  | Turvo | RS | 27° 09' S | 53° 53' W | DBH ≥ 5 cm | Plot | 10000m² | Giehl & Jarenkow (2008) |
|  | Santa Maria | RS | 29° 41' S | 53° 47' W | DBH ≥ 5 cm | Plot | 3600m² | Longhi et al. (1999) |
|  | Santa Maria | RS | 29° 43' S | 53° 47' W | DBH ≥ 9.5 cm | Plot | 3200m² | Longhi et al. (2000) |
|  | Santa Maria | RS | 29° 41' S | 53° 47' W | DBH ≥ 5 cm | Plot | 10000m² | Budke et al. (2004) |
|  | Santa Maria | RS | 29° 41' S | 53° 47' W | DBH ≥ 5 cm | Plot | 10000m² | Budke et al. (2005) |
|  | Santa Maria | RS | 29° 38' S | 53° 54' W | DBH ≥ 5 cm | Plot | 10000m² | Giehl et al. (2007) |
|  | Arroio do Padre | RS | 31° 27' S | 52° 28' W | DBH≥ 10 cm | Plot | 10000m² | Souza (2001) |
|  | Bossoroca | RS | 28° 42' S | 54° 53' W | DBH≥ 10 cm | Plot | 7000m² | Boligon et al. (2005) |
|  | Ijuí | RS | 27° 54' S | 54° 45' W | DBH ≥ 9.5 cm | Plot | 12000m² | Vaccaro & Longhi (1995) |
|  | Camaqua | RS | 30° 41' S | 51° 53' W | DBH ≥ 5 cm | Plot | 10000m² | Jurinitz & Jarenkow (2003) |
|  | Canela | RS | 29° 23' S | 50° 41' W | DBH≥ 10 cm | Point centered quarter method | 30 points | Daniel, A. 1991 (dissertação) |
|  | Tupanciretã | RS | 29° 04' S | 53° 50' W | DBH ≥ 5 cm | Plot | 2000m² | Rosa et al. (2008) |
|  | Encruzilhada do Sul | RS | 30° 30' S | 52° 42' W | DBH ≥ 5 cm | Plot | 3000m² | Giongo & Waechter (2007) |
|  | Jaguari | RS | 29° 30' S | 54° 40' W | DBH≥ 10 cm | Plot | 14000m² | Hack et al. (2005) |
|  | Bacia do Rio Jacuí | RS | 30° 00' S | 52° 48' W | DBH ≥ 5 cm | Plot | 10000m² | Lindenmaier & Budke (2006) |
|  | Londrina | RS | 23° 27' S | 51° 15' W | DBH ≥ 5 cm | Plot | 5000m² | Bianchini et al. (2003) |
|  | Monte Negro | RS | 29° 49' S | 51° 25' W | DBH ≥ 5 cm | Plot | 18000m² | Longhi et al. (2008) |
|  | Morro Santana, POA | RS | 30° 01' S | 51° 13' W | All shrub and tree spp. DBH ≥ 3cm | Floristic inventory | Na | Vargas (2005) |
|  | Rolante | RS | 29° 35' S | 50° 26' W | DBH≥ 10 cm | Point centered quarter method | 30 points | Daniel (1991) |
|  | Santiago | RS | 29° 10' S | 54° 51' W | DBH ≥ 9.5 cm | Plot | Na | Longhi (1991) |
|  | Horto de São Pedro (Baixo Jacuí) | RS | 30° 03' S | 51° 43' W | DBH≥ 10 cm | Plot | 2400m² | Balbueno & Oliveira (2000) |
|  | Horto Santa Rosa (Baixo Jacuí) | RS | 30° 11' S | 51° 45' W | DBH≥ 10 cm | Plot | 2400m² | Balbueno & Oliveira (2000) |
|  | São Sepé | RS | 30° 10' S | 53° 34' W | DBH≥ 10 cm | Plot | 2500m² | Longhi et al. (1992) |
|  | Vale do Sol | RS | 29° 34' S | 52° 40' W | DBH ≥ 5cm and high ≥ 5m | Plot | 10000m² | Jarenkow & Waechter (2001) |
|  | Alinópolis | MG | 20° 41' S | 46° 22' W | DBH ≥ 5 cm | Plot | 4800m² | Fagundes et al. (2007) |
|  | Passos | MG | 20° 40' S | 46° 27' W | DBH ≥ 5 cm | Plot | 5200m² | Fagundes et al. (2007) |
|  | Gália | SP | 22° 24' S | 49° 42' W | DBH ≥ 5 cm | Plot | 6000m² | Durigan et al. (2000) |
|  | Itapiranga | SC | 27° 11' S | 53° 38' W | DBH ≥ 5 cm | Plot | 11200m² | Ruschel et al. (2009) |
|  | São Valentim | SC | 26° 56' S | 53° 31' W | DBH ≥ 5 cm | Plot | 11200m² | Ruschel et al. (2009) |
|  | São Paulo | SP | 23° 34' S | 46° 43' W | GBH ≥ 50 cm | Plot | 7000m² | Dislich et al. (2001) |
|  | Araguari | MG | 18° 48' S | 48° 07' W | DBH ≥ 5 cm | Plot | 12000m² | Siqueira et al. 2009 |
|  | Cabo Frio | RJ | 22° 48' S | 41° 57' W | DBH ≥ 5 cm | Plot | 5000m² | Kurtz et al. (2009) |
|  | Goyatacazes | RJ | 21° 24' S | 41° 04' W | DBH≥ 10 cm | Plot | 10000m² | Silva & Nascimento (2001) |
|  | Goyatacazes | RJ | 21° 42' S | 41° 15' W | DBH >3.2 cm | Point centered quarter method | 84 points | Carvalho et al. (2006) |
|  | São Domingos | RJ | 21° 21' S | 41° 56' W | DBH ≥ 5 cm | Plot | 8000m² | Dan et al. (2010) |

| *GBH: Girth at breast height |
| --- |
| †DBH: Diameter at breast height |
| ** data kindly provided by Rodrigo Bergamin |
| *** data kindly provided by Ary T. Oliveira-Filho |
| **** Not available |

**References**

Albuquerque, G.B., and Rodrigues, R.R. 2000. A vegetação do Morro de Araçoaiaba, Floresta Nacional de Ipanema, Iperó (SP). Scientia Forestalis 58:145–159.

Araújo, D.S.D., Oliveira, R.R., Lima, E. e Ravelli, A. 1997. Estrutura da vegetação e condições edáficas numa clareira de mata de restinga na Reserva Biológica Estadual da Praia do Sul, RJ. Brazilian Journal of Ecology 1:36-43.

Araújo, M.M., Longhi, S.J., Brena, D.A., Barros, P.L.C. e Franco, S., 2004. Análise de agrupamento da vegetação de um fragmento de Floresta Estacional Decidual Aluvial, Cachoeira do Sul, RS, Brasil. Ciência Florestal 14(1), 133-147.

Arruda, L., Daniel, O., 2007. Florística e diversidade em um fragmento de floresta estacional semidecidual aluvial em dourados. MS. Floresta, 37, 2.

Assis, A.M., Pereira, O.J. e Thomaz, L.D. 2004. Fitossociologia de uma floresta de restinga no Parque Estadual Paulo César Vinha, Setiba, município de Guarapari (ES). Rev. Bras. Bot. 27:349-361.

Assis, M.A. 1999. Florística e caracterização das comunidades vegetais da planície costeira de Picinguaba, Ubatuba – SP. PhD Thesis, Instituto de Biologia da Universidade Estadual de Campinas.

Assumpção, J. e Nascimento, M.T. 2000. Estrutura e composição florística de quatro formações vegetais de restinga no complexo lagunar Grussaí/Iquipari, São João da Barra, RJ, Brasil. Acta bot Bras 14:243-357.

Balbueno, R.A., Oliveira, P.L., 2000. Estrutura e composição florística de dois fragmentos florestais na região do Baixo Jacuí, RS, Brasil. Biotemas 13 (2), 23-46.

Baptista-Maria, V.R., Rodrigues, R.R., Junior, G.D., Maria, F.S. e Souza, V.C. 2009. Composição florística de florestas estacionais ribeirinhas no Estado de Mato Grosso do Sul, Brasil. Acta bot. bras. 23(2), 535-548.

Battilani, J.L., Scremin-Dias, E. e Souza, A.L.T., 2005. Fitossociologia de um trecho da mata ciliar do rio da Prata, Jardim, MS, Brasil1 , Acta bot. bras. 19(3), 597-608.

Bertani, D.F., Rodrigues, R.R., Batista, J.L.F. e george john Shepherd, G.J. 2001. Análise temporal da heterogeneidade florística e estrutural em uma floresta Ribeirinha. Revista brasil. Bot., São Paulo, 24(1), 11-23.

Bianchini, E., Popolo, R.S., Dias, M.C. e Pimenta, J.A., 2003. Diversidade e estrutura de espécies arbóreas em área alagágel do município de Londrina, sul do Brasil. Acta bot. bras. 17(3), 405-419.

Blum, C.T. 2006. A floresta ombrófila densa na Serra da Prata, Parque Nacional Saint-Hilare/Lange, PR – Caracterização florística, fitossociológica e ambiental de um gradiente altitudinal. MSc Dissertation, Setor de Ciências Agrárias da Universidade Federal do Paraná.

Blum, C.T., Silva, D.A.T., Hase, L.M., Miranda, D.L.C. 2003. Caracterização florística e ecológica de remanescentes florestais no Rio Das Cinzas, Norte Pioneiro, PR. UFPR, Curitiba, PR.

Boligon, A.A., Longhi, S.J., Murari, A.B. e Hack, C., 2005. Aspectos fitossociológicos de um fragmento da floresta natural de Astronium balasae engl., no município de Bossoroca, RS.Ciência rural, Santa Maria, 35(5).

Borghi, W.A., Martins, S.S., Quiqui, E.M.D., Nanni, M.R., 2004. Caracterização e avaliação da mata ciliar à montante da Hidrelétrica de Rosana, na Estação Ecológica do Caiuá, Diamante do Norte, PR, Cad. biodivers. 4(2).

Botrel, R.T., Oliveira Filho, A.T., Rodrigues, L.A. e Curi, N., 2002. Influência do solo e topografia sobre as variações da composição florística e estrutura da comunidade arbóreo-arbustiva de uma floresta estacional semidecidual em Ingaí, MG , Revista Brasil. Bot., 25(2), 195-213.

Budke, J.C., Athayde, E.A., Giehl, E.L.H., Záchia, R.A. & Eisinger, S.M., 2005. Composição florística e estratégias de dispersão de espécies lenhosas em uma floresta ribeirinha, Arroio Passo das Tropas, RS. Iheringia, Sér. Bot., Porto Alegre, 60(1), 17-24.

Budke, J.C., Giehl, E.L., Athayde, E.L.H., Eisinger, S.M., Záchia, R.A., 2004. Florística e fitossociologia do componente arbóreo de uma floresta ribeirinha, arroio Passo das Tropas, Santa Maria, RS, Brasil. Acta bot. bras. 18(3), 581-589.

Camargos, V.L., Silva, A.F., Neto, J.A.A.M., e S.V., 2008. Influência de fatores edáficos sobre variações florísticas na Floresta Estacional Semidecídua no entorno da Lagoa Carioca, Parque Estadual do Rio Doce, MG, Brasil , Acta bot. bras. 22(1), 75-84.

### **Campos, J.B., Romagnolo, M.B. e Souza, M.C., 2000. Structure, composition and spatial distribution of tree species in a remnant of the semideciduous seasonal Alluvial Forest of the upper Paraná River Floodplain.** Braz. arch. biol. technol. 43(2).

Cardoso-Leite, E., Covre, T.B., Ometto, R.G., Cavalcanti, D.C. e Pagani, M.I., 2004. Fitossociologia e caracterização sucessional de um fragmento de mata ciliar, em Rio Claro/SP, como subsídio à recuperação da área. Rev. Inst. Flor., São Paulo, 16(1), 31-41.

Cardoso-Leite, E., Rodrigues, R.R., 2008. Fitossociologia e caracterização sucessional de um fragmento de floresta estacional no sudeste do brasil. Revista Árvore, Viçosa-MG, 32(3), 583-595.

Carvalhaes, M.A. 1997. Florística e estrutura de Mata sobre restinga na Juréia, Iguape, SP. MSc Dissertation, Departamento de Ecologia Geral da Universidade de São Paulo.

Carvalho, D.A., Oliveira Filho, A.T., Vilela, E.A., Curi, N., Van den Berg, E., Fontes, M.A. e Botezelli, L., 2005. Distribuição de espécies arbóreo arbustivas ao longo de um gradiente de solos e topografia em um trecho de floresta ripária do Rio São Francisco em Três Marias, MG, Brasil , Revista Brasil. Bot., 28(2), 329-345.

Carvalho, D.A., Oliveira-Filho, A.T., van der Berg, E., Fontes, M.A.L., Vilela, E.A., Marques, J.J.G.S.M., and Carvalho, W.A.C. 2005. Variações florísticas e estruturais do componente arbóreo de uma floresta ombrófila alto-montana às margens do rio Grande, Bocaina de Minas, MG, Brasil. Acta Botanica Brasilica 19:91–109.

### **Carvalho, D.A., Oliveira-Filho, A.T., Vilela, E.A. e Curi, N., 2000. Florística e estrutura da vegetação arbórea de um fragmento de floresta semidecedual às margens do reservatório da usina hidrelétrica Dona Rita (Itambé do Mato Dentro, MG)** Acta Bot. Bras. 14(1).

Carvalho, F.A., Braga, J.M.A., Gomes, J.M.L., Souza, J.S. e Nascimento, M.T. 2006. Comunidade arbórea de uma floresta de baixada aluvial no município de Campos dos Goytacazes, RJ. Cerne, Lavras, v. 12, n. 2, p. 157-166, abr./jun.

Carvalho, F.A., Nascimento, M.T., Braga, J.M.A. e Rodrigues, P.J.F.P. 2006. Estrutura da comunidade arbórea da Floresta Atlântica de Baixada periodicamente inundada da Reserva Biológica de Poço das Antas, Rio de Janeiro, Brasil. Rodriguésia 57:503-518.

Carvalho, J., Marques, M.C.M., Roderjan, C.V., Barddal, M., and Sousa, S.G.A. 2009. Species distribution relationships of different strata and soil characteristics in an alluvial forest in Paraná State, Brazil. Acta Botanica Brasilica 23:1-9.

Carvalho, P.E.R. 1980. Levantamento florístico da região de Irati – PR (1ª aproximação). EMBRAPA, Curitiba.

Carvalho, W.A.C., Oliveira Filho,A.T., Fontes, M.A.L. e Curi, N. 2007. Variação espacial da estrutura da comunidade arbórea de um fragmento de floresta semidecídua em Piedade do Rio Grande, MG, Brasil , Revista Brasil. Bot., V.30, n.2, p.315-335, abr.-jun.

Catharino, E.L.M., Bernacci, L.C., Franco, G.A.D.C., Durigan, G., and Metzger, J.P. 2006. Aspectos da composição e diversidade do componente arbóreo das florestas da Reserva Florestal do Morro Grande, Cotia, SP. Biota Neotropica 6:1-28.

César, O. e Monteiro, R. 1995. Florística e fitossociologia de uma floresta de restinga em Picinguaba (Parque Estadual da Serra do Mar), município de Ubatuba-PR. Naturalia 20:89-105.

Cielo-Filho, R. e Santin, D. 2002. Estudo florístico e fitossociológico de um fragmento florestal urbano: Bosque dos Alemães, Campinas, SP. Rev. bras. bot. 25(3):291-301.

Cordeiro, J., and Rodrigues, W.A. 2007. Caracterização fitossociológica de um remanescente de floresta ombrófila mista em Guarapuava, PR. Árvore 31:545-554.

Curcio, G.R., Galvão, F., Bonnet, A., Barddal, M.L., and Dedecek, R.A. 2007. A floresta fluvial em dois compartimentos do rio Iguaçu, Paraná, Brasil. Floresta 37:125-147.

Dalanesi, P.E., Oliveira-Filho, A.T., and Fontes, M.A.L. 2004. Flora e estrutura do componente arbóreo da floresta do Parque Ecológico Quedas do Rio Bonito, Lavras, MG, e correlações entre a distribuição das espécies e variáveis ambientais. Acta Botanica Brasilica 18:737–757.

Dan, M.L., Braga, J.M.A. e Nascimento, M.T. 2010. Estrutura da comunidade arbórea de fragmentos de floresta Estacional Semidecidual na bacia hidrográfica do rio São Domingos, Rio de Janeiro, Brasil Rodriguésia 61(4): 749-766.

Daniel, A. 1991. Estudo fitossociológico arbóreo/ arbustivo da mata ripária da bacia hidrográfica do rio dos sinos, RS. Pesquisas, série botânica 41: 5-199.

De Paula, A., Silva, A.F., Júnior, P.M., Santos, F.A.M. e Souza, A.L. 2004. Sucessão ecológica da vegetação arbórea em uma Floresta Estacional Semidecidual, Viçosa, MG, Brasil1 , Acta bot. bras. 18(3): 407-423.

### **Del Quiqui, E.M., Martins, S.S., Silva, I.C., Borghi, W.A., Silva, O.H., Sakuragui, C.M. e Pacheco, R.B. 2007.** Estudo fitossociológico de um trecho da floresta estacional semidecidual em Diamante do Norte, Estado do Paraná, Brasil- DOI: 10.4025/actasciagron.v29i2.298 Acta Sci. Agron. Maringa, v. 29, n. 2, p. 283-290.

Dias, H.M. 2005. Estrutura do estrato lenhoso de uma comunidade arbustiva fechada sobre cordão arenoso na Restinga de Marambaia, RJ. Msc Dissertation, Escola Nacional de Botânica Tropical do Instituto de Pesquisas Jardim Botânico do Rio de Janeiro.

Dias, M.C., Vieira, A.O.S., Nakajima, J.N., Pimenta, J.A., and Lobo, P.C. 1998. Composição florística e fitossociologia do componente arbóreo das florestas ciliares do rio Iapó, na bacia do rio Tibagi, Tibagi, PR. Revista Brasileira de Botânica 21:183-195.

Dislich, R., Cersósimo, L. e Mantovani, W. 2001. Análise da estrutura de fragmentos florestais no Planalto Paulistano - SP. Revta brasil. Bot., São Paulo, V.24, n.3, p.321-332.

Dorneles, L.P.P.e Waechter, J.L. 2004. Estrutura do componente arbóreo da floresta arenosa de restinga do Parque Nacional da Lagoa do Peixe, Rio Grande do Sul. Hoehnea 31:61-71.

Durigan, G. 1994. Floristica, fitossociologia e produção de folhedo em matas ciliares da região oeste do Estado de São Paulo. Tese de Doutorado, Universidade Estadual de Campinas.

Durigan, G., Franco, G.A.D.C., Saito, M. e Baitello, J.B. 2000. Estrutura e diversidade do componente arbóreo da floresta na Estação Ecológica dos Caetetus, Gália, SP. Revista Brasileira de Botânica 23(4).

Eskuche, U. 2007. El bosque de Araucaria com Podocarpus y los campos de Bom Jardim da Serra, Santa Catarina (Brasil Meridional). Boletín de la Sociedad Argentina de Botánica 42:295-308.

Fabris, L.C. e César, O. 1996. Estudos florísticos em uma mata litorânea no sul do estado do Espírito Santo, Brasil. Boletim do Museu de Biologia Mello Leitão 5:15-46.

Fagundes, L.M., Carvalho, D.A., Van den Berg, E., Marques, J.J.G.S.M. e Machado, E.L.M. 2007. Florística e estrutura do estrato arbóreo de dois fragmentos de florestas decíduas às margens do rio Grande, em Alpinópolis e Passos, MG, Brasil. Acta botanica Brasilica 21(1).

Farias, J.A.C.; Teixeira, I.F.; Pes, L. & Filho. A.A. 1994. Estrutura fitossociológica de uma floresta estacional decidual na região de Santa Maria, RS. Santa Maria: Ciência Florestal, 4 (1): 109-128.

Fontoura, S.B., Ganade, G., and Larocca, J. 2006. Changes in plant community diversity and composition across an edge between Araucaria forest and pasture in South Brazil. Revista Brasileira de Botânica 29:79-91.

Formento, S., Schorn, L.A., and Ramos, R.A.B. 2004. Dinâmica estrutural arbórea de uma floresta ombrófila mista em Campo Belo do Sul, SC. Cerne 10:196–212.

Galvão, F., Kuniyoshi, Y.S., and Roderjan, C.V. 1989. Levantamento fitossociológico das principais associações arbóreas da Floresta Nacional de Irati – PR. Floresta 1/2:30–49.

Galvão, F., Roderjan, C.V., Kuniyoshi, Y.S. e Ziller, S.R. 2002. Composição florística e fitossociologia de caxetais do litoral do Paraná – Brasil. Floresta 32:17-39.

Giehl, E.L.H. e Jarenkow, J.A. 2008. Gradiente estrutural no componente arbóreo e relação com inundações em uma floresta ribeirinha, rio Uruguai, sul do Brasil. Acta bot. bras. 22(3): 741-753.

Giehl, E.L.H., Athayde, E.A., Budke, J.C., Gesing, J.P.A., Einsiger, S.M. e Canto-Dorow, T.S. 2007. Espectro e distribuição vertical das estratégias de dispersão de diásporos do componente arbóreo em uma floresta estacional no sul do Brasil. Acta bot. bras. 21(1): 137-145.

Giongo, C. e Waechter, J.L. 2007. Composição florística e espectro de dispersão das espécies arbóreas de uma floresta mista com Podocarpus, Rio Grande do Sul. Nota científica. Revista Brasileira de Biociências, Porto Alegre, v. 5.

Giongo, C., and Waechter, J.L. 2007. Composição florística e espectro de dispersão das espécies arbóreas de uma floresta mista com Podocarpus, rio Grande do Sul. Revista Brasileira de Biociências 5:333-335.

Guapyassú, M.S., 1994. Caracterização fitossociológica de três fases sucessionais de uma Floresta Ombrófila Densa Submontana Morretes - Paraná. MSc Dissertation, Setor de Ciências Agrárias da Universidade Federal do Paraná.

Guedes-Bruni, R.R., Silva Neto, S.J., Morim, M.P. e Mantovani, W. 2006. Composição florística e estrutura de trecho de Floresta Ombrófila Densa Atlântica Aluvial na Reserva Biológica de Poço das Antas, Silva Jardim, Rio de Janeiro, Brasil. Rodriguésia 57:413-428.

Guedes, D., Barbosa, L.M. e Martins, S.E. 2006. Composição florística e estrutura fitossociológica de dois fragmentos de floresta de restinga no município de Bertioga, SP, Brasil. Acta bot Bras 20:299-311.

Guilherme, F.A.G., Morellato, L.P.C. e Assis, M.A. 2004. Horizontal and vertical tree community structure in a lowland Atlantic Rain Forest, Southeastern Brazil. Rev. Bras. Bot. 27:725-737.

Hack, C., Longhi, S.J., Boligon, A.A., Murari, A.B. e Pauleski, D.T. 2005. Análise fitossociológica de um fragmento de floresta estacional decidual no município de Jaguari, RS. Ciência Rural, Santa Maria, v35.

Ivanauskas, N.M. 1997. Caracterização florística e fisionomia da Floresta Atlântica sobre a formação Pariquera-Açu, na Zona da Morraria Costeira do estado de São Paulo. Msc Dissertation, Instituto de Biologia da Universidade Estadual de Campinas.

### **Ivanauskas, N.M. e Rodrigues, R.R. 2000. Florística e fitossociologia de remanescentes de floresta estacional decidual em Piracicaba, São Paulo, Brasil** Rev. bras. Bot. vol.23 no.3 São Paulo Sept.

Ivanauskas, N.M.,  Rodrigues, R.R.,  Nave, A.G. 1999. Fitossociologia de um trecho de Floresta Estacional Semidecidual em Itatinga , São Paulo , Brasil Phytosociology of the Semi-deciduous Seasonal Forest fragment in Itatinga , São Paulo , Brazil Scientia (1999) Volume: 56, Pages: 83-99

Ivanauskas, N.M., Rodrigues, R.R. e Nave, A.G. 1997. Aspectos ecológicos de um trecho de floresta de brejo em Itatinga, SP: florística, fitossociologia e seletividade de espécies Revta brasil. Bot., São Paulo, V.20, n.2, p.139-153, dez.

Jarenkow, J.A. & Waechter, J.L. 2001. Composição, estrutura e relações florísticas do componente arbóreo de uma Floresta Estacional no Rio Grande do Sul, Brasil. São Paulo: Revista Brasileira de Botânica, 24 (3): 263-272.

Jarenkow, J.A., and Baptista, L.R.M. 1987. Composição florística e estrutura da mata com araucária na estação ecológica de Aracuri, Esmeralda, RS. Napaea 3:9–18.

Jaster, C.B. 2002. A estrutura como indicadora do nível de desenvolvimento sucessional de comunidades arbóreas da restinga – uma proposta metodológica. PhD Thesis, Setor de Ciências Agrárias da Universidade Federal do Paraná.

Jurinitz, C.F. e Jarenkow, J.A. 2003. Estrutura do componente arbóreo de uma floresta estacional na Serra do Sudeste, Rio Grande do Sul, Brasil. Revista Brasil. Bot., V.26, n.4, p.475-487.

Kipper, J., Chambó, E.D., Stefanello, S. e Garcia, R.C. 2010. Levantamento florístico de um componente arbóreo de mata ciliar do Rio Paraná, Marechal Cândido Rondon, PR Scientia Agraria Paranaensis; Vol. 9, No 1 (2010); p.82-92

Kurtz, B.C. e Araújo, D.S.D. 2000. Composição florística e estrutura do componente arbóreo de um trecho de Mata Atlântica na Estação Ecológica Estadual do Paraíso, Cachoeiras de Macacu, Rio de Janeiro, Brasil. Rodriguésia 51:69-112.

Kurtz, B.C., Sá, C.F.C. e Daniele Oliveira da Silva, D.O. 2009. Fitossociologia do componente arbustivo-arbóreo de Florestas Semidecíduas costeiras da região de Emerenças, Área de proteção ambiental do Pau Brasil, Armação dos Búzios, Rio de Janeiro, Brasil. Rodriguésia 60 (1): 129-146.

Lacerda, M.S. 2001. Composição florística e estrutura da comunidade arbórea num gradiente altitudinal da Mata Atlântica. PhD Thesis, Instituto de Biologia da Universidade Estadual de Campinas.

Leite, E.C., e Rodrigues, R.R. 2008. Fitossociologia e caracterização sucessional de um fragmento de floresta estacional no Sudeste do Brasil. R. Árvore, Viçosa-MG, v.32, n.3, p.583-595.

Leyser, G., Viniski, M., Donida, A.L., Zanin, E.M., and Budke, J.C. 2009. Espectro de dispersão em um fragmento de transição entre floresta ombrófila mista e floresta estacional na região do Alto Uruguai, Rio Grande do Sul, Brasil. Pesquisas Série Botânica 60:355-366.

Liebsch, D., Goldenberg, R. e Marques, M.C.M. 2007. Florística e estrutura de comunidades vegetais em uma cronoseqüência de Floresta Atlântica no Paraná. Acta bot Bras 21:983-992.

Lima, M.S., Damasceno-Júnior, G.A. e Tanaka, M.O. 2010. Aspectos estruturais da comunidade arbórea em remanescentes de floresta estacional decidual, em Corumbá, MS, Brasil1 , Revista Brasil. Bot., V.33, n.3, p.437-453, jul.-set.

Lindenmaier, D.S. e Budke, J.C. 2006. Florística, diversidade e distribuição espacial das espécies arbóreas em uma floresta estacional na bacia do rio Jacuí, sul do Brasil, Pesquisas, Botânica N° 57: 193-216. São Leopoldo, Instituto Anchietano de Pesquisas.

Lobão, A.Q. e Kurtz, B.C. 2000. Fitossociologia de um trecho de mata de restinga na Praia Gorda, município de Armação de Búzios, RJ. In: Anais do 5º Simpósio sobre Ecossistemas Brasileiros 3:66-73.

Longhi, S.J. 1991. Aspectos Fitossociológicos de "Capões" na Região de Carovi e Tupantuba, em Santiago, RS. Ci. Flor., Santa Maria, v. 1, n. 1, p. 22-39.

Longhi, S.J., Araujo, M.M., Kelling, M.B., Hoppe, J.M.., Müller, I. & Borsoi, G.A. 2000. Aspectos fitossociológicos de fragmento de floresta estacional decidual. Santa Maria, RS. Santa Maria: Ciência Florestal, 10 (2): 59 - 74.

Longhi, S.J., Boligon, A.A., Murari, A.B., Hack, C., Pauleski, D.T. 2005. Análise florística e estrutural de um fragmento florestal no município de Jaguari - RS. Ciência Florestal v.35, n.5: 1083-1091.

Longhi, S.J., Brena, D.A., Gomes, J.F., Narvaes, I.S., Berger, G., and Soligo, A.J. 2006. Classificação e caracterização de estágios sucessionais em remanescentes de floresta ombrófila mista na Flona de São Francisco de Paula, RS, Brasil. Ciência Florestal 16:113–125.

Longhi, S.J., Brena, D.A., Scipioni, M. C., Giacomolli, L.Z., Deliberali, G., Longhi, R.V. e Mastella, T. 2008. Caracterização fitossociológica do estrato arbóreo em um remanescente de Floresta Estacional Semidecidual, em Montenegro, RS. Ciência Rural, Santa Maria, v.38, n.6, p.1630-1638, set.

Longhi, S.J., Nascimento, A.R.T., Fleig, F.D., Della-Flora, J.B., Freitas, R.A., Charão, L.W. 1999. Composição florística e estrutura da comunidade arbórea de um fragmento florestal no município de Santa Maria - Brasil. Ciência Florestal, Santa Maria, v.9, n.1.

Longhi, S.J.; Selle, G.L.; Ragagnin, L.I.M. & Damiani, J.E. 1992. Composição florística e estrutura fitossociológica de um "capão" de Podocarpus lambertii Klotz., no Rio Grande do Sul. Santa Maria: Ciência Florestal, 2 (1): 9 - 26.

Loures, L., Carvalho, D.A., Machado, E.L.M. e Marques, J.J.G.S.M. 2007. Florística, estrutura, e características do solo de um fragmento de floresta paludosa no sudese do Brasil. Acta Botanica Brasilica 21:885-896.

Machado, E.L.M., Oliveira-Filho, A.T., Carvalho, W.A.C., Souza, J.S., Borém, R.A.T. e Botezelli, L. 2004. Análise comparativa da estrutura e flora do compartimento arbóreo-arbustivo de um remanescente florestal na fazenda Beira Lago, Lavras, MG. Revista Árvore, Viçosa-MG, v.28, n.4, p.499-516.

Machado, P. F. S. Longhi, S.J. 1990. Aspectos floristicos e fitossociologicos do "Morro do Elefante" Santa Maria, RS. Revista Centro de Ciências Rurais, Santa Maria, 20(3-4):261-280.

Marangon, L.C., Soares, J.J. e Feliciano, A.L.P. 2003. Florística arbórea da Mata da Pedreira, município de Viçosa, Minas Gerais. Revista Árvore, Viçosa-MG, v.27, n.2, p.207-215.

Marchi, T.C. 2005. Estudo do componente arbóreo de mata ribeirinha no rio Camaquã, Cristal, RS. Dissertação, UFRGS.

Marchi, T.C. Jarencow, J.A. 2008. Estrutura do componente arbóreo de mata ribeirinha no rio Camaquã, município de Cristal, Rio Grande do Sul, Brasil. Iheringia, Sér. Bot., Porto Alegre, v. 63, n. 2, p. 241-248, jul./dez.

Marques, M.C.M., Silva, S.M. e Salino, A. 2003. Florística e estrutura do componente arbustivo-arbóreo de uma Floresta Higrófila da Bacia do rio Jacaré-Pepira, SP, Brasil. Acta bot. bras. 17(4): 495-506.

Martins, S.V. e Rodrigues, R.R. 2002. Gap-phase regeneration in a semideciduous mesophytic forest, south–eastern Brazil. Plant Ecology 00: 1–12.

Mauhs, J., and Backes, A. 2002. Estrutura fitossociológica e regeneração natural de um fragmento de floresta ombrófila mista exposto a perturbações antrópicas. Pesquisas Série Botânica 52:89-109.

Meira-Neto, J.A.A. e Martins, F.R. 2002. Composição florística de uma Floresta Estacional Semidecidual Montana no município de Viçosa-MG. Revista Árvore, Viçosa-MG, v.26, n.4, p.437-446.

Meira-Neto, J.A.A., Martins, F.R. e Souza, A.L. 2005. Influência da cobertura e do solo na composição florística do sub-bosque em uma floresta estacional semidecidual em Viçosa, MG, Brasil1 , Acta bot. bras. 19(3): 473-486.

Mello, R.S.P. 2006. Detecção de padrões de coexistência arbórea e processos ecológicos em zona de contato de florestas ombrófilas montanas no sul do Brasil. Thesis. Universidade Federal do Rio Grande do Sul, Porto Alegre, Rio Grande do Sul, Brazil.

Melo, M.M.R.F. e Mantovani, W. 1994. Composição florística e estrutura de trecho de Mata Atlântica de encosta na Ilha do Cardoso (Cananéia, SP, Brasil). Boletim do Instituto de Botânica 9:107-158.

Melo, M.M.R.F., Oliveira, R.J., Rossi, L., Mamede, M.C.H. e Cordeiro, I. 2000. Estrutura de um trecho de Floresta Atlântica de planície na Estação Ecológica Juréia-Itatins, Iguape, SP, Brasil. Hoehnea 27:299-322.

Mikich, S.B. e Silva, S.M. 2001. Composição florística e fenologia das espécies zoocóricas de remanescentes de floresta estacional semidecidual no Centro-Oeste do Paraná, Brasil. Acta bot. bras. 15(1): 89-113.

Montezuma, R.C.M. 1997. Estrutura da vegetação de uma restinga de Ericaceae no município de Carapebus – RJ. MSc Dissertation, Departamento de Ecologia da Universidade Federal do Rio de Janeiro.

Moraes, D. e Mondin, C.A. 2001. Florística e fitossociologia do estrato arbóreo em mata arenosa no Balneário do Quintão, Palmares do Sul, Rio Grande do Sul. Pesquisas-Botânica 51:87-100.

Moreno, M.R., Nascimento, M.T. e Kurtz, B.C. 2003. Estrutura e composição florística do estrato arbóreo em duas zonas altitudinais na Mata Atlântica de encosta da Região do Imbé, RJ. Acta bot Bras 17:371-386.

Moro, R.S., Silva, M.A., Dalazoana, K., and Almeida, C.G. 2007. Perfil arbóreo e herbáceo-arbustivo de capões no Parque Nacional dos Campos Gerais, Ponta Grossa, PR.. Revista Brasileira de Biociências 5:126-128.

Nascimento, A.R.T., Longhi, S.J., and Brena, D.A. 2001. Estrutura e padrões de distribuição espacial de espécies arbóreas em uma floresta ombrófila mista em Nova Prata, RS. Ciência Florestal 11:105-119.

### Nascimento, H. E. M., Dias, A.S., Tabanez, A. A. J. e Viana, V. M. 1999. Estrutura e dinâmica de populações arbóreas de um fragmento de floresta estacional semidecidual na região de Piracicaba, SP. Rev. Brasil. Biol., 59(2): 329-342.

Negrelle, R.A.B., and Leuchtenberger, R. 2001. Composição e estrutura do componente arbóreo de um remanescente de floresta ombrófila mista. Floresta 31:42-51.

Negrelle, R.A.B., and Silva, F.C. 1992. Fitossociologia de um trecho de floresta com Araucaria angustifolia (Bert.) O. Ktze. no Município de Caçador – SC. Boletim de Pesquisa Florestal 24/25:37-54.

Negrelle, R.R.B. 2006. Composição florística e estrutura vertical de um trecho de Floresta Ombrófila Densa de planície quaternária. Hoehnea 33:261-289.

Nunes, J.A.A. 1998. Caracterização estrutural, fisionômica e florística da vegetação de restinga no complexo lagunar Grussaí/Iquipari, São João da Barra, RJ. PhD Thesis, Centro de Biociência e Biotecnologia da Universidade Estadual do Norte Fluminense.

Nunes, Y.R.F., Mendonça, A.V.R., Botezelli, L., Machado, E.L.M. e Oliveira-Filho, A.T. 2003. Variações da fisionomia, diversidade e composição de guildas da comunidade arbórea em um fragmento de Floresta Semidecidual em Lavras, MG. Acta bot. bras. 17(2): 213-229.

Oliveira-Filho, A. T., [Carvalho, W. A. C.](http://lattes.cnpq.br/8343479823138548), [Fontes, M. A. L.](http://lattes.cnpq.br/6809021495935047), [Schiavini, I.](http://lattes.cnpq.br/8256858019626093) and 2006. Composição florística do compartimento arbóreo de cinco remanescentes florestais do maciço do Itatiaia, Minas Gerais e Rio de Janeiro. Rodriguesia 57:103-126.

### **Oliveira-Filho, A.T. e Machado, J.N.M. 1993. Composição florística de uma floresta semidecídua montana na Serra de São José, Tiradentes, Minas Gerais** Acta Bot. Bras. vol.7 no.2 Feira de Santana Dec.

Oliveira-filho, A.T., Curi, N., Vilela, E. A. e Carvalho, D. A. 2001. Variation in tree community composition and structure with changes in soil properties within a fragment of Semideciduous Forest in South-Eastern Brazil. Edinb. J. Bot. 58 (1): 139–158.

Oliveira-Filho, A.T., Vilela, E.A., Carvalho, D.A. e Gavilanes, M.L. 1995. Estudos floristicos e fitossociológicos em remanescentes de matas ciliares do alto e médio rio Grande./Belo Horizonte, -27p.:il. UFLA, CEMIG.

Oliveira, R.J., Mantovani, W. e Melo, M.M.R.F. 2001. Estrutura do componente arbustivo-arbóreo da floresta Atlântica de encosta, Peruíbe, SP. Acta bot Bras 15:391-412.

Oliveira, R.R. 2002. Ação antrópica e resultantes sobre a estrutura e composição da Mata Atlântica na Ilha Grande, RJ. Rodriguésia 53:33-58.

Oliveira, Y.M.M., and Rotta, E. 1982. Levantamento da estrutura horizontal de uma mata de araucária do primeiro planalto paranaense. Boletim de Pesquisa Florestal 4:1-46.

Pereira, M.C.A., Araújo, D.S.D. e Pereira, O.J. 2001. Estrutura de uma comunidade arbustiva da restinga de Barra de Maricá – RJ. Rev. Bras. Bot. 24:273-281.

Pereira, O.J. e Assis, A.M. 2000. Florística da restinga de Camburi, Vitória, ES. Acta bot Bras 14:99-111.

Pereira, O.J. e Gomes, J.M.L. 1994. Levantamento florístico das comunidades vegetais de restinga no município de Conceição da Barra, ES. In: Anais do 3º Simpósio sobre Ecossistemas da Costa Brasileira 2:67-78.

Pereira, O.J. e Zambom, O. 1998. Composição florística da restinga de Interlagos, Vila Velha (ES). In: Anais do 4º Simpósio sobre Ecossistemas Brasileiros 3:129-139.

Pereira, O.J., Assis, A.M. e Souza, R.L.D. 1998. Vegetação da restinga de Pontal do Ipiranga, Município de Linhares (ES). In: Anais do 4º Simpósio sobre Ecossistemas Brasileiros 3:117-128.

Pereira, O.J., Borgo, J.H., Rodrigues, I.D. e Assis, A.M. 2000. Composição florística de uma floresta de restinga no município de Serra – ES. In: Anais do 5º Simpósio sobre Ecossistemas Brasileiros, 3:74-83.

Pessoa, S.V.A. e Oliveira, R.R. 2006. Análise estrutural da vegetação arbórea em três fragmentos florestas na Reserva Biológica de Poço das Antas, Rio de Janeiro, Brasil. Rodriguésia 57:391-411.

Pinheiro, M.H.O. e Monteiro, R. 2008. Florística de uma Floresta Estacional Semidecidual, localizada em ecótono savânico-florestal, no município de Bauru, SP, Brasil. Acta bot. bras. 22(4): 1085-1094.

Porto, M.L., Wildi, O., e Assunção, A.F. 2008. Análise de gradiente de comunidades vegetais e sua relação com fatores edáficos em um remanescente florestal no sul do Brasil. Em Porto, M.L. e colaboradores. Comunidades vegetais e fitossociologia: fundamentos para avaliação e manejo de ecossistemas. Porto Alegre : Ed. UFRGS, p. 162-183.

Reis-Duarte, R.M. 2004. Estrutura da floresta de restinga do Parque Estadual da Ilha Anchieta (SP): bases para promover o enriquecimento com espécies arbóreas nativas em solos alterados. PhD Thesis, Instituro de Biociências da Universidade Estadual Paulista Júlio de Mesquita Filho.

Ribas, R.F., Neto, J.A.A.M., Silva, A.F. e Agostinho Lopes de Souza, A.L. 2003. Composição florística de dois trechos em diferentes etapas serais de uma floresta estacional semidecidual em Viçosa, Minas Gerais. R. Árvore, Viçosa-MG, v.27, n.6, p.821-830.

Ríos, R.C., Galvão, F., and Curcio, G.R. 2008. Variaciones estruturales de la vegetacion arborea em tres ambientes de uma selva con Araucaria em Misiones, Argentina. Floresta 38:743-756.

### **Rodrigues, L.A. e Araújo, G.M. 1997. Levantamento florístico de uma mata decídua em Uberlândia, Minas Gerais, Brasil** Acta Bot. Bras. vol.11 no.2 Feira de Santana Dec.

Rodrigues, L.A., Carvalho, D.A., Oliveira-Filho, A.T., Botrel, R.T. e Silva, E.A. 2003. Florística e estrutura da comunidade arbórea de um fragmento florestal em Luminárias, MG. Acta bot. bras. 17(1): 71-87.

Rolim, S.G., Ivanauskas, N.M., Rodrigues, R.R., Nascimento, M.T., Gomes, J.M.L., Folli, D.A. e Couto, H.T.Z. 2006. Composição Florística do estrato arbóreo da Floresta Estacional Semidecidual na Planície Aluvial do rio Doce, Linhares, ES, Brasil. Acta bot. bras. 20(3): 549-561.

Rondon-Neto, R.M., Watzlawick, L.F., Caldeira, M.V.W., and Schoeninger, E.R. 2002. Análise florística e estrutural de um fragmento de floresta ombrófila mista Montana, situado em Criúva, RS – Brasil. Ciência Florestal 12:29-37.

Rosa, S.F., Longhi, S.J., Ludwig, M.P. 2008. Aspectos florísticos e fitossociológicos da Reserva Capão de Tupanciretã, Tupanciretã, RS, Brasil. Ciência Florestal, Santa Maria, v. 18, n. 1, p. 15-25, jan.-mar.

Rosário, D. 2001. Padrões florísticos e tipos funcionais em floresta com Araucária e suas relações com o solo. Dissertation. Universidade Federal do Rio Grande do Sul, Porto Alegre, Rio Grande do Sul, Brazil.

Rossoni, M.G. e Baptista, L.R.M. 1995. Composição florística da mata de restinga, Balneário Rondinha Velha, Arroio do Sal, RS, Brasil. Pesquisas-Botânica 45:115-131.

Rotta, E., Boerger, M.R.T. e Grodzki, L. 1997. Levantamento florístico e fitossociológico de um trecho de Floresta Ombrófila Densa das Terras Baixas no Parque Estadual do Palmito, Paranaguá, PR. Braz Arch Biol Techn 40:849-861.

Ruschel, A.R., Guerra, M.P. e Nodari, R.O. 2009. Estrutura e composição florística de dois fragmentos da Floresta Estacional Decidual do Alto-Uruguai, SC. Ciência Florestal, Santa Maria, v. 19, n. 2, p. 225-236, abr.-jun.

Ruschel, A.R., Nodari, R.O., Moerschbacher, B.M. 2007. Woody plant species richness in the Turvo State park, a large remnant of deciduous Atlantic forest, Brazil. Biodivers Conserv (2007) 16:1699–1714

Sá, C.F.C. 1992. A vegetação da restinga de Ipirangas, Reserva Ecológica Estadual de Jacarepiá, Saquarema (RJ): fisionomia e listagem de Angiospermas. Arquivos do Jardim Botânico do Rio de Janeiro 31:87-102.

Salis, S.M., Silva, M.P., Mattos, P.P., Silva, J.S.V., Pott, V.J. e ARNILDO Pott, A. 2004. Fitossociologia de remanescentes de floresta estacional decidual em Corumbá, Estado do Mato Grosso do Sul, Brasil , Revista Brasil. Bot., V.27, n.4, p.671-684, out.-dez.

Sanquetta, C.R., Pizzatto, W., Péllico Netto, S., and Figueiredo- Filho, A. 2000. Dinâmica da composição florística de um fragmento de floresta ombrófila mista no centro-sul do Paraná. Revista de Ciências Exatas e Naturais 1:78-88.

Schaaf, L.B., Figueiredo-Filho, A., Sanquetta, C.R., and Galvão, F. 2005. Incremento diamétrico e em área basal no período 1979–2000 de espécies arbóreas de uma floresta ombrófila mista localizada no sul do Paraná. Floresta 35: 271–290.

Schorn, L.A. 2005. Estrutura e dinâmica de estágios sucessionais de uma floresta ombrófila densa em Blumenau, Santa Catarina. MSc Dissertation, Setor de Ciências Agrárias da Universidade Federal do Paraná.

Scipioni, M.C., Longhi, S.L., Araújo, M.J. e Dalvan José Reinert, D.J. 2009. Regeneração natural de um fragmento da Floresta Estacional Decidual na Reserva Biológica do Ibicuí- Mirim (RS). Floresta, Curitiba, PR, v. 39, n. 3, p. 675-690, jul./set.

Silva, A.F., Oliveira, R.V., Santos, N.R.L. e de Paula, A. 2003. Composição florística e grupos ecológicos das espécies de um trecho de Floresta Semidecídua Submontana da Fazenda São Geraldo, Viçosa-MG. Revista Árvore, Viçosa-MG, v.27, n.3, p.311-319.

Silva, C.A., Pereira-Noronha, M.R., Santos, J., Lima, F.P. e Stefani, E.J.F. Fitossociologia de um fragmento de floresta estacional semidecidual em Ilha Solteira, São Paulo. UNESP, Ilha Solteira SP.

Silva, C.R. 2006. Fitossociologia e avaliação da chuva de sementes em uma área de floresta alta de restinga, em Ilha Comprida - São Paulo. Msc Dissertation, Instituto de Botânica de São Paulo.

Silva, C.T., dos Reis, G.G., Reis, M.G.F., Silva, E., Chaves, R.A. 2004. Avaliação temporal da florística arbórea de uma Floresta Secundária no município de Viçosa, Minas Gerais. Revista Árvore, Viçosa-MG, v.28, n.3, p. 429-441.

Silva, F.C. 1994. Composição florística e estrutura fitossociológica da floresta tropical ombrófila da encosta Atlântica no município de Morretes, Estado do Paraná. Acta Biológica Paranaense 23:1-54.

Silva, F.C., and Marconi, L.P. 1990. Fitossociologia de uma floresta com Araucária em Colombo, PR. Boletim de Pesquisa Florestal 20:23-38.

Silva, G.C. e Nascimento, M.T. 2001. Fitossociologia de um remanescente de mata sobre tabuleiros no norte do estado do Rio de Janeiro (Mata do Carvão)1Revta brasil. Bot., São Paulo, V.24, n.1, p.51-62, mar.

Silva, J.A., Salomão, A.N., Gripp, A., and Leite, E.J. 1997. Phytosociological survey in Brazilian forest genetic reserve of Caçador. Plant Ecology 133:1-11.

### Silva, L.A. e Aldicir Scariot, A. 2004. Comunidade arbórea de uma Floresta Estacional Decídua sobre afloramento calcário na Bacia do rio Paraná. Revista Árvore vol.28 no.1 Viçosa Jan./Feb.

Silva, L.A., e Scariot, A. 2003. Composição florística e estrutura da comunidade arbórea em uma Floresta Estacional Decidual em afloramento calcário (Fazenda São José, São Domingos, GO, Bacia do rio Paraná). Acta bot. bras. 17(2): 305-313.

Silva, M.R. e Araújo, G.M.,2009. Dinâmica da comunidade arbórea de uma floresta semidecidual em Uberlândia, MG, Brasil , Acta bot. bras. 23(1): 49-56.

Silva, S.M. 1998. As formações vegetais da planície litorânea da Ilha do Mel, Paraná, Brasil: composição florística e principais características estruturais. PhD Thesis, Instituto de Biologia da Universidade Estadual de Campinas.

Silva, S.M., Britez, R.M., Souza, W.S. e Joly, C.A. 1994. Fitossociologia do componente arbóreo da floresta de restinga da Ilha do Mel, Paranaguá, PR. In: Anais do 3º Simpósio sobre Ecossistemas da Costa Brasileira 2:47-56.

Siminski, A., Mantovani, M., Reis, M.S. e Fantini, A.C. 2004. Sucessão florestal secundária no município de São Pedro de Alcântara, Litoral de Santa Catarina: estrutura e diversidade. Cienc Florest 14:21-33.

Siqueira, A.S., Araújo, G.M.A. e Schiavini, I. 2009. Estrutura do componente arbóreo e características edáficas de dois fragmentos de floresta estacional decidual no vale do rio Araguari, MG, Brasil. Acta bot. bras. 23(1): 10-21.

Sobrinho, F.A.P., Christo, A.G., Guedes-Bruni, R.R. e Silva, A.F. 2009. Composição florística e estrutura de um fragmento de Floresta Estacional Semidecidual Aluvial em Viçosa (MG). Floresta, Curitiba, PR, v. 39, n. 4, p. 793-805, out./dez.

Sonego, R.C., Backes, A., and Souza, A.F. 2007. Descrição da estrutura de uma Floresta Ombrófila Mista, RS, Brasil, utilizando estimadores não-paramétricos de riqueza e rarefação de amostras. Acta Botanica Brasilica 21:943-955.

Souza, C.A. 2001. Estrutura do componente arbóreo de floresta pluvial subtropical na Serra dos Tapes, sul do Rio Grande do Sul. Dissertação de Mestrado, Porto Alegre: UFRGS.

### Souza, J.S., Espírito-Santo, F.D.B., Fontes, M.A.L., Oliveira-Filho, A.T. e Botezelli, L. 2003. Análise das variações florísticas e estruturais da comunidade arbórea de um fragmento de Floresta Semidecídua às margens do rio Capivari, Lavras-MG. Revista Árvore vol.27 no.2 Viçosa Mar./Apr.

Sugiyama, M. 1998. Estudo de florestas de restinga da Ilha do Cardoso, Cananéia, São Paulo, Brasil. Boletim do Instituto de Botânica 11:119-159.

Tanaka G.K. 2009. Estrutura e florística do estrato arbóreo de um fragmento de floresta estacional semidecidual: Estação Ecológica de Ribeirão Preto, SP. Dissertação, USP.

Torezan, J.M.D. 1995. Estudo da sucessão secundária na Floresta Ombrófila Densa Submontana, em áreas anteriormente cultivadas pelo sistema de “coivara”, em Iporanga-SP. MSc Dissertation, Setor de Ciências Biológicas da Universidade Federal do Paraná.

Vaccaro, S. 1991. Caracterização fitossociológica de três fases sucessionais de uma Floresta Estacional Decidual, no município de Santa Tereza – RS. Dissertação UFSM.

Vaccaro, S. 1997. Caracterização fitossociológica de três fases sucessionais de uma floresta estacional decidual, no município de Santa Tereza - RS. Dissertação de Mestrado, Santa Maria: UFSM.

Vaccaro, S. Longhi, S.J. 1995. Análise Fitossociologica de Algumas Áreas Remanescentes da Floresta do Alto Uruguai, Entre os Rios Ijui e Turvo, no Rio Grande do Sul. Ciência. Florestal., Santa Maria, v.5, n.1, p. 33-53.

### **Van Den Berg, E. e Oliveira-Filho, A.T. 2000. Composição florística e estrutura fitossociológica de uma floresta ripária em Itutinga, MG, e comparação com outras áreas** Rev. bras. Bot. vol.23 no.3 São Paulo Sept.

Vargas, D. 2005. Floristica, fitossociologia e aspectos da dinamica de um remanescente de mata de enconsta no morro Santana, Porto Alegre, Rio Grande do Sul. Universidade Federal do Rio Grande do Sul, PPG Ecologia, Dissertação de mestrado. Poto Alegre.

Waechter, J.L., Muller, S.C., Breier, T.B. e Venturi, S. 2000. Estrutura do componente arbóreo em uma floresta subtropical de planície costeira interna. In: Anais do 5º Simpósio sobre Ecossistemas Brasileiros 3:92-112.

Yamamoto, L.F., Kinoshita, L.S. e Martins, F.R. 2007. Síndromes de polinização e de dispersão em fragmentos da Floresta Estacional Semidecídua Montana, SP, Brasil , Acta bot. bras. 21(3): 553-573.
