# Supplementary material for: Phylobetadiversity among Forest Types in the Brazilian Atlantic Forest Complex
Source: PLoS One. 2014 Aug 14;9(8):e105043. doi: 10.1371/journal.pone.0105043 (PMC4133375; doi:10.1371/journal.pone.0105043)
Supplement: Appendix S2 — Time-calibrated phylogenetic tree used to carry out phylogenetic structure and phylobetadiversity analyses. (DOCX) [file pone.0105043.s002.docx]

**Appendix S2**: Time-calibrated phylogenetic tree used to carry out phylogenetic structure and phylobetadiversity analyses (1,916 species). The tree was built based on the megatree R20120829 (available at <https://github.com/camwebb/tree-of-trees/blob/master/megatrees/R20120829.new>). References of constructed clades inserted into the megatree and references of clades age estimates combined with Bell *et al*. (2010) age estimates.

**((((((((((((((((((justicia_brasiliana:41.0,ruellia_brevifolia:41.0)acanthaceae:15.5,((((cybistax_antisyphilitica:14.143517,(zeyheria_montana:7.071758,zeyheria_tuberculosa:7.071796)zeyheria:7.071758):14.143517,(handroanthus_albus:8.486111,handroanthus_botelhensis:8.486149,handroanthus_catarinensis:8.486149,handroanthus_chrysotrichus:8.486149,handroanthus_cristatus:8.486149,handroanthus_heptaphyllus:8.486149,handroanthus_ochraceus:8.486149,handroanthus_pulcherrimus:8.486149,handroanthus_serratifolius:8.486149,handroanthus_umbellatus:8.486149,handroanthus_vellosoi:8.486149)handroanthus:19.800924,(tabebuia_aurea:7.071758,tabebuia_obtusifolia:7.071796,tabebuia_roseoalba:7.071796,tabebuia_stenocalyx:7.071796)tabebuia:21.215275):5.657408,sparattosperma_leucanthum:33.944443,paratecoma_peroba:33.944489)tabebuiaalliance:21.438889,(jacaranda_bracteata:27.691666,jacaranda_brasiliana:27.691696,jacaranda_cuspidifolia:27.691696,jacaranda_jasminoides:27.691696,jacaranda_macrantha:27.691696,jacaranda_micrantha:27.691696,jacaranda_puberula:27.691696,jacaranda_subalpina:27.691696)jacaranda:27.691666)bignoniaceae:1.116669):1.116669,(((aloysia_virgata:12.500001,((lantana_camara:2.083333,lantana_canescens:2.083384,lantana_fucata:2.083384,lantana_hypoleuca:2.083384,lantana_trifolia:2.083384)lantana:2.083333,lippia_alba:4.166666):8.333333):13.750000,(citharexylum_montevidense:13.125000,(citharexylum_myrianthum:6.562500,citharexylum_solanaceum:6.562531)citharexylum:6.562500):13.125000):1.875000,((duranta_erecta:5.273438,duranta_vestita:5.273468)duranta:5.273438,recordia_reitzii:10.546875):17.578125):29.491669):1.116665,((aegiphila_brachiata:20.0,aegiphila_fluminensis:20.0,aegiphila_integrifolia:20.0,aegiphila_mediterranea:20.0,aegiphila_obducta:20.0,aegiphila_verticillata:20.0)aegiphila:20.0,(hyptidendron_arboreum:20.0,hyptidendron_asperrimum:20.0,hyptidendron_canum:20.0)hyptidendron:20.0,(vitex_cymosa:20.0,vitex_megapotamica:20.0,vitex_polygama:20.0,vitex_rufescens:20.0,vitex_sellowiana:20.0)vitex:20.0)lamiaceae:18.733398):11.666668,((chionanthus_filiformis:22.5,chionanthus_micranthus:22.5,chionanthus_trichotomus:22.5)chionanthus:22.5,priogymnanthus_hasslerianus:45.0)oleaceae:25.400066):14.933336,((((((((acnistus_arborescens:2.638889,vassobia_breviflora:2.638889):10.027779,(aureliana_fasciculata:2.375000,aureliana_velutina:2.375031)aureliana:10.291667):4.222223,capsicum_flexuosum:16.888889):2.111111,(solanum_acerifolium:15.200043,solanum_americanum:15.200043,solanum_aparadense:15.200043,solanum_argenteum:15.200043,solanum_atropurpureum:15.200043,solanum_aturense:15.200043,solanum_bonariense:15.200043,solanum_bullatum:15.200043,solanum_caavurana:15.200043,solanum_cernuum:15.200043,solanum_cinnamomeum:15.200043,solanum_cladotrichum:15.200043,solanum_compressum:15.200043,solanum_corymbiflorum:15.200043,solanum_diploconos:15.200043,solanum_enantiophyllanthum:15.200043,solanum_granulosoleprosum:15.200043,solanum_johannae:15.200043,solanum_lacerdae:15.200043,solanum_lepidotum:15.200043,solanum_leucodendron:15.200043,solanum_lycocarpum:15.200043,solanum_martii:15.200043,solanum_paniculatum:15.200043,solanum_paranense:15.200043,solanum_pseudocapsicum:15.200043,solanum_pseudoquina:15.200043,solanum_ramulosum:15.200043,solanum_sanctaecatharinae:15.200043,solanum_scuticum:15.200043,solanum_sessile:15.200043,solanum_swartzianum:15.200043,solanum_sycocarpum:15.200043,solanum_variabile:15.200043,solanum_viarum:15.200043)solanum:3.800001):1.900000,brugmansia_suaveolens:20.900000):3.800002,dyssochroma_viridiflora:24.700003):9.500002,(brunfelsia_australis:12.214286,brunfelsia_brasiliensis:12.214334,brunfelsia_cuneifolia:12.214334,brunfelsia_pilosa:12.214334,brunfelsia_uniflora:12.214334)brunfelsia:21.985714,((cestrum_axillare:2.442858,cestrum_bracteatum:2.442885,cestrum_corymbosum:2.442885,cestrum_gardneri:2.442885,cestrum_intermedium:2.442885,cestrum_mariquitense:2.442885,cestrum_pedicellatum:2.442885,cestrum_schlechtendalii:2.442885,cestrum_strigilatum:2.442885)cestrum:2.442858,(sessea_brasiliensis:2.442858,sessea_regnellii:2.442885)sessea:2.442858):29.314287):1.899998,metternichia_princeps:36.099998):49.233337):9.333328,(((((aspidosperma_australe:9.000000,aspidosperma_cuspa:9.000031,aspidosperma_cylindrocarpon:9.000031,aspidosperma_discolor:9.000031,aspidosperma_eburneum:9.000031,aspidosperma_illustre:9.000031,aspidosperma_macrocarpon:9.000031,aspidosperma_multiflorum:9.000031,aspidosperma_olivaceum:9.000031,aspidosperma_parvifolium:9.000031,aspidosperma_polyneuron:9.000031,aspidosperma_pyricollum:9.000031,aspidosperma_ramiflorum:9.000031,aspidosperma_spruceanum:9.000031,aspidosperma_subincanum:9.000031,aspidosperma_tomentosum:9.000031)aspidosperma:9.000000,geissospermum_laeve:18.000000):36.000000,(((hancornia_speciosa:40.444443,(macoubea_guianensis:34.666668,(tabernaemontana_catharinensis:9.244445,tabernaemontana_hystrix:9.244477,tabernaemontana_laeta:9.244477)tabernaemontana:25.422222)tabernaemontaneae:5.777775):5.777779,(rauvolfia_grandiflora:5.777778,rauvolfia_mattfeldiana:5.777825,rauvolfia_sellowii:5.777825)rauvolfia:40.444443):5.777779,((himatanthus_lancifolius:6.125000,himatanthus_obovatus:6.125031,himatanthus_phagedaenicus:6.125031)himatanthus:42.875000,malouetia_cestroides:49.000000):3.000000):2.000000)apocynaceae:7.500000,(strychnos_brasiliensis:20.500000,strychnos_parvifolia:20.500061)strychnos:41.000000):7.500000,((((((((calycophyllum_multiflorum:14.768182,(alseis_floribunda:7.384091,alseis_involuta:7.384128,alseis_pickelii:7.384128)alseis:7.384091):4.922727,(simira_corumbensis:4.922727,simira_glaziovii:4.922762,simira_sampaioana:4.922762,simira_viridiflora:4.922762)simira:14.768181):9.845456,((bathysa_australis:3.692045,bathysa_gymnocarpa:3.692079,bathysa_mendoncaei:3.692079,bathysa_nicholsonii:3.692079,bathysa_stipulata:3.692079)bathysa:3.692045,warszewiczia_longistaminea:7.384091):22.152271)septicidalclade:4.922728,((rustia_formosa:9.189091,rustia_gracilis:9.189144)rustia:18.378181,schizocalyx_cuspidatus:27.567272)loculicidalclade:6.891819):14.768181,(((alibertia_edulis:10.795454,(amaioua_guianensis:5.397727,amaioua_intermedia:5.397774)amaioua:5.397727,(cordiera_concolor:5.397727,cordiera_elliptica:5.397774,cordiera_macrophylla:5.397774,cordiera_myrciifolia:5.397774,cordiera_sessilis:5.397774)cordiera:5.397727,melanopsidium_nigrum:10.795455)alibertiagroup:5.397727,((genipa_americana:3.238636,genipa_infundibuliformis:3.238636):6.477272,(randia_ferox:6.477273,(tocoyena_bullata:3.238636,tocoyena_formosa:3.238658,tocoyena_sellowiana:3.238658)tocoyena:3.238636):3.238636):6.477273):22.670454,(ixora_brevifolia:5.829545,ixora_gardneriana:5.829573,ixora_venulosa:5.829573)ixora:33.034088):10.363636):2.590908,(posoqueria_acutifolia:7.402597,posoqueria_latifolia:7.402639)posoqueria:44.415581)ixoroideae:2.590912,(((((chomelia_bella:7.949380,chomelia_brasiliana:7.949424,chomelia_nitidula:7.949424,chomelia_obtusa:7.949424,chomelia_parvifolia:7.949424,chomelia_ribesioides:7.949424,chomelia_sericea:7.949424,chomelia_sessilis:7.949424)chomelia:13.248966,(guettarda_pohliana:7.066116,guettarda_sericea:7.066147,guettarda_uruguensis:7.066147,guettarda_viburnoides:7.066147)guettarda:14.132231):12.719007,machaonia_brasiliensis:33.917355):12.719009,((coutarea_hexandra:31.090910,salzmannia_nitida:31.090908):11.659090,(hamelia_patens:32.062500,(hillia_ilustris:5.343750,hillia_parasitica:5.343781)hillia:26.718750):10.687500):3.886364):3.886364,ladenbergia_hexandra:50.522728)cinchonoideae:3.886364):2.590909,(((coussarea_capitata:9.104167,coussarea_contracta:9.104207,coussarea_graciliflora:9.104207,coussarea_hydrangeifolia:9.104207,coussarea_meridionalis:9.104207,coussarea_nodosa:9.104207,coussarea_platyphylla:9.104207)coussarea:9.104167,(faramea_hyacinthina:9.104167,faramea_latifolia:9.104207,faramea_montevidensis:9.104207,faramea_multiflora:9.104207,faramea_pachyantha:9.104207,faramea_porophylla:9.104207,faramea_truncata:9.104207)faramea:9.104167):30.347223,(((margaritopsis_astrellantha:31.439316,(rudgea_coriacea:7.859829,rudgea_gardenioides:7.859867,rudgea_insignis:7.859867,rudgea_jasminoides:7.859867,rudgea_parquioides:7.859867,rudgea_recurva:7.859867,rudgea_reticulata:7.859867,rudgea_sessilis:7.859867,rudgea_triflora:7.859867,rudgea_umbrosa:7.859867,rudgea_vellerea:7.859867,rudgea_viburnoides:7.859867)rudgea:23.579487):2.858120,(palicourea_guianensis:8.574359,palicourea_marcgravii:8.574384,palicourea_rigida:8.574384)palicourea:25.723076)paulicoreeae:2.858120,(psychotria_bahiensis:16.255554,psychotria_brachyceras:16.255585,psychotria_capitata:16.255585,psychotria_carthagenensis:16.255585,psychotria_deflexa:16.255585,psychotria_forsteronioides:16.255585,psychotria_glaziovii:16.255585,psychotria_hastisepala:16.255585,psychotria_hoffmannseggiana:16.255585,psychotria_laciniata:16.255585,psychotria_leiocarpa:16.255585,psychotria_mapourioides:16.255585,psychotria_myriantha:16.255585,psychotria_nemorosa:16.255585,psychotria_nuda:16.255585,psychotria_patentinervia:16.255585,psychotria_rhytidocarpa:16.255585,psychotria_stachyoides:16.255585,psychotria_subspathacea:16.255585,psychotria_suterella:16.255585,psychotria_tenerior:16.255585,psychotria_vellosiana:16.255585)psychotriaclade123:20.900002):11.400003):8.444444)rubiaceae:12.000000)gentianales:25.666664,(((cordia_alliodora:3.325000,cordia_americana:3.325024,cordia_brasiliensis:3.325024,cordia_ecalyculata:3.325024,cordia_glabrata:3.325024,cordia_magnoliifolia:3.325024,cordia_rufescens:3.325024,cordia_sellowiana:3.325024,cordia_sericicalyx:3.325024,cordia_silvestris:3.325024,cordia_superba:3.325024,cordia_taguahyensis:3.325024)cordia:9.975000,cordia_trichotoma:13.300000):6.650001,(varronia_curassavica:9.975000,varronia_guazumaefolia:9.975042,varronia_polycephala:9.975042)varronia:9.975000):74.716660):9.333333,(emmotum_nitens:69.333336,leretia_cordata:69.333359)icacinaceae:34.666668)lamiidae:4.000000,(((sambucus_australis:76.000063,(aralia_warmingiana:15.545454,((dendropanax_affinis:3.454545,dendropanax_arboreus:3.454573,dendropanax_cuneatus:3.454573)dendropanax:10.363636,(schefflera_angustissima:6.045455,schefflera_calva:6.045488,schefflera_longepetiolata:6.045488,schefflera_macrocarpa:6.045488,schefflera_morototoni:6.045488,schefflera_selloi:6.045488,schefflera_succinea:6.045488,schefflera_vinosa:6.045488)neotropicalschefflera:7.772727,(oreopanax_capitatus:2.590909,oreopanax_fulvum:2.590945)oreopanax:11.227273):1.727273):60.454544):16.000000,((((((baccharis_dentata:1.954545,baccharis_genistelloides:1.954587,baccharis_lateralis:1.954587,baccharis_linearifolia:1.954587,baccharis_lymanii:1.954587,baccharis_nummularia:1.954587,baccharis_oblongifolia:1.954587,baccharis_pingraea:1.954587,baccharis_punctulata:1.954587,baccharis_semiserrata:1.954587,baccharis_uncinella:1.954587)baccharis:21.500000,((austrocritonia_angulicaulis:0.651515,austrocritonia_velutina:0.651560)austrocritonia:0.651515,(austroeupatorium_inulaefolium:0.651537,austroeupatorium_picturatum:0.651537)austroeupatorium:0.651537,barrosoa_betonicaeformis:1.303075,campovassouria_cruciata:1.303075,chromolaena_picta:1.303075,grazielia_serrata:1.303075,hatschbachiella_tweedieana:1.303075,heterocondylus_alatus:1.303075,kaunia_rufescens:1.303075,(symphyopappus_compressus:0.651537,symphyopappus_itatiayensis:0.651537,symphyopappus_lymansmithii:0.651537)symphyopappus:0.651537,verbesina_claussenii:1.303075)eupatorieae:22.151514,dendrophorbium_glaziovii:23.454546)asteroideae:3.909090,((eremanthus_erythropappus:1.303030,eremanthus_glomerulatus:1.303058,eremanthus_incanus:1.303058,eremanthus_polycephalus:1.303058)eremanthus:1.303030,(lepidaploa_canescens:1.303044,lepidaploa_pseudomuricata:1.303044)lepidaploa:1.303044,lessingianthus_carduoides:2.606088,(piptocarpha_angustifolia:1.303044,piptocarpha_axillaris:1.303044,piptocarpha_macropoda:1.303044,piptocarpha_notata:1.303044,piptocarpha_organensis:1.303044,piptocarpha_regnellii:1.303044,piptocarpha_rotundifolia:1.303044,piptocarpha_sellowii:1.303044)piptocarpha:1.303044,(vernonanthura_brasiliana:1.303044,vernonanthura_catharinensis:1.303044,vernonanthura_discolor:1.303044,vernonanthura_divaricata:1.303044,vernonanthura_ferruginea:1.303044,vernonanthura_montevidensis:1.303044,vernonanthura_petiolaris:1.303044,vernonanthura_phosphorica:1.303044,vernonanthura_puberula:1.303044,vernonanthura_tweediana:1.303044)vernonanthura:1.303044,vernonia_rubriramea:2.606088)vernonieae:24.757574):9.772728,(moquiniastrum_barrosoae:12.378788,moquiniastrum_paniculatum:12.378838,moquiniastrum_polymorphum:12.378838)moquiniastrum:24.757576):3.909092,jungia_sellowii:41.045456,stifftia_chrysantha:41.045456):1.954545,(dasyphyllum_brasiliense:14.333333,dasyphyllum_spinescens:14.333396,dasyphyllum_tomentosum:14.333396)dasyphyllum:28.666666)asteraceae:49.000000,(escallonia_bifida:36.0,escallonia_chlorophylla:36.0,escallonia_megapotamica:36.0)escallonia:56.000063):8.000000,((ilex_affinis:22.500061,ilex_brasiliensis:22.500061,ilex_brevicuspis:22.500061,ilex_cerasifolia:22.500061,ilex_chamaedryfolia:22.500061,ilex_conocarpa:22.500061,ilex_dumosa:22.500061,ilex_integerrima:22.500061,ilex_microdonta:22.500061,ilex_paraguariensis:22.500061,ilex_pseudobuxus:22.500061,ilex_sapotifolia:22.500061,ilex_taubertiana:22.500061,ilex_theezans:22.500061)ilex:64.500000,(citronella_gongonha:24.333334,citronella_paniculata:24.333361)citronella:62.666668)aquifoliales:13.000000)campanulidae:8.000000)lamiidae_and_campanulidae:2.000000,(((((clethra_scabra:10.908334,clethra_uleana:10.908378)clethra:32.725002,((agarista_eucalyptoides:7.5,agarista_glaberrima:7.5,agarista_oleifolia:7.5)agarista:7.5,(gaultheria_eriophylla:7.5,gaultheria_itatiaiae:7.5)gaultheria:7.5,gaylussacia_brasiliensis:15.0)ericaceae:28.633403):28.633335,((styrax_acuminatus:19.0,styrax_aureus:19.0,styrax_camporum:19.0,styrax_ferrugineus:19.0,styrax_glabratus:19.0,styrax_latifolius:19.0,styrax_leprosus:19.0,styrax_pohlii:19.0)styrax:44.133403,(symplocos_arbutifolia:15.783378,symplocos_celastrinea:15.783378,symplocos_estrellensis:15.783378,symplocos_falcata:15.783378,symplocos_glandolosomarginata:15.783378,symplocos_insignis:15.783378,symplocos_itatiaiae:15.783378,symplocos_laxiflora:15.783378,symplocos_nitidiflora:15.783378,symplocos_oblongifolia:15.783378,symplocos_paraensis:15.783378,symplocos_pubescens:15.783378,symplocos_tenuifolia:15.783378,symplocos_tetrandra:15.783378,symplocos_trachycarpos:15.783378,symplocos_uniflora:15.783378)symplocos:47.350002):9.133335,(laplacea_acutifolia:24.088890,laplacea_fruticosa:24.088943)laplacea:48.177780):9.133331,(((diospyros_apeibacarpos:23.600000,diospyros_brasiliensis:23.600050,diospyros_hispida:23.600050,diospyros_inconstans:23.600050)diospyros:47.200001,((((ardisia_guianensis:18.350000,(geissanthus_ambiguus:6.116667,(stylogyne_lhotzkyana:3.058333,stylogyne_martiana:3.058366,stylogyne_pauciflora:3.058366)stylogyne:3.058333):12.233334):3.750000,(myrsine_balansae:3.683333,myrsine_coriacea:3.683366,myrsine_gardneriana:3.683366,myrsine_guianensis:3.683366,myrsine_hermogenesii:3.683366,myrsine_laetevirens:3.683366,myrsine_lancifolia:3.683366,myrsine_lineata:3.683366,myrsine_loefgrenii:3.683366,myrsine_parvifolia:3.683366,myrsine_parvula:3.683366,myrsine_umbellata:3.683366,myrsine_venosa:3.683366,myrsine_villosissima:3.683366)myrsine:18.416668):19.050001,(cybianthus_brasiliensis:20.575027,cybianthus_cuneifolius:20.575027,cybianthus_indecorus:20.575027)cybianthus:20.575027)myrsinoideae:17.300001,((clavija_nutans:8.566667,clavija_spinosa:8.566695)clavija:25.700001,jacquinia_armillaris:34.266666):24.183332):12.350002):5.299995,(((chrysophyllum_flexuosum:11.191176,chrysophyllum_gonocarpum:11.191209,chrysophyllum_inornatum:11.191209,chrysophyllum_januariense:11.191209,chrysophyllum_lucentifolium:11.191209,chrysophyllum_marginatum:11.191209,chrysophyllum_splendens:11.191209,chrysophyllum_viride:11.191209)chrysophyllum:33.573528,((((diploon_cuspidatum:8.952941,(pradosia_kuhlmannii:4.476470,pradosia_lactescens:4.476502)pradosia:4.476470,sarcaulus_brasiliensis:8.952941):8.952941,pouteria_gardneriana:17.905880):17.905882,ecclinusa_ramiflora:35.811760,(micropholis_crassipedicellata:17.905882,micropholis_gardneriana:17.905947,micropholis_guyanensis:17.905947,micropholis_venulosa:17.905947)micropholis:17.905882):4.476471,(pouteria_bangii:10.072059,pouteria_beaurepairei:10.072106,pouteria_bullata:10.072106,pouteria_caimito:10.072106,pouteria_coelomatica:10.072106,pouteria_durlandii:10.072106,pouteria_filipes:10.072106,pouteria_gardneri:10.072106,pouteria_glomerata:10.072106,pouteria_grandiflora:10.072106,pouteria_guianensis:10.072106,pouteria_hispida:10.072106,pouteria_peduncularis:10.072106,pouteria_psammophila:10.072106,pouteria_ramiflora:10.072106,pouteria_reticulata:10.072106,pouteria_salicifolia:10.072106,pouteria_torta:10.072106,pouteria_venosa:10.072106)pouteria:30.216175):4.476471):17.905880,(((manilkara_longifolia:11.937255,manilkara_salzmannii:11.937287,manilkara_subsericea:11.937287)manilkara:27.853594,mimusops_coriacea:39.790848):15.916340,sideroxylon_obtusifolium:55.707188)sapotoideae:6.963398):13.429413):5.300003,(ternstroemia_alnifolia:27.0,ternstroemia_brasiliensis:27.0)ternstroemia:54.400069):5.299995,((cariniana_domestica:10.666667,cariniana_estrellensis:10.666707,cariniana_legalis:10.666707)cariniana:10.666667,(couratari_macrosperma:10.666667,couratari_pyramidata:10.666707)couratari:10.666667,((eschweilera_compressa:8.000000,eschweilera_ovata:8.000031)eschweilera:8.000000,(lecythis_lanceolata:8.000000,lecythis_lurida:8.000031,lecythis_pisonis:8.000031)lecythis:8.000000):5.333334):65.366669):23.299999)ericales_and_others:6.000000,((achatocarpus_praecox:61.333336,((brasiliopuntia_brasiliensis:5.217593,(cereus_bicolor:2.608810,cereus_fernambucensis:2.608810,cereus_hildmannianus:2.608810)cereus:2.608810,(opuntia_ficusindica:2.608810,opuntia_monacantha:2.608810)opuntia:2.608810,pilosocereus_arrabidae:5.217620)cactaceae:41.740738,(((andradaea_floribunda:11.500000,ramisia_brasiliensis:11.500000):11.500000,(bougainvillea_glabra:15.164836,(((guapira_areolata:2.527472,guapira_cafferiana:2.527520,guapira_graciliflora:2.527520,guapira_hirsuta:2.527520,guapira_hoehnei:2.527520,guapira_laxiflora:2.527520,guapira_nitida:2.527520,guapira_noxia:2.527520,guapira_obtusata:2.527520,guapira_opposita:2.527520,guapira_pernambucensis:2.527520,guapira_salicifolia:2.527520,guapira_tomentosa:2.527520,guapira_venosa:2.527520)guapira:2.527472,neea_hermaphrodita:5.054946,neea_macrophylla:5.054945):5.054945,(pisonia_aculeata:5.054945,pisonia_ambigua:5.054979,pisonia_zapallo:5.054979)pisonia:5.054945):5.054944):7.835165)nyctaginaceae:4.791666,(gallesia_integrifolia:18.527777,(phytolacca_dioica:9.263899,phytolacca_thyrsiflora:9.263899)phytolacca:9.263899,(seguieria_americana:9.263899,seguieria_langsdorffii:9.263899)seguieria:9.263899,trichostigma_octandrum:18.527798)phytolaccaceae:9.263888):19.166666):14.375000):44.666672,((coccoloba_alnifolia:21.0,coccoloba_arborescens:21.0,coccoloba_cordifolia:21.0,coccoloba_cujabensis:21.0,coccoloba_declinata:21.0,coccoloba_mollis:21.0,coccoloba_warmingii:21.0)coccoloba:21.0,(ruprechtia_laxiflora:21.0,ruprechtia_lundii:21.0)ruprechtia:21.0,(triplaris_americana:21.0,triplaris_gardneriana:21.0)triplaris:21.0)polygonaceae:64.000063)caryophyllales:10.000000):2.000000,(((cathedra_bahiensis:33.000000,cathedra_rubricaulis:33.000061)cathedra:33.000000,(dulacia_papillosa:33.000031,dulacia_singularis:33.000031)dulacia:33.000031,(heisteria_ovata:33.000031,heisteria_perianthomega:33.000031,heisteria_silvianii:33.000031)heisteria:33.000031,tetrastylidium_grandifolium:66.000061,ximenia_americana:66.000061)olacaceae:33.000000,(((agonandra_brasiliensis:29.666666,agonandra_excelsa:29.666700)agonandra:59.333332,jodina_rhombifolia:89.000063)bell0563:5.000000,schoepfia_brasiliensis:94.000000):5.000000)santalales:19.000000):3.000000,((((((carpotroche_brasiliensis:85.666664,(((lacistema_hasslerianum:24.333334,lacistema_lucidum:24.333391,lacistema_pubescens:24.333391)lacistema:48.666668,(abatia_americana:63.0,azara_uruguayensis:63.0,(banara_parviflora:31.5,banara_tomentosa:31.5)banara:31.5,(casearia_aculeata:31.5,casearia_arborea:31.5,casearia_catharinensis:31.5,casearia_commersoniana:31.5,casearia_decandra:31.5,casearia_gossypiosperma:31.5,casearia_grandiflora:31.5,casearia_lasyophylla:31.5,casearia_mariquitensis:31.5,casearia_melliodora:31.5,casearia_obliqua:31.5,casearia_pauciflora:31.5,casearia_rufescens:31.5,casearia_rupestris:31.5,casearia_sylvestris:31.5,casearia_ulmifolia:31.5)casearia:31.5,prockia_crucis:63.0,salix_humboldtiana:63.0,(xylosma_ciliatifolia:31.5,xylosma_glaberrimum:31.5,xylosma_prockia:31.5,xylosma_pseudosalzmannii:31.5,xylosma_tweediana:31.5,xylosma_venosa:31.5)xylosma:31.5)salicaceae:10.000066)bell0859:6.333336,((amphirrhox_longifolia:43.692307,hybanthus_artropurpureus:43.692303):16.384617,(rinorea_guianensis:30.038462,rinorea_laevigata:30.038504)rinorea:30.038462):19.256409):6.333328):6.333333,(((calophyllum_brasiliense:41.904762,(kielmeyera_albopunctata:8.380953,kielmeyera_coriacea:8.380980,kielmeyera_decipiens:8.380980,kielmeyera_lathrophyton:8.380980,kielmeyera_membranacea:8.380980,kielmeyera_petiolaris:8.380980)kielmeyera:33.523811)calophylleae:16.761906,(vismia_baccifera:7.000000,vismia_brasiliensis:7.000031,vismia_guianensis:7.000031,vismia_latifolia:7.000031,vismia_martiana:7.000031,vismia_parviflora:7.000031)vismia:51.666668):16.666668,((((clusia_criuva:4.882716,clusia_fluminensis:4.882756,clusia_fragrans:4.882756,clusia_hilariana:4.882756,clusia_nemorosa:4.882756,clusia_spiritusanctensis:4.882756)clusia:24.413582,(tovomita_fructipendula:9.765432,tovomita_glazioviana:9.765451)tovomita:19.530865):9.765432,(tovomitopsis_paniculata:19.530865,tovomitopsis_saldanhae:19.530903)tovomitopsis:19.530865):19.530865,((garcinia_brasiliensis:25.111113,garcinia_gardneriana:25.111147,garcinia_macrophylla:25.111147)garcinia:25.111113,symphonia_globulifera:50.222225):8.370369)clusiaceae:16.740742):16.666666,caryocar_brasiliense:92.000000,((((couepia_belemii:1.416667,couepia_leitaofilhoi:1.416708,couepia_meridionalis:1.416708,couepia_schottii:1.416708,couepia_venosa:1.416708)couepia:4.250000,((hirtella_angustifolia:1.416667,hirtella_glandulosa:1.416708,hirtella_gracilipes:1.416708,hirtella_hebeclada:1.416708)hirtella:1.416667,(licania_apetala:1.416667,licania_arianeae:1.416708,licania_gardneri:1.416708,licania_hoehnei:1.416708,licania_humilis:1.416708,licania_hypoleuca:1.416708,licania_kunthiana:1.416708,licania_octandra:1.416708,licania_spicata:1.416708,licania_tomentosa:1.416708)licania:1.416667):2.833333):5.666666,(parinari_brasiliensis:2.125000,parinari_excelsa:2.125031)parinari:9.208333):52.666668,((stephanopodium_blanchetianum:16.666666,stephanopodium_engleri:16.666731,stephanopodium_estrellense:16.666731,stephanopodium_organense:16.666731)stephanopodium:33.333332,trigoniodendron_spiritusanctense:50.000000)bell0834:14.000000):28.000000,(erythroxylum_ambiguum:21.000000,erythroxylum_argentinum:21.000061,erythroxylum_buxus:21.000061,erythroxylum_citrifolium:21.000061,erythroxylum_cuneifolium:21.000061,erythroxylum_cuspidifolium:21.000061,erythroxylum_daphnites:21.000061,erythroxylum_deciduum:21.000061,erythroxylum_frangulifolium:21.000061,erythroxylum_myrsinites:21.000061,erythroxylum_oxypetalum:21.000061,erythroxylum_passerinum:21.000061,erythroxylum_pelleterianum:21.000061,erythroxylum_pulchrum:21.000061,erythroxylum_suberosum:21.000061,erythroxylum_subracemosum:21.000061,erythroxylum_subrotundum:21.000061,erythroxylum_subsessile:21.000061,erythroxylum_tenue:21.000061,erythroxylum_tortuosum:21.000061,erythroxylum_vaccinifolium:21.000061)erythroxylum:71.000000,(((((acalypha_brasiliensis:7.097142,acalypha_gracilis:7.097194,acalypha_villosa:7.097194)acalypha:48.102856,((adelia_membranifolia:18.400000,philyra_brasiliensis:18.400000):18.400000,bernardia_pulchella:36.799999):18.399998):12.266670,((alchornea_glandulosa:8.433333,alchornea_sidifolia:8.433366,alchornea_triplinervia:8.433366)alchornea:16.866665,aparisthmium_cordatum:25.299999):42.166668)acalyphoideaess:6.133331,(((((((actinostemon_concolor:9.200000,actinostemon_klotzschii:9.200025,actinostemon_schomburgkii:9.200025,actinostemon_verticillatus:9.200025)actinostemon:9.200000,senefeldera_verticillata:18.400000):18.400000,((mabea_fistulifera:4.600000,mabea_piriri:4.600043)mabea:13.799999,microstachys_corniculata:18.400000):18.399998):4.600002,maprounea_guianensis:41.400002)h1:4.599998,((((algernonia_leandrii:11.828571,algernonia_obovata:11.828594,algernonia_riedelii:11.828594)algernonia:11.828571,pachystroma_longifolium:23.657143):7.885714,pleradenophora_membranifolia:31.542856):7.885714,(((sapium_glandulosum:6.160714,sapium_haematospermum:6.160758,sapium_obovatum:6.160758)sapium:6.160714,stillingia_oppositifolia:12.321428):20.535715,(sebastiania_argutidens:6.571429,sebastiania_brasiliensis:6.571455,sebastiania_commersoniana:6.571455,sebastiania_edwalliana:6.571455,sebastiania_klotzschiana:6.571455,sebastiania_schottiana:6.571455,sebastiania_serrata:6.571455)sebastiania:26.285715):6.571426)h2:6.571430)hippomanoids:18.400000,(tetrorchidium_parvulum:10.733334,tetrorchidium_rubrivenium:10.733372)tetrorchidium:53.666668):4.599998,((((astraea_lobata:23.000000,(croton_celtidifolius:5.750000,croton_echinocarpus:5.750031,croton_floribundus:5.750031,croton_glandulosus:5.750031,croton_gracilipes:5.750031,croton_hemiargyreus:5.750031,croton_macrobothrys:5.750031,croton_organensis:5.750031,croton_piptocalyx:5.750031,croton_priscus:5.750031,croton_salutaris:5.750031,croton_triqueter:5.750031,croton_urucurana:5.750031,croton_vullnerarius:5.750031)croton:17.250000):15.333332,joannesia_princeps:38.333332)c1:7.666668,(pausandra_megalophylla:7.666667,pausandra_morisiana:7.666708)pausandra:38.333336)inaperturatecrotonoids:7.666668,(cnidoscolus_vitifolius:16.100000,(manihot_anomala:8.050000,manihot_esculenta:8.050036,manihot_grahamii:8.050036,manihot_inflata:8.050036,manihot_pilosa:8.050036,manihot_tripartita:8.050036)manihot:8.050000):37.566666):15.333332):4.599998):9.200005,((chaetocarpus_echinocarpus:20.700001,(pera_bicolor:10.350000,pera_glabrata:10.350042,pera_heteranthera:10.350042)pera:10.350000):41.400002,pogonophora_schomburgkiana:62.100002)peroideae:20.700001)euphorbiaceae:9.199998,(humiria_balsamifera:61.333332,(humiriastrum_dentatum:30.666683,humiriastrum_glaziovii:30.666683)humiriastrum:30.666683,vantanea_compacta:61.333366)humiriaceae:30.666666,((barnebya_dispar:51.428570,((bunchosia_armeniaca:5.714285,bunchosia_maritima:5.714334,bunchosia_pallescens:5.714334,bunchosia_paraguariensis:5.714334)bunchosia:40.000000,(heteropterys_byrsonimifolia:10.666666,heteropterys_hypericifolia:10.666708,heteropterys_pannosa:10.666708)heteropterys:35.047619):5.714283):8.571428,((byrsonima_bahiana:12.000000,byrsonima_basiloba:12.000031,byrsonima_coccolobifolia:12.000031,byrsonima_cydoniifolia:12.000031,byrsonima_intermedia:12.000031,byrsonima_laxiflora:12.000031,byrsonima_ligustrifolia:12.000031,byrsonima_myricifolia:12.000031,byrsonima_pachyphylla:12.000031,byrsonima_sericea:12.000031,byrsonima_stipulacea:12.000031,byrsonima_variabilis:12.000031,byrsonima_verbascifolia:12.000031)byrsonima:36.000000,lophanthera_lactescens:48.000000):12.000000)malpighiaceae:32.000000,((ouratea_castaneifolia:5.625000,ouratea_cuspidata:5.625031,ouratea_multiflora:5.625031,ouratea_parviflora:5.625031,ouratea_polygyna:5.625031,ouratea_semiserrata:5.625031,ouratea_spectabilis:5.625031,ouratea_vaccinioides:5.625031)ouratea:39.375000,quiina_glaziovii:45.000000)ochnaceae:47.000000,((((discocarpus_pedicellatus:25.714287,savia_dictyocarpa:25.714287)saviinae:28.285713,(margaritaria_nobilis:37.799999,(phyllanthus_acuminatus:9.450000,phyllanthus_sellowianus:9.450025)phyllanthus:28.350000)phyllantheae:16.200001)phyllanthoideae:6.750000,((hieronyma_alchorneoides:13.500000,hieronyma_oblonga:13.500031)hieronyma:27.000000,richeria_grandis:40.500000):20.250000)phyllanthaceae:6.750000,piranhea_securinega:67.500063):24.500000,drypetes_sessiliflora:92.000000)malpighiales:5.000000,(((connarus_detersus:19.666666,connarus_nodosus:19.666731,connarus_regnellii:19.666731,connarus_suberosus:19.666731)connarus:19.666666,(rourea_glabra:19.666698,rourea_martiana:19.666698)rourea:19.666698)connaraceae:41.666664,(((lamanonia_grandistipularis:4.833333,lamanonia_ternata:4.833384)lamanonia:14.500000,(weinmannia_humilis:3.222223,weinmannia_paulliniifolia:3.222266,weinmannia_pinnata:3.222266)weinmanniaamerica&mascarene:16.111111):48.666664,(sloanea_garckeana:14.000000,sloanea_guianensis:14.000031,sloanea_hirsuta:14.000031,sloanea_lasiocoma:14.000031)sloanea:54.000000):13.000000):16.000000):3.000000,((((cheiloclinium_anomalum:3.325000,cheiloclinium_cognatum:3.325024)cheiloclinium:9.975000,salacia_arborea:13.300000):6.650001,(salacia_elliptica:4.987500,salacia_impressifolia:4.987536,salacia_mosenii:4.987536)salacia:14.962501)salacioideae:32.299999,(((maytenus_ardisiaefolia:7.464286,maytenus_boaria:7.464334,maytenus_brasiliensis:7.464334,maytenus_cassineformis:7.464334,maytenus_communis:7.464334,maytenus_evonymoides:7.464334,maytenus_floribunda:7.464334,maytenus_glaucescens:7.464334,maytenus_gonoclada:7.464334,maytenus_ilicifolia:7.464334,maytenus_littoralis:7.464334,maytenus_longifolia:7.464334,maytenus_macrodonta:7.464334,maytenus_muelleri:7.464334,maytenus_obtusifolia:7.464334,maytenus_robusta:7.464334,maytenus_salicifolia:7.464334,maytenus_schumanniana:7.464334,maytenus_subalata:7.464334)maytenus:7.464286,plenckia_populnea:14.928572):29.857141,schaefferia_argentinensis:44.785713):7.464287):47.750000):3.000000,(((((((celtis_ehrenbergiana:9.250000,celtis_iguanaea:9.250031)celtis:9.250000,trema_micrantha:18.500000):33.000000,((((((brosimum_gaudichaudii:1.750000,brosimum_glaziovii:1.750031,brosimum_guianense:1.750031)brosimum:8.750000,brosimum_lactescens:10.500000):17.500000,((ficus_adhatodifolia:12.250000,ficus_americana:12.250031,ficus_calyptroceras:12.250031,ficus_cestrifolia:12.250031,ficus_citrifolia:12.250031,ficus_crocata:12.250031,ficus_dendrocida:12.250031,ficus_enormis:12.250031,ficus_eximia:12.250031,ficus_gomelleira:12.250031,ficus_hirsuta:12.250031,ficus_insipida:12.250031,ficus_luschnathiana:12.250031,ficus_mexiae:12.250031,ficus_nymphaeifolia:12.250031,ficus_obtusifolia:12.250031,ficus_obtusiuscula:12.250031,ficus_pallida:12.250031,ficus_pertusa:12.250031,ficus_pulchella:12.250031,ficus_trigona:12.250031)ficus:12.250000,((helicostylis_tomentosa:7.000000,pseudolmedia_hirtula:7.000000):3.500000,naucleopsis_oblongifolia:10.500000):14.000000):3.500000):3.500000,maclura_tinctoria:31.500000):3.500000,((clarisia_ilicifolia:3.645833,clarisia_racemosa:3.645884)clarisia:25.520834,(sorocea_bonplandii:11.666667,sorocea_guilleminiana:11.666707,sorocea_hilarii:11.666707,sorocea_sprucei:11.666707)sorocea:17.500000):5.833333)moraceae:8.250000,(((((coussapoa_curranii:8.020833,coussapoa_microcarpa:8.020885)coussapoa:8.020833,(cecropia_glaziovi:4.010417,cecropia_hololeuca:4.010458,cecropia_pachystachya:4.010458,cecropia_saxatilis:4.010458)cecropia:12.031250):8.020834,(pourouma_guianensis:12.031250,pourouma_velutina:12.031281)pourouma:12.031250)cladeIV:8.020832,boehmeria_caudata:32.083332):2.916667,(urera_baccifera:4.444444,urera_caracasana:4.444472,urera_nitida:4.444472)urera:30.555555)urticaceae:8.250000):8.250000):14.500000,(ampelocera_glabra:44.000000,(phyllostylon_brasiliense:22.000015,phyllostylon_rhamnoides:22.000015)phyllostylon:22.000015)ulmaceae:22.000000)bell0744:9.500000,((colubrina_glandulosa:35.424999,(ziziphus_glaziovii:4.843750,ziziphus_platyphylla:4.843781)ziziphus:30.581249):26.575001,(rhamnidium_elaeocarpum:27.600000,(rhamnus_sphaerosperma:13.800000,(scutia_arenicola:6.900000,scutia_buxifolia:6.900049)scutia:6.900000):13.800000)rhamneae:34.400002)rhamnaceae:13.500000)bell0738:9.500000,prunus_myrtifolia:85.000000)rosales:14.000000,(((((((((((((((((abarema_brachystachya:2.650000,abarema_filamentosum:2.650049,abarema_jupunba:2.650049)abarema:7.950000,(((albizia_edwallii:1.987500,albizia_inundata:1.987537,albizia_lebbeck:1.987537,albizia_niopoides:1.987537,albizia_pedicellaris:1.987537,albizia_polycephala:1.987537)albizia:1.987500,blanchetiodendron_blanchetii:3.975000):3.975000,(((inga_alba:1.325000,inga_bullata:1.325024,inga_capitata:1.325024,inga_ciliata:1.325024,inga_cylindrica:1.325024,inga_edulis:1.325024,inga_edwallii:1.325024,inga_heterophylla:1.325024,inga_hispida:1.325024,inga_ingoides:1.325024,inga_lanceifolia:1.325024,inga_laurina:1.325024,inga_lenticellata:1.325024,inga_lentiscifolia:1.325024,inga_marginata:1.325024,inga_organensis:1.325024,inga_pleiogyna:1.325024,inga_ruiziana:1.325024,inga_sellowiana:1.325024,inga_sessilis:1.325024,inga_striata:1.325024,inga_subnuda:1.325024,inga_tenuis:1.325024,inga_thibaudiana:1.325024,inga_vera:1.325024,inga_virescens:1.325024,inga_vulpina:1.325024)inga:1.325000,leucochloron_incuriale:2.650000):2.650000,zygia_latifolia:5.300000):2.650000):2.650000):2.650000,(calliandra_brevipes:1.325000,calliandra_foliolosa:1.325024,calliandra_parviflora:1.325024,calliandra_tweediei:1.325024)calliandra:11.924999):2.650001,((chloroleucon_tenuiflorum:6.360000,chloroleucon_tortum:6.360032)chloroleucon:6.360000,((enterolobium_contortisiliquum:3.180000,enterolobium_gummiferum:3.180046)enterolobium:6.360000,samanea_tubulosa:9.540000):3.180000):3.180000):7.950001,senegalia_polyphylla:23.850000):2.650000,(((microlobius_foetidus:6.625000,(parapiptadenia_pterosperma:3.312500,parapiptadenia_rigida:3.312531)parapiptadenia:3.312500):6.625000,(pseudopiptadenia_contorta:6.625000,pseudopiptadenia_inaequalis:6.625031,pseudopiptadenia_leptostachya:6.625031,pseudopiptadenia_warmingii:6.625031)pseudopiptadenia:6.625000,(stryphnodendron_adstringens:6.625000,stryphnodendron_guianense:6.625031,stryphnodendron_polyphyllum:6.625031,stryphnodendron_pulcherrinum:6.625031,stryphnodendron_rotundifolium:6.625031)stryphnodendron:6.625000):6.625000,((mimosa_artemisiana:4.968750,mimosa_bimucronata:4.968781,mimosa_glutinosa:4.968781,mimosa_scabrella:4.968781)mimosa:4.968750,(piptadenia_gonoacantha:4.968750,piptadenia_paniculata:4.968781)piptadenia:4.968750):9.937500):6.625000):2.650002,(anadenanthera_colubrina:7.287500,anadenanthera_peregrina:7.287542)anadenanthera:21.862501):5.300000,leucaena_leucocephala:34.450001):5.300000,plathymenia_reticulata:39.750000):10.816664,(dimorphandra_mollis:44.245834,(peltophorum_dubium:37.924999,schizolobium_parahyba:37.924999)peltophorumclade:6.320835,(tachigali_aurea:7.374306,tachigali_beaurepairei:7.374352,tachigali_denudata:7.374352,tachigali_multijuga:7.374352,tachigali_paratyensis:7.374352,tachigali_pilgeriana:7.374352,tachigali_rugosa:7.374352,tachigali_vulgaris:7.374352)tachigali:36.871529):6.320831,diptychandra_aurantiaca:50.566666):2.722225,(((caesalpinia_echinata:36.540951,(libidibia_ferrea:18.270475,poincianella_pluviosa:18.270475):18.270475):6.090160,guilandina_bonduc:42.631111)caesalpiniaclade:5.328892,((((cassia_ferruginea:7.993334,cassia_grandis:7.993361,cassia_leptophylla:7.993361)cassia:7.993334,(senna_affinis:7.993334,senna_alata:7.993361,senna_angulata:7.993361,senna_appendiculata:7.993361,senna_cernua:7.993361,senna_macranthera:7.993361,senna_multijuga:7.993361,senna_pendula:7.993361,senna_reticulata:7.993361,senna_rugosa:7.993361,senna_silvestris:7.993361,senna_spectabilis:7.993361,senna_splendida:7.993361)senna:7.993334):15.986668,(chamaecrista_ensiformis:23.980001,melanoxylon_brauna:23.980001):7.993334):7.993332,pterogyne_nitens:39.966667)cassiaclade:7.993336):5.328888):2.722221,gleditsia_amorphoides:56.011112)caesalpinieaegrade:2.722221,((((((abrus_precatorius:40.177780,(((dahlstedtia_pentaphylla:2.511111,dahlstedtia_pinnata:2.511136)dahlstedtia:2.511111,(lonchocarpus_campestris:2.511111,lonchocarpus_cultratus:2.511136,lonchocarpus_filipes:2.511136,lonchocarpus_muehlbergianus:2.511136,lonchocarpus_nitidus:2.511136,lonchocarpus_sericeus:2.511136,lonchocarpus_subglaucescens:2.511136,lonchocarpus_virgilioides:2.511136)lonchocarpus:2.511111):10.044445,deguelia_hatschbachii:15.066667,derris_floribundus:15.066666):25.111115):5.022223,(erythrina_cristagalli:4.591667,erythrina_falcata:4.591713,erythrina_speciosa:4.591713,erythrina_verna:4.591713)erythrina:40.608334,platycyamus_regnellii:45.200001)milletioids:6.781246,sesbania_sesban:51.981313):4.143752,(((((acosmium_cardenasii:15.733334,acosmium_lentiscifolium:15.733372)acosmium:15.733334,(((centrolobium_robustum:7.866667,centrolobium_sclerophyllum:7.866701,centrolobium_tomentosum:7.866701)centrolobium:13.111111,(pterocarpus_montanus:5.244444,pterocarpus_rohrii:5.244478)pterocarpus:15.733334):5.244446,platypodium_elegans:26.222223):5.244444):5.244446,stylosanthes_viscosa:36.711113):5.244442,(platymiscium_floribundum:20.977777,platymiscium_luteum:20.977819,platymiscium_pubescens:20.977819)platymiscium:20.977777):6.994444,((dalbergia_brasiliensis:12.160000,dalbergia_ecastaphyllum:12.160038,dalbergia_foliolosa:12.160038,dalbergia_miscolobium:12.160038,dalbergia_nigra:12.160038,dalbergia_villosa:12.160038)dalbergia:12.160000,(machaerium_acutifolium:6.080000,machaerium_amplum:6.080034,machaerium_brasiliense:6.080034,machaerium_condensatum:6.080034,machaerium_eriocarpum:6.080034,machaerium_floridum:6.080034,machaerium_hatschbachii:6.080034,machaerium_hirtum:6.080034,machaerium_incorruptibile:6.080034,machaerium_isadelphum:6.080034,machaerium_leucopterum:6.080034,machaerium_nyctitans:6.080034,machaerium_opacum:6.080034,machaerium_paraguariense:6.080034,machaerium_pedicellatum:6.080034,machaerium_puntactum:6.080034,machaerium_scleroxylon:6.080034,machaerium_stipitatum:6.080034,machaerium_uncinatum:6.080034,machaerium_villosum:6.080034)machaerium:18.240000):24.630001):7.174999,((andira_anthelmia:14.031250,andira_fraxinifolia:14.031281,andira_inermis:14.031281,andira_legalis:14.031281,andira_nitida:14.031281,andira_ormosioides:14.031281)andira:14.031250,hymenolobium_janeirense:28.062500)andiraclade:28.062500,(((bowdichia_virgilioides:17.889843,(leptolobium_bijugum:8.944921,leptolobium_dasycarpum:8.944971,leptolobium_elegans:8.944971)leptolobium:8.944921):29.816406,(crotalaria_vitellina:42.093750,sophora_tomentosa:42.093750):5.612500,poecilanthe_parviflora:47.706249):5.612500,(ormosia_arborea:19.994530,ormosia_fastigiata:19.994576,ormosia_friburgensis:19.994576)ormosia:33.324219)genistoidslclade:2.806252,(exostyles_venusta:37.416668,holocalyx_balansae:37.416668,(zollernia_glabra:18.708334,zollernia_glaziovii:18.708384,zollernia_ilicifolia:18.708384)zollernia:18.708334)lecointeoidclade:18.708332,(((luetzelburgia_auriculata:9.354167,luetzelburgia_trialata:9.354207)luetzelburgia:9.354167,vatairea_macrocarpa:18.708334):18.708334,sweetia_fruticosa:37.416668,vataireopsis_araroba:37.416668)vataireoidclade:18.708332):1.649998,(ateleia_glazioveana:46.219997,((swartzia_acutifolia:11.554999,swartzia_flaemingii:11.555047,swartzia_langsdorffii:11.555047,swartzia_macrostachya:11.555047,swartzia_multijuga:11.555047,swartzia_myrtifolia:11.555047,swartzia_polyphylla:11.555047,swartzia_simplex:11.555047)swartzia:11.554999,swartzia_apetala:23.109999):23.109999)swartzioidclade:11.555000):0.825000,((amburana_cearensis:29.299999,(myrocarpus_frondosus:7.325000,myroxylon_peruiferum:7.325000):21.974998):14.649998,dipteryx_alata:43.949997):14.650001)papilionoideae:0.133335):0.133331,(apuleia_leiocarpa:12.857142,martiodendron_mediterraneum:12.857142):46.009521):0.133334,((bauhinia_acuruana:3.777777,bauhinia_brevipes:3.777826,bauhinia_forficata:3.777826,bauhinia_longifolia:3.777826,bauhinia_pentandra:3.777826,bauhinia_rufa:3.777826,bauhinia_ungulata:3.777826)bauhinia:42.722221,(((copaifera_langsdorffii:4.380000,copaifera_lucens:4.380040,copaifera_trapezifolia:4.380040)copaifera:16.060001,((guibourtia_hymenaefolia:5.110000,(hymenaea_aurea:2.555000,hymenaea_courbaril:2.555046,hymenaea_rubriflora:2.555046,hymenaea_stigonocarpa:2.555046)hymenaea:2.555000):10.220000,(peltogyne_angustiflora:7.665000,peltogyne_discolor:7.665048,peltogyne_mattosiana:7.665048)peltogyne:7.665000):5.110001)detarieaessclade:8.760000,macrolobium_latifolium:29.200001)detarieae:17.299999):12.500000)fabaceae:12.000000,(acanthocladus_albicans:23.0,acanthocladus_brasiliensis:23.0,acanthocladus_pulcherrimus:23.0)acanthocladus:48.000063):12.000000,quillaja_brasiliensis:83.000000)fabales:16.000000)nitrogen_fixing_clade:4.000000):7.500000,((((((anacardium_occidentale:42.666668,(astronium_concinnum:21.333363,astronium_fraxinifolium:21.333363,astronium_graveolens:21.333363,astronium_nelsonrosae:21.333363)astronium:21.333363,(lithrea_brasiliensis:21.333363,lithrea_molleoides:21.333363)lithrea:21.333363,(myracrodruon_balansae:21.333363,myracrodruon_urundeuva:21.333363)myracrodruon:21.333363,schinopsis_brasiliensis:42.666725,(schinus_lentiscifolius:21.333363,schinus_molle:21.333363,schinus_polygamus:21.333363,schinus_terebinthifolius:21.333363,schinus_weinmannifolius:21.333363)schinus:21.333363,(tapirira_guianensis:21.333363,tapirira_obtusa:21.333363)tapirira:21.333363,thyrsodium_spruceanum:42.666725)anacardiaceae:21.333334,((commiphora_leptophloeos:36.571430,(protium_brasiliense:13.714286,protium_heptaphyllum:13.714334,protium_icicariba:13.714334,protium_kleinii:13.714334,protium_spruceanum:13.714334,protium_warmingiana:13.714334,protium_widgrenii:13.714334)protium:22.857145):9.142857,trattinnickia_ferruginea:45.714287):18.285713)bell0930:3.500000,(((((cabralea_canjerana:16.625000,(guarea_guidonia:2.078125,guarea_kunthiana:2.078156,guarea_macrophylla:2.078156,guarea_pendula:2.078156)guarea:14.546875):11.875000,(trichilia_catigua:8.906250,trichilia_claussenii:8.906281,trichilia_elegans:8.906281,trichilia_hirta:8.906281,trichilia_lepidota:8.906281,trichilia_pallens:8.906281,trichilia_pallida:8.906281,trichilia_pseudostipularis:8.906281,trichilia_silvatica:8.906281)trichilia:19.593750):9.500000,(cedrela_fissilis:3.257143,cedrela_odorata:3.257170)cedrela:34.742859)meliaceae:7.500000,(almeidea_rubra:40.0,balfourodendron_riedelianum:40.0,(conchocarpus_heterophyllus:20.0,conchocarpus_odoratissimus:20.0,conchocarpus_pentandrus:20.0)conchocarpus:20.0,dictyoloma_vandellianum:40.0,(esenbeckia_febrifuga:20.0,esenbeckia_grandiflora:20.0,esenbeckia_leiocarpa:20.0,esenbeckia_pilocarpoides:20.0)esenbeckia:20.0,(galipea_jasminiflora:20.0,galipea_laxiflora:20.0)galipea:20.0,(helietta_apiculata:20.0,helietta_puberula:20.0)helietta:20.0,hortia_brasiliana:40.0,(metrodorea_nigra:20.0,metrodorea_stipularis:20.0)metrodorea:20.0,(neoraputia_alba:20.0,neoraputia_magnifica:20.0)neoraputia:20.0,(pilocarpus_giganteus:20.0,pilocarpus_pauciflorus:20.0,pilocarpus_pennatifolius:20.0,pilocarpus_riedelianus:20.0,pilocarpus_spicatus:20.0)pilocarpus:20.0,(rauia_nodosa:20.0,rauia_resinosa:20.0)rauia:20.0,(zanthoxylum_acuminatum:20.0,zanthoxylum_caribaeum:20.0,zanthoxylum_fagara:20.0,zanthoxylum_kleinii:20.0,zanthoxylum_monogynum:20.0,zanthoxylum_petiolare:20.0,zanthoxylum_rhoifolium:20.0,zanthoxylum_riedelianum:20.0,zanthoxylum_rigidum:20.0,zanthoxylum_tingoassuiba:20.0)zanthoxylum:20.0)rutaceae:5.500062,((castela_tweedii:27.805555,picrasma_crenata:27.805555):13.902777,((simaba_floribunda:5.055556,simaba_subcymosa:5.055589)simaba:5.055556,(simarouba_amara:2.527778,simarouba_versicolor:2.527825)simarouba:7.583334):31.597219)simaroubaceae:3.791668):5.500000,((((((allophylus_edulis:2.411484,allophylus_guaraniticus:2.411515,allophylus_heterophyllus:2.411515,allophylus_petiolulatus:2.411515,allophylus_puberulus:2.411515,allophylus_racemosus:2.411515,allophylus_semidentatus:2.411515,allophylus_sericeus:2.411515)allophylus:12.057416,diatenopteryx_sorbifolia:14.468900):4.822967,((dilodendron_bipinnatum:4.822967,(talisia_coriacea:2.411484,talisia_esculenta:2.411515)talisia:2.411484):9.645934,(melicoccus_lepidopetalus:7.234450,melicoccus_oliviformis:7.234483)melicoccus:7.234450)melicoccusgroup:4.822967):7.234450,(((cupania_castaneaefolia:1.289474,cupania_emarginata:1.289506,cupania_furfuracea:1.289506,cupania_ludowigii:1.289506,cupania_oblongifolia:1.289506,cupania_paniculata:1.289506,cupania_racemosa:1.289506,cupania_tenuivalvis:1.289506,cupania_vernalis:1.289506,cupania_zanthoxyloides:1.289506)cupania:3.868421,(matayba_cristae:1.289474,matayba_elaeagnoides:1.289506,matayba_elegans:1.289506,matayba_guianensis:1.289506,matayba_juglandifolia:1.289506)matayba:3.868421):16.947369,tripterodendron_filicifolium:22.105286)cupaniagroup:4.421053):2.210526,(sapindus_saponaria:4.190789,toulicia_laevigata:4.190789):24.546051):8.842108,(averrhoidium_paraguaiense:30.063160,dodonaea_viscosa:30.063160)dodonaeagroup:7.515789):13.421052)bell0929:16.500000):24.699997,((((bixa_arborea:17.888889,bixa_orellana:17.888939)bixa:35.777779,((((((((abutilon_bedfordianum:3.412500,abutilon_peltatum:3.412543)abutilon:3.412500,bastardiopsis_densiflora:6.825000):16.331249,(peltaea_obsita:20.261749,(pavonia_alnifolia:1.447266,pavonia_malacophylla:1.447296,pavonia_sepium:1.447296)pavonia:18.814451)hybisceae:2.894531)eumalvoideae:4.875000,(quararibea_penduliflora:9.343750,quararibea_turbinata:9.343781)quararibea:18.687500):2.437500,(cavanillesia_umbellata:21.328125,(((ceiba_crispiflora:2.539063,ceiba_erianthos:2.539093,ceiba_pubiflora:2.539093,ceiba_speciosa:2.539093)ceiba:7.617188,(pseudobombax_endecaphyllum:5.078125,pseudobombax_grandiflorum:5.078156,pseudobombax_longiflorum:5.078156,pseudobombax_tomentosum:5.078156)pseudobombax:5.078125):5.078125,(((eriotheca_candolleana:1.523438,eriotheca_gracilipes:1.523468,eriotheca_macrophylla:1.523468,eriotheca_pentaphylla:1.523468)eriotheca:1.523438,pachira_stenopetala:3.046875):9.140625,spirotheca_rivieri:12.187500):3.046875):6.093750):9.140625):2.437500,((sterculia_apetala:13.710938,sterculia_striata:13.710968)sterculia:13.710938,basiloxylon_brasiliensis:27.421906)sterculioideae:5.484375):3.656250,(helicteres_lhotzkyana:4.570313,helicteres_ovata:4.570343,helicteres_pentandra:4.570343)helicteres:31.992188):2.437500,(((apeiba_tibourbou:24.311687,(heliocarpus_americanus:6.077922,triumfetta_semitriloba:6.077922):18.233765)apeibaclade:6.077921,(luehea_candicans:9.496753,luehea_conwentsii:9.496778,luehea_divaricata:9.496778,luehea_grandiflora:9.496778,luehea_paniculata:9.496778)luehea:20.892857)grewioideae:5.064936,(guazuma_crinita:11.965909,guazuma_ulmifolia:11.965945)guazuma:23.488634):3.545455)malvaceae:14.666668):14.666668,(daphnopsis_brasiliensis:18.0,daphnopsis_coriacea:18.0,daphnopsis_fasciculata:18.0,daphnopsis_martii:18.0,daphnopsis_racemosa:18.0,daphnopsis_schwackeana:18.0,daphnopsis_sellowiana:18.0)daphnopsis:50.333393):17.733334,((crataeva_tapia:28.666666,(cynophalla_flexuosa:14.333364,cynophalla_retusa:14.333364)cynophalla:14.333364,mesocapparis_lineata:28.666729,monilicarpa_brasiliana:28.666729)capparaceae:45.833332,((jacaratia_corumbensis:12.7,jacaratia_heptaphylla:12.7,jacaratia_spinosa:12.7)jacaratia:6.10006,vasconcellea_quercifolia:18.80006)jaca_vasco:55.700001):11.566666):6.133332):3.066666,(picramnia_bahiensis:31.755554,picramnia_ciliata:31.755585,picramnia_gardneri:31.755585,picramnia_glazioviana:31.755585,picramnia_parvifolia:31.755585,picramnia_ramiflora:31.755585,picramnia_sellowii:31.755585)picramnia:63.511108):9.400002,((((buchenavia_kleinii:1.500000,buchenavia_tetraphylla:1.500031)buchenavia:4.500000,(terminalia_acuminata:3.000000,terminalia_argentea:3.000031,terminalia_australis:3.000031,terminalia_catappa:3.000031,terminalia_glabrescens:3.000031,terminalia_januarensis:3.000031,terminalia_phaeocarpa:3.000031,terminalia_triflora:3.000031)terminalia:3.000000):12.000000,combretum_duarteanum:17.999998)combreteae:71.000000,((lafoensia_glyptocarpa:25.0,lafoensia_pacari:25.0,lafoensia_vandelliana:25.0)lafoensia:63.250063,(((((((clidemia_capitellata:15.789474,(leandra_aurea:3.157895,leandra_barbinervis:3.157932,leandra_carassana:3.157932,leandra_dasytricha:3.157932,leandra_fallax:3.157932,leandra_glabrata:3.157932,leandra_glazioviana:3.157932,leandra_lacunosa:3.157932,leandra_lancifolia:3.157932,leandra_melastomoides:3.157932,leandra_pectinata:3.157932,leandra_regnellii:3.157932,leandra_sericea:3.157932,leandra_variabilis:3.157932)leandra:12.631579):11.278195,(miconia_affinis:8.458647,miconia_albicans:8.458681,miconia_argyrophylla:8.458681,miconia_brevipes:8.458681,miconia_brunnea:8.458681,miconia_budlejoides:8.458681,miconia_cabucu:8.458681,miconia_calvescens:8.458681,miconia_castaneiflora:8.458681,miconia_chamissois:8.458681,miconia_chartacea:8.458681,miconia_cinerascens:8.458681,miconia_cinnamomifolia:8.458681,miconia_collatata:8.458681,miconia_corallina:8.458681,miconia_cubatanensis:8.458681,miconia_discolor:8.458681,miconia_divaricata:8.458681,miconia_dodecandra:8.458681,miconia_eichlerii:8.458681,miconia_elegans:8.458681,miconia_fasciculata:8.458681,miconia_holosericea:8.458681,miconia_hyemalis:8.458681,miconia_hypoleuca:8.458681,miconia_inaequidens:8.458681,miconia_inconspicua:8.458681,miconia_latecrenata:8.458681,miconia_lepidota:8.458681,miconia_ligustroides:8.458681,miconia_minutiflora:8.458681,miconia_paucidens:8.458681,miconia_pepericarpa:8.458681,miconia_prasina:8.458681,miconia_pusilliflora:8.458681,miconia_pyrifolia:8.458681,miconia_rimalis:8.458681,miconia_rubiginosa:8.458681,miconia_sellowiana:8.458681,miconia_stenostachya:8.458681,miconia_theizans:8.458681,miconia_trianae:8.458681,miconia_tristis:8.458681,miconia_urophylla:8.458681,miconia_valtherii:8.458681,miconia_willdenowii:8.458681)miconia:18.609022):13.533836,(meriania_claussenii:5.075188,meriania_glabra:5.075233,meriania_paniculata:5.075233)meriania:35.526318):2.255638,((huberia_laurina:2.816327,huberia_nettoana:2.816364,huberia_semiserrata:2.816364)huberia:36.612244,((rhynchanthera_dichotoma:26.938776,trembleya_parviflora:26.938776)microliceae:10.775510,(tibouchina_arborea:15.428572,tibouchina_candolleana:15.428606,tibouchina_clavata:15.428606,tibouchina_estrellensis:15.428606,tibouchina_fothergillae:15.428606,tibouchina_granulosa:15.428606,tibouchina_heteromalla:15.428606,tibouchina_moricandiana:15.428606,tibouchina_mutabilis:15.428606,tibouchina_pulchra:15.428606,tibouchina_reitzii:15.428606,tibouchina_sellowiana:15.428606,tibouchina_semidecandra:15.428606,tibouchina_stenocarpa:15.428606,tibouchina_trichopoda:15.428606,tibouchina_urvilleana:15.428606)tibouchina:22.285715):1.714283):3.428574):1.714287,(henriettea_glabra:11.142858,henriettea_succosa:11.142909)henriettea:33.428574):3.428569,(mouriri_arborea:6.000000,mouriri_chamissoana:6.000031,mouriri_glazioviana:6.000031)mouriri:42.000000)melastomataceae:39.500000,((((((acca_sellowiana:6.800000,((campomanesia_dichotoma:1.700000,campomanesia_eugenioides:1.700024,campomanesia_guaviroba:1.700024,campomanesia_guazumifolia:1.700024,campomanesia_laurifolia:1.700024,campomanesia_phaea:1.700024,campomanesia_pubescens:1.700024,campomanesia_rhombea:1.700024,campomanesia_rufa:1.700024,campomanesia_schlechtendahliana:1.700024,campomanesia_simulans:1.700024,campomanesia_velutina:1.700024,campomanesia_xanthocarpa:1.700024)campomanesia:1.700000,(psidium_brownianum:1.700000,psidium_cattleianum:1.700024,psidium_grandifolium:1.700024,psidium_guajava:1.700024,psidium_guineense:1.700024,psidium_longipetiolatum:1.700024,psidium_myrtoides:1.700024,psidium_ovale:1.700024,psidium_rufum:1.700024,psidium_sartorianum:1.700024)psidium:1.700000):3.400000,myrrhinium_atropurpureum:6.800000):3.400000,pimenta_pseudocaryophyllus:10.200000):6.800001,((eugenia_acutata:4.250000,eugenia_arenaria:4.250031,eugenia_astringens:4.250031,eugenia_aurata:4.250031,eugenia_bacopari:4.250031,eugenia_bahiensis:4.250031,eugenia_biflora:4.250031,eugenia_bimarginata:4.250031,eugenia_bocainensis:4.250031,eugenia_brasiliensis:4.250031,eugenia_brevistyla:4.250031,eugenia_bunchosiifolia:4.250031,eugenia_burkartiana:4.250031,eugenia_cambucae:4.250031,eugenia_capitulifera:4.250031,eugenia_catharinae:4.250031,eugenia_cerasiflora:4.250031,eugenia_cereja:4.250031,eugenia_chlorophylla:4.250031,eugenia_copacabanensis:4.250031,eugenia_cuprea:4.250031,eugenia_cymatodes:4.250031,eugenia_dimorpha:4.250031,eugenia_disperma:4.250031,eugenia_dodonaeaefolia:4.250031,eugenia_dysenterica:4.250031,eugenia_egensis:4.250031,eugenia_excelsa:4.250031,eugenia_expansa:4.250031,eugenia_flavescens:4.250031,eugenia_florida:4.250031,eugenia_francavilleana:4.250031,eugenia_fusca:4.250031,eugenia_handroana:4.250031,eugenia_handroi:4.250031,eugenia_hyemalis:4.250031,eugenia_involucrata:4.250031,eugenia_itajurensis:4.250031,eugenia_klotzschiana:4.250031,eugenia_leptoclada:4.250031,eugenia_ligustrina:4.250031,eugenia_longipedunculata:4.250031,eugenia_macahensis:4.250031,eugenia_macedoi:4.250031,eugenia_macrosperma:4.250031,eugenia_magnibracteolata:4.250031,eugenia_magnifica:4.250031,eugenia_mandioccensis:4.250031,eugenia_mansoi:4.250031,eugenia_melanogyna:4.250031,eugenia_monosperma:4.250031,eugenia_multicostata:4.250031,eugenia_myrcianthes:4.250031,eugenia_neoglomerata:4.250031,eugenia_neomyrtifolia:4.250031,eugenia_neosilvestris:4.250031,eugenia_neotristis:4.250031,eugenia_neoverrucosa:4.250031,eugenia_nutans:4.250031,eugenia_oxyoentophylla:4.250031,eugenia_peruibensis:4.250031,eugenia_pisiformis:4.250031,eugenia_pitanga:4.250031,eugenia_platysema:4.250031,eugenia_pluriflora:4.250031,eugenia_prasina:4.250031,eugenia_pruinosa:4.250031,eugenia_pruniformis:4.250031,eugenia_punicifolia:4.250031,eugenia_pyriformis:4.250031,eugenia_ramboi:4.250031,eugenia_repanda:4.250031,eugenia_rostrata:4.250031,eugenia_rostrifolia:4.250031,eugenia_schottiana:4.250031,eugenia_sclerocalyx:4.250031,eugenia_sonderiana:4.250031,eugenia_speciosa:4.250031,eugenia_sphenophylla:4.250031,eugenia_stigmatosa:4.250031,eugenia_subavenia:4.250031,eugenia_subterminalis:4.250031,eugenia_sulcata:4.250031,eugenia_supraaxillaris:4.250031,eugenia_tenuipedunculata:4.250031,eugenia_ternatifolia:4.250031,eugenia_umbellata:4.250031,eugenia_umbrosa:4.250031,eugenia_uniflora:4.250031,eugenia_uruguayensis:4.250031,eugenia_verticillata:4.250031)eugenia:4.250000,(myrcianthes_gigantea:4.250000,myrcianthes_pungens:4.250031)myrcianthes:4.250000)eugeniagroup:8.500000):6.800000,(((((calyptranthes_brasiliensis:4.250000,calyptranthes_clusiifolia:4.250031,calyptranthes_concinna:4.250031,calyptranthes_grandifolia:4.250031,calyptranthes_lanceolata:4.250031,calyptranthes_lucida:4.250031,calyptranthes_pileata:4.250031,calyptranthes_rubella:4.250031,calyptranthes_strigipes:4.250031,calyptranthes_tricona:4.250031,calyptranthes_widgreniana:4.250031)calyptranthes:4.250000,marlierea_eugeniopsoides:8.500000):4.250000,(((marlierea_antonia:2.125000,marlierea_dimorpha:2.125031,marlierea_excoriata:2.125031,marlierea_glabra:2.125031,marlierea_involucrata:2.125031,marlierea_krapovickae:2.125031,marlierea_neuwiediana:2.125031,marlierea_obscura:2.125031,marlierea_racemosa:2.125031,marlierea_reitzii:2.125031,marlierea_schottii:2.125031,marlierea_silvatica:2.125031,marlierea_skortzoviana:2.125031,marlierea_suaveolens:2.125031,marlierea_tomentosa:2.125031)marlierea:2.125000,myrcia_multiflora:4.250000):4.250000,myrcia_racemosa:8.500000):4.250000,(myrcia_aethusa:6.375000,myrcia_albotomentosa:6.375031,myrcia_amazonica:6.375031,myrcia_anacardiifolia:6.375031,myrcia_anceps:6.375031,myrcia_bella:6.375031,myrcia_bergiana:6.375031,myrcia_brasiliensis:6.375031,myrcia_cordiifolia:6.375031,myrcia_diaphana:6.375031,myrcia_dichrophylla:6.375031,myrcia_eriocalyx:6.375031,myrcia_eriopus:6.375031,myrcia_fallax:6.375031,myrcia_fenzliana:6.375031,myrcia_flagellaris:6.375031,myrcia_glabra:6.375031,myrcia_graciliflora:6.375031,myrcia_grandifolia:6.375031,myrcia_guianensis:6.375031,myrcia_hartwegiana:6.375031,myrcia_hatschbachii:6.375031,myrcia_hebepetala:6.375031,myrcia_heringii:6.375031,myrcia_hexasticha:6.375031,myrcia_ilheosensis:6.375031,myrcia_insularis:6.375031,myrcia_lajeana:6.375031,myrcia_laruotteana:6.375031,myrcia_macrocarpa:6.375031,myrcia_oblongata:6.375031,myrcia_obovata:6.375031,myrcia_oligantha:6.375031,myrcia_ovata:6.375031,myrcia_palustris:6.375031,myrcia_plusiantha:6.375031,myrcia_pubiflora:6.375031,myrcia_pubipetala:6.375031,myrcia_pulchra:6.375031,myrcia_recurvata:6.375031,myrcia_retorta:6.375031,myrcia_rufipes:6.375031,myrcia_rufula:6.375031,myrcia_selloi:6.375031,myrcia_splendens:6.375031,myrcia_squamata:6.375031,myrcia_subsericea:6.375031,myrcia_tenuivenosa:6.375031,myrcia_thyrsoidea:6.375031,myrcia_tijucensis:6.375031,myrcia_tomentosa:6.375031,myrcia_variabilis:6.375031,myrcia_venulosa:6.375031)myrcia:6.375000)myrciagroup:4.250000,((myrciaria_cuspidata:5.100000,myrciaria_delicatula:5.100043,myrciaria_floribunda:5.100043,myrciaria_glanduliflora:5.100043,myrciaria_glomerata:5.100043,myrciaria_plinioides:5.100043,myrciaria_strigipes:5.100043,myrciaria_tenella:5.100043)myrciaria:5.100000,(((neomitranthes_cordifolia:1.700000,neomitranthes_gemballae:1.700024,neomitranthes_glomerata:1.700024,neomitranthes_obscura:1.700024,neomitranthes_warmingiana:1.700024)neomitranthes:1.700000,(siphoneugena_crassifolia:1.700000,siphoneugena_densiflora:1.700024,siphoneugena_guilfoyleiana:1.700024,siphoneugena_kiaerskoviana:1.700024,siphoneugena_kuhlmannii:1.700024,siphoneugena_reitzii:1.700024)siphoneugena:1.700000):3.400000,(plinia_cauliflora:3.400000,plinia_complanata:3.400049,plinia_edulis:3.400049,plinia_grandifolia:3.400049,plinia_peruviana:3.400049,plinia_pseudodichasiantha:3.400049,plinia_rivularis:3.400049)plinia:3.400000):3.400000)pliniagroup:6.800001):3.400000,(myrceugenia_acutiflora:6.800000,myrceugenia_alpigena:6.800037,myrceugenia_bracteosa:6.800037,myrceugenia_brevipedicellata:6.800037,myrceugenia_campestris:6.800037,myrceugenia_cucullata:6.800037,myrceugenia_euosma:6.800037,myrceugenia_foveolata:6.800037,myrceugenia_glaucescens:6.800037,myrceugenia_kleinii:6.800037,myrceugenia_mesomischa:6.800037,myrceugenia_miersiana:6.800037,myrceugenia_myrcioides:6.800037,myrceugenia_myrtoides:6.800037,myrceugenia_ovalifolia:6.800037,myrceugenia_ovata:6.800037,myrceugenia_oxysepala:6.800037,myrceugenia_pilotantha:6.800037,myrceugenia_reitzii:6.800037,myrceugenia_rufescens:6.800037,myrceugenia_seriatoramosa:6.800037)myrceugenia:13.600000):3.400000):3.400002,blepharocalyx_salicifolius:27.200001):59.549999,(((callisthene_fasciculata:8.675000,callisthene_major:8.675036,callisthene_minor:8.675036)callisthene:26.025002,(qualea_cordata:17.350000,qualea_cryptantha:17.350042,qualea_dichotoma:17.350042,qualea_glaziovii:17.350042,qualea_grandiflora:17.350042,qualea_multiflora:17.350042,qualea_selloi:17.350042)qualea:17.350000):17.349998,(vochysia_acuminata:13.012500,vochysia_bifalcata:13.012525,vochysia_cinnamomea:13.012525,vochysia_glazioviana:13.012525,vochysia_magnifica:13.012525,vochysia_oppugnata:13.012525,vochysia_rectiflora:13.012525,vochysia_schwackeana:13.012525,vochysia_thyrsoidea:13.012525,vochysia_tucanorum:13.012525)vochysia:39.037498):34.700001):0.750000):0.750000):0.750000)myrtales:15.666664)malvidae:5.833336):10.500000,(curatella_americana:36.250000,davilla_elliptica:36.250000):84.750000)pentapetalae:7.333328,(((euplassa_cantareirae:6.145000,euplassa_incana:6.145039,euplassa_itatiaiae:6.145039,euplassa_legalis:6.145039,euplassa_organensis:6.145039,euplassa_rufa:6.145039)euplassa:44.084999,panopsis_rubescens:50.230003)macadamieae:23.170002,(roupala_consimilis:13.107143,roupala_longepetiolata:13.107182,roupala_montana:13.107182,roupala_paulensis:13.107182)roupala:60.292858)grevilleoideae:54.933331,(meliosma_itatiaiae:44.5,meliosma_sellowii:44.5,meliosma_sinuata:44.5)meliosma:83.833391):0.666667,berberis_laurina:129.000058)eudicots:32.666672,((((((acrocomia_aculeata:35.400002,desmoncus_orthacanthos:35.400002)acro&desmo:3.599998,(astrocaryum_aculeatissimum:33.300061,(bactris_acanthocarpa:16.650000,bactris_bahiensis:16.650049,bactris_caryotifolia:16.650049,bactris_hirta:16.650049,bactris_setosa:16.650049,bactris_vulgaris:16.650049)bactris:16.650000)bact&astro:5.700001)bactridinae:16.660000,(((allagoptera_arenaria:5.1,allagoptera_caudescens:5.1)allagoptera:27.700062,(attalea_dubia:13.0,attalea_phalerata:13.0)attalea:19.800062)atta&others:5.600002,(lytocaryum_hoehnei:21.400062,(syagrus_botryophora:16.6,syagrus_oleracea:16.6,syagrus_pseudococos:16.6,syagrus_romanzoffiana:16.6,syagrus_schizophylla:16.6)syagrus:4.800062)syag&others:17.000002)americanattaleinae:17.259998):17.939997,(euterpe_edulis:37.125000,(geonoma_brevispatha:18.1,geonoma_gamiova:18.1,geonoma_pauciflora:18.1,geonoma_pohliana:18.1,geonoma_schottiana:18.1)geonoma:19.025061):36.474998)arecoideae:13.266670,copernicia_alba:86.866669):23.300005,(cordyline_dracaenoides:28.0,cordyline_fruticosa:28.0,cordyline_spectabilis:28.0,cordyline_stricta:28.0)cordyline:82.166734):51.499996):5.666656,(((((((anaxagorea_dolichocarpa:12.500000,anaxagorea_phaeocarpa:12.500031,anaxagorea_silvatica:12.500031)anaxagorea:12.500000,((((((annona_acutiflora:2.812500,annona_cacans:2.812531,annona_coriacea:2.812531,annona_crassiflora:2.812531,annona_dolabripetala:2.812531,annona_emarginata:2.812531,annona_glabra:2.812531,annona_maritima:2.812531,annona_mucosa:2.812531,annona_neosalicifolia:2.812531,annona_neosericea:2.812531,annona_rugulosa:2.812531,annona_sylvatica:2.812531,annona_xylopiifolia:2.812531)annona:12.812500,(guatteria_australis:7.812500,guatteria_campestris:7.812531,guatteria_candolleana:7.812531,guatteria_latifolia:7.812531,guatteria_macropus:7.812531,guatteria_pogonopus:7.812531,guatteria_schomburgkiana:7.812531,guatteria_sellowiana:7.812531,guatteria_villosissima:7.812531)guatteria:7.812500):1.562500,(duguetia_furfuracea:2.864583,duguetia_lanceolata:2.864604,duguetia_pohliana:2.864604,duguetia_salicifolia:2.864604,duguetia_sessilis:2.864604)duguetia:14.322917):1.562500,(xylopia_aromatica:4.687500,xylopia_brasiliensis:4.687531,xylopia_emarginata:4.687531,xylopia_langsdorffiana:4.687531,xylopia_sericea:4.687531)xylopia:14.062500):1.562500,(cardiopetalum_calophyllum:15.234406,(cymbopetalum_brasiliense:5.078125,porcelia_macrocarpa:5.078125):10.156250)bocageeae:5.078125)annonoideae:1.562500,((oxandra_martiana:4.474432,oxandra_nitida:4.474482)oxandra:11.186080,unonopsis_guatterioides:15.660512)malmeeae:6.214488):3.125000)annonaceae:44.000000,magnolia_ovata:69.000058):7.000000,(bicuiba_oleifera:7.071429,(virola_bicuhyba:1.767857,virola_gardneri:1.767879,virola_sebifera:1.767879)virola:5.303571):68.928574)magnoliales:44.500000,(((((((((licaria_armeniaca:3.152951,licaria_triandra:3.152987)licaria:3.152951,(aniba_canelilla:3.152951,aniba_firmula:3.152987,aniba_viridis:3.152987)aniba:3.152951):12.611806,(phyllostemonodaphne_geminiflora:7.567083,urbanodendron_verrucosum:7.567083):11.350627):3.783541,((((endlicheria_glomerata:6.191250,endlicheria_paniculata:6.191250):2.063750,rhodostemonodaphne_capixabensis:8.255000,rhodostemonodaphne_macrocalyx:8.255000):8.254999,ocotea_diospyrifolia:16.510000):4.127500,(nectandra_angustifolia:5.159375,nectandra_barbellata:5.159411,nectandra_cissiflora:5.159411,nectandra_cuspidata:5.159411,nectandra_gardneri:5.159411,nectandra_grandiflora:5.159411,nectandra_hihua:5.159411,nectandra_lanceolata:5.159411,nectandra_leucantha:5.159411,nectandra_megapotamica:5.159411,nectandra_nitidula:5.159411,nectandra_oppositifolia:5.159411,nectandra_psammophila:5.159411,nectandra_puberula:5.159411,nectandra_reticulata:5.159411,nectandra_warmingii:5.159411)nectandra:15.478126):2.063749,(ocotea_aciphylla:11.350625,ocotea_acutifolia:11.350669,ocotea_aniboides:11.350669,ocotea_beulahiae:11.350669,ocotea_bicolor:11.350669,ocotea_brachybotra:11.350669,ocotea_campininha:11.350669,ocotea_catharinensis:11.350669,ocotea_cernua:11.350669,ocotea_complicata:11.350669,ocotea_corymbosa:11.350669,ocotea_daphnifolia:11.350669,ocotea_densiflora:11.350669,ocotea_dispersa:11.350669,ocotea_divaricata:11.350669,ocotea_elegans:11.350669,ocotea_floribunda:11.350669,ocotea_glaziovii:11.350669,ocotea_indecora:11.350669,ocotea_insignis:11.350669,ocotea_lanata:11.350669,ocotea_lancifolia:11.350669,ocotea_laxa:11.350669,ocotea_lobbii:11.350669,ocotea_longifolia:11.350669,ocotea_mandioccana:11.350669,ocotea_martiana:11.350669,ocotea_minarum:11.350669,ocotea_notata:11.350669,ocotea_nutans:11.350669,ocotea_odorifera:11.350669,ocotea_percoriacea:11.350669,ocotea_porosa:11.350669,ocotea_puberula:11.350669,ocotea_pubescens:11.350669,ocotea_pulchella:11.350669,ocotea_pulchra:11.350669,ocotea_schottii:11.350669,ocotea_silvestris:11.350669,ocotea_spectabilis:11.350669,ocotea_spixiana:11.350669,ocotea_tabacifolia:11.350669,ocotea_teleiandra:11.350669,ocotea_tenuiflora:11.350669,ocotea_urbaniana:11.350669,ocotea_vaccinioides:11.350669,ocotea_velloziana:11.350669,ocotea_velutina:11.350669,ocotea_venulosa:11.350669)ocotea:11.350625):4.127500,((aiouea_acarodomatifera:6.707188,aiouea_saligna:6.707241)aiouea:6.707188,(cinnamomum_amoenum:3.353594,cinnamomum_glaziovii:3.353620,cinnamomum_sellowianum:3.353620,cinnamomum_stenophyllum:3.353620,cinnamomum_triplinerve:3.353620)cinnamomum:10.060782):13.414376):16.711248,(persea_alba:11.610667,persea_fuliginosa:11.610707,persea_fulva:11.610707,persea_major:11.610707,persea_venosa:11.610707,persea_willdenovii:11.610707)persea:31.929333)corelauraceae:32.072380,((beilschmiedia_emarginata:16.202654,beilschmiedia_fluminensis:16.202681,beilschmiedia_rigida:16.202681,beilschmiedia_stricta:16.202681)beilschmiedia:27.004419,(cryptocarya_aschersoniana:21.603537,cryptocarya_citriformis:21.603556,cryptocarya_micrantha:21.603556,cryptocarya_moschata:21.603556,cryptocarya_saligna:21.603556)cryptocarya:21.603537):32.405308):24.054283,(hennecartia_omphalandra:15.600000,(((macropeplus_dentatus:0.450000,macropeplus_ligustrinus:0.450024)macropeplus:0.450000,macrotorus_utriculatus:0.900000)macro&macro:10.400001,(mollinedia_argyrogyna:2.2,mollinedia_blumenaviana:2.2,mollinedia_boracensis:2.2,mollinedia_calodonta:2.2,mollinedia_clavigera:2.2,mollinedia_elegans:2.2,mollinedia_engleriana:2.2,mollinedia_eugeniifolia:2.2,mollinedia_glabra:2.2,mollinedia_heteranthera:2.2,mollinedia_lamprophylla:2.2,mollinedia_lanceolata:2.2,mollinedia_longifolia:2.2,mollinedia_micrantha:2.2,mollinedia_oligantha:2.2,mollinedia_pachysandra:2.2,mollinedia_puberula:2.2,mollinedia_schottiana:2.2,mollinedia_triflora:2.2,mollinedia_uleana:2.2,mollinedia_widgrenii:2.2)mollinedia:9.100053)macro&others:4.300000)mollinedia&allies:84.066666):9.666672,(siparuna_brasiliensis:17.291668,siparuna_guianensis:17.291706,siparuna_reginae:17.291706)siparuna:92.041672):11.166667):1.500000,((cinnamodendron_dinisii:80.000058,(drimys_angustifolia:9.500000,drimys_brasiliensi:9.500031)drimys:70.500000)canellales:40.500000,(piper_aduncum:6.666667,piper_amalago:6.666708,piper_amplum:6.666708,piper_arboreum:6.666708,piper_caldense:6.666708,piper_caracolanum:6.666708,piper_cernuum:6.666708,piper_chimonanthifolium:6.666708,piper_claussenianum:6.666708,piper_corcovadensis:6.666708,piper_crassinervium:6.666708,piper_cuyabanum:6.666708,piper_dilatatum:6.666708,piper_diospyrifolium:6.666708,piper_divaricatum:6.666708,piper_gaudichaudianum:6.666708,piper_hispidum:6.666708,piper_hostmannianum:6.666708,piper_miquelianum:6.666708,piper_mollicomum:6.666708,piper_regnellii:6.666708,piper_rivinoides:6.666708,piper_sprengelianum:6.666708,piper_tuberculatum:6.666708,piper_umbellatum:6.666708)piper:113.833328):1.500000)magnoliidae:22.666656,(hedyosmum_brasiliense:29.8,hedyosmum_racemosum:29.8)hedyosmum:114.866714):22.666672):162.996658,(araucaria_angustifolia:243.000000,(podocarpus_lambertii:40.500000,podocarpus_sellowii:40.500031)podocarpus:202.500000)arauc_podoc:87.329987)seedplants:80.590027;**

**References of constructed clades inserted into the megatree**

**Gymnospermae**

Burleigh *et al*. (2012) *Journal of Botany* **2012**, 1-6.**Annonaceae**

Chatrou *et al*. (2012) *Bot. J. Linn. Soc.* **169,** 5-40.

**Apocynaceae**

Simões *et al*. (2006) *Ann. Mo. Bot. Gard.* **93**(4), 565-591.

Goyder *et al*. (2007) *Ann. Mo. Bot. Gard.* **94**(2), 423-434.

Ionta & Judd (2007) *Ann. Mo. Bot. Gard.* **94**(2), 360-375.

Livshultz *et al*. (2007) *Ann. Mo. Bot. Gard.* **94**(2), 324-359.

Rapini *et al*. (2007) *Ann. Mo. Bot. Gard.* **94**(2), 407-422.

Simões *et al*. (2007) *Ann. Mo. Bot. Gard.* **94**(2), 268-297.

Krings *et al*. (2008) *Syst. Bot.* **33**(2), 403-415.

Livshultz (2010) *Taxon* **59**(4), 1016-1030.

Simões *et al*. (2010) *Taxon* **59**(3), 772-790.

Hechem *et al*. (2011) *Taxon* **60** (3), 638–648.

Soares e Silva *et al*. (2012) *Syst. Bot*. **37**(3), 795–806.

Liede-Schumann & Meve (2013) *Ann. Mo. Bot. Gard.* **99**(1), 44-81.

**Araliaceae**

Nicolas & Plunkett (2009) *Mol. Phylogenet. Evol.* **53**, 134-151.

Fiaschi & Plunkett (2011) *Syst. Bot.* **36**(3), 806-817.

**Arecaceae**

Cuenca *et al*. (2008) *Mol. Phylogenet. Evol.* **46**, 760-775.

Montúfar & Pintaud (2008) *Rev. Peru. Biol.* **15**(supl. 1), 73-78.

Baker *et al*. (2009) *Syst. Biol.* **58**(2), 240-256.

Meerow *et al*. (2009) *PLoS ONE* **4**(10), e7353.

Roncal *et al*. (2011) *Biotropica* **43**(3), 324-334.

Baker *et al*. (2011) *Ann. Bot-London* **108,** 1417-1432.

Eiserhardt *et al*. (2011) *Taxon* **60**(2), 485-498.

Roncal *et al*. (2013) *Bot. J. Linn. Soc.* **171**(1), 120–139.

**Asteraceae**

Funk *et al*. (2009) *Systematics, evolution and biogeography of the compositae.* Chapter 44, pp 747–777.

**Bignoniaceae**

Olmstead *et al*. (2009) *Am. J. Bot.* **96**(9), 1731-1743.

Lohmann *et al*. (2013) *Bot. J. Linn. Soc.* **171**, 154–170.

**Boraginaceae**

Nazaire & Hufford (2012) *Syst. Bot.* **37**(3), 758-783.

**Burseraceae**

Becerra *et al*. (2012) *Taxon* **61**(2), 333–343.

**Calophyllaceae**

Ruhfel *et al*. (2011) *Am. J. Bot.* **98**(2), 306–325.

**Cannabaceae**

Yang *et al*. (2013) *Taxon* **62**(3), 473–485.

**Caricaceae**

Carvalho & Renner (2012) *Mol. Phylogenet. Evol.* **65**, 46–53.

**Celastraceae**

Simmons *et al*. (2008) *Mol. Phylogenet. Evol.* **48,** 745–757.

Coughenour *et al*. (2011) *Mol. Phylogenet. Evol.* **59**, 320–330.

Simmons *et al*. (2012) *Mol. Phylogenet. Evol.* **62**, 9-20.

Simmons *et al*. (2012) *Syst. Bot.* **37**(2), 456-467.

**Chloranthaceae**

Zhang *et al*. (2011) *J. Syst. Evol.* **49**(4), 315–329.

**Chrysobalanaceae**

Yakandawala *et al*. (2010) *Ann. Mo. Bot. Gard.* **97**(2), 259-281.

**Clusiaceae**

Ruhfel *et al*. (2011) *Am. J. Bot.* **98**(2), 306–325.

**Combretaceae**

Maurin *et al*. (2010) *Bot. J. Linn. Soc.* **162**, 453–476

**Cunoniaceae**

Bradford & Barnes (2001) *Syst. Bot*. **26**(2), 354-385.

Bradford (2002) *Ann. Mo. Bot. Gard*. **89**(4), 491-503.

Sweeney *et al*. (2004) *Ann. Mo. Bot. Gard*. **91**(2) 266-274.

Pillon *et al*. (2009) *Syst. Bot*. **34**(1), 141-148.

**Dilleniaceae**

Horn (2009) *Int. J. Plant Sci.* **170**(6), 794-813.

**Elaeocarpaceae**

Crayn *et al*. (2006) *Am. J. Bot.* **93**(9), 1328-1342.

**Euphorbiaceae**

Wurdack *et al*. (2005) *Am. J. Bot.* **92**(8), 1397-1420.

Sierra *et al*. (2010) *Taxon*, **59**(1), 101-116.

**Fabaceae**

Crisp *et al*. (2000) *Advances in Legume Systematics 9*, Royal Botanic Gardens, Kew, UK, pp 249-276.

Lavin *et al*. (2001) *Am. J. Bot*. **88**(3), 503–533.

Cubas *et al*. (2002) *Plant Syst. Evol.* **233**, 223-242.

Crisp & Cook (2003) *Advances in Legume Systematics 10*, Royal Botanic Gardens, Kew, UK, pp 253-268.

Pardo *et al*. (2004) *Plant Syst. Evol.* **244**, 93-119.

Wojciechowski *et al*. (2004) *Am. J. Bot.* **91**(11), 1846-1862.

Lewis *et al*. (2005) *Legumes of the World*. Royal Botanic Gardens, Kew, UK.

Lavin *et al*. (2005) *Syst. Biol.* **54**(4), 575-594.

MacMahon & Hufford (2005) *Int. J. Plant Sci.* **166**(3), 383-396.

Wang *et al*. (2006) *Bot. J. Linn. Soc.* **151**, 365-373.

Ribeiro *et al*. (2007) *Syst. Bot.* **32**(4), 762-771.

Boatwright *et al*. (2008) *Syst. Bot.* **33**(1), 133-147.

Brown *et al*. (2008) *Syst. Bot.* **33**(4), 739-751.

Bruneau *et al*. (2008) *Botany* **86**, 697-718.

Saslis-Lagoudakis *et al*. (2008) *Am. J. Bot.* **95**(10), 1270-1286.

Torke & Schaal (2008) *Am. J. Bot.* **95**(2), 215-228.

Schirire *et al*. (2009) *Am. J. Bot.* **96**(4), 816-852.

Sinou *et al*. (2009) *Botany* **87**, 947-960.

Stefanovic *et al*. (2009) *Syst. Bot.* **34**(1), 115-128.

De Queiroz *et al*. (2010) *Kew Bull.* **65**, 189–203.

Bouchenak-Khelladi *et al*. (2010) *Mol. Phylogenet. Evol.* **57**, 495-508.

Redden *et al*. (2010) *Syst. Bot.* **35**(3), 524-533.

Boatwright *et al*. (2011) *Taxon* **60**(1), 161-177.

Delgado-Salinas (2011) *Am. J. Bot*. **98**(10), 1694–1715.

Cardoso *et al*. (2012) *Am. J. Bot.* **99**(12), 1991-2013.

Da Silva *et al*. (2012) *Taxon* **61**(1), 93-108.

Degtjareva *et al*. (2012) *Mol. Phylogenet. Evol.* **62**, 693-707.

Manzanilla & Bruneau (2012) *Mol. Phylogenet. Evol.* **65**(1), 149-162.

Gagnon *et al*. (2013) *S. Afr. J. Bot*. **89**, 11-127.

**Hypericaceae**

Ruhfel *et al*. (2011) *Am. J. Bot.* **98**(2), 306–325.

**Lauraceae**

Chanderbali *et al*. (2001) *Ann. Missouri Bot. Gard.* **88**, 104-134.

Rohwer & Rudolph (2005) *Ann. Missouri Bot. Gard.* **92**, 153-178.

Nie *et al*. (2007) *Plant Syst. Evol.* **267**, 191-203.

Assis (2009) Sistemática e filosofia: filogenia do complexo *Ocotea* e revisão do grupo *Ocotea indecora* (Lauraceae), PhD Thesis, USP, Brazil.

Li *et al*. (2011) *Am. J. Bot.* **98**(9), 1-17.

Alves & Souza (2013) *Taxon* **62**(2), 281–290.

**Lecythidaceae**

Mori *et al*. (2007) *Am. J. Bot.* **94**(3), 289–301.

**Malpighiaceae**

Davis & Anderson (2010) *Am. J. Bot.* **97**(12), 2031-2048.

**Malvaceae**

Whitlock *et al*. (2001) *Syst. Bot.* **26**(2), 420-437.

Pfeil *et al*. (2002) *Syst. Bot.* **27**(2), 333–350.

Baum *et al*. (2004) *Am. J. Bot.* **91**(11), 1863–1871.

Nyffeler *et al*. (2005) *Org. Div. Evol.* **5**, 109–125.

Tate *et al*. (2005) *Amer. J. Bot.* **92**, 584–602.

Wilkie *et al*. (2006) *Syst. Bot.* **31**(1), 160-170.

Koopman & Baum (2008) *Syst. Bot.* **33**(2), 364-374.

Brunken & Muellner (2012) *Syst. Bot.* **37**(3), 699-711.

Duarte *et al*. (2011) *Syst. Bot.* **36**(3), 690-701.

Whitlock & Hale (2011) *Syst. Bot.* **36**(1), 129-136.

Whitlock *et al*. (2011) *Aust. Syst. Bot.* **24**, 215–225

Donnel *et al*. (2012) *Syst. Bot.* **37**(3), 712-722.

Skema (2012) *Taxon* **61**(3), 612-628.

**Melastomataceae**

Fritsch *et al*. (2004) *Amer. J. Bot*. **91**, 1105-1114.

Stone (2006) *Syst. Bot*. **31**(1), 107-121.

Goldenberg *et al*. (2008) *Int. J. Pl. Sci*. **169**, 963–979.

Goldenberg *et al*. (2012) *Taxon* **61** (5), 1040–1056.

Michelangeli *et al*. (2013) *Bot. J. Linn. Soc.* **171**, 38–60.

Penneys & Judd (2013) *Int. J. Pl. Sci.* **174**(5), 802-817.

**Meliaceae**

Muellner *et al*. (2008) *Taxon* **57**(1), 98-108.

Muellner *et al*. (2009) *Mol. Phylogenet. Evol.* **52**, 461–469.

**Monimiaceae**

Renner *et al*. (2010) *J. Biogeogr.* **37**, 1227-1238.

**Moraceae**

Clement & Weiblen (2009) *Syst. Bot.* **34**(3), 530-555.

**Myristicaceae**

Sauquet *et al*. (2003) *Bot. J. Linn. Soc*. **142**, 125-186.

**Myrtaceae**

Lucas *et al*. (2007) *Taxon* **56**(4), 1105-1128.

Biffin *et al*. (2010) *Ann. Bot-London* **106**, 79-93.

**Nyctaginaceae**

Douglas & Manos (2007) *Am. J. Bot.* **94**(5), 856–872.

**Ochnaceae**

Schneider *et al*. (2014) *Mol. Phylogenet. Evol*. **78**, 199-214

**Phyllanthaceae**

Hoffman *et al*. (2006) *Kew Bull*. **61**(1), 37-53.

Vorontsova & Hoffman (2008) *Kew Bull*. **63**(1), 41-59.

**Piperaceae**

Wanke et al. (2007) *Ann. Bot-London* **99**, 1231-1238.

**Primulaceae**

Yesson *et al*. (2009) *J. Biogeogr.* **36**, 1234–1252.

**Proteaceae**

Weston & Barker (2006) *Telopea* **11**, 314-344.

Mast *et al*. (2008) *Am. J. Bot.* **95**(7), 843–870.

Sauquet *et al*. (2009) *Mol. Phylogenet. Evol.* **51**, 31–43.

**Rhamnaceae**

Richardson *et al*. (2004) *Phil. Trans. R. Soc. Lond. B* **359**, 1495-1508.

Aagesen *et al*. (2005) *Plant Syst. Evol*. **250,** 197–214.

Kellermann & Udovicic (2007) *Telopea* **12**(1) 1-21.

**Rosaceae**

Potter *et al*. (2007) *Plant Syst. Evol.* **266**, 5-43.

Lundberg *et al*. (2009) *Mol. Phylogenet. Evol.* **51**, 269-280.

Dobes & Paule (2010) *Mol. Phylogenet. Evol.* **56**, 156-175.

Lo & Donoghue (2012) *Mol. Phylogenet. Evol.* **63**, 230-243.

**Rubiaceae**

Delprete & Córtes-B (2004) *Taxon* **53**, 347-356.

Lantz & Bremer (2004) *Bot. J. Linn. Soc*. **146**, 257-283.

Alejandro *et al*. (2005) *Amer. J. Bot*. **92**, 544-557.

Lantz & Bremer (2005) *Plant. Syst. Evol*. **253**, 159-183.

Kårehed *et al*. (2008) *Taxon* **56**, 1051-1076.

Khan *et al*. (2008) *Taxon* **57**, 7-23.

Razafimandimbison *et al*. (2008) *Mol. Phylogenet. Evol.* **48**, 207-223.

Bremer & Eriksson (2009) *Int. J. Plant Sci.* **170**(6), 766–793.

Razafimandimbison *et al*. (2009) *Ann. Missouri Bot. Gard*. **96**(1), 161-181.

Razafimandimbison *et al*. (2009) *Mol. Phylogenet. Evol.* **52**, 879-886.

Rydin *et al*. (2009) *Taxon* **58**, 793-810.

Tosh *et al*. (2009) *Ann. Missouri Bot. Gard*. **96**, 194-213.

Kainulainen *et al*. (2010) *Amer. J. Bot*. **97**(12), 1961-1981.

Sosa & Olmstead (2010) *Taxon* **59**(3), 755-771.

Alejandro *et al*. (2011) *Plant. Syst. Evol*. **296**, 1-20.

Razafimandimbison *et al*. (2011) *Taxon* **60**(4), 941-952.

Smedmark & Bremer (2011) *Taxon* **60**(5), 1397-1406.

Manns *et al*. (2012) *Int. J. Pl. Sci*. **173**(3), 261-286.

Verstraete *et al*. (2013) *Bot. J. Linn. Soc*. **173**, 407-441.

Barrabé *et al*. (2014) *Mol. Phylogenet. Evol*. **71**, 15-35.

**Sapindaceae**

Buerki *et al*. (2011) *Syst. Biol.* **60**(1), 32–44.

**Sapotaceae**

Smedmark *et al*. (2006) *Mol. Phylogenet. Evol.* **39**, 706–721.

Swenson *et al*. (2008) *Cladistics* **24**, 1006–1031.

**Simaroubaceae**

Clayton *et al*. (2009) *Syst. Biol.* **58**(4), 395–410.

**Solanaceae**

Weese & Bohs (2007) *Syst. Bot.* **32**(2), 445-463.

Olmstead *et al*. (2008) *Taxon* **57**(4), 1159-1181.

**Symplocaceae**

Fritsch *et al*. (2008) *Taxon* **57**(3), 823-852.

**Urticaceae**

Wu *et al*. (2013) *Mol. Phylogenet. Evol*. **69**(3), 814-827.

**Verbenaceae**

Marx *et al*. (2010) *Am. J. Bot.* **97**(10), 1647-1663.

**Violaceae**

Tukuoka (2008) *J. Plant Res*. **121**, 253-260.

**Vochysiaceae**

Sytsma *et al*. (2004) *Int. J. Plant Sci.* **165**(4 Suppl.), S85–S105.

**Winteraceae**

Marquínez *et al*. (2009) *Mol. Phylogenet. Evol.* **53**(2), 435-449.

**References of clades age estimates combined with Bell *et al*. (2010) age estimates**

Bell *et al.* (2010). *Am. J. Bot.* **97**(8), 1296-1303.

**Euphyllophytes, Monilophytes, Seed Plants & Gymnosperms**

Magallón *et al*. (2013) *Am. J. Bot.* **100**(3), 556–573.

**Apocynaceae**

Rapini *et al*. (2007) *Ann. Mo. Bot. Gard.* **94**(2), 407-422.

**Arecaceae**

Cuenca *et al*. (2008) *Mol. Phylogenet. Evol.* **46**, 760-775.

Meerow *et al*. (2009) *PLoS ONE* **4**(10), e7353.

Roncal *et al*. (2011) *Biotropica* **43**(3), 324-334.

Couvreur *et al*. (2011) *BMC Biol.* **9,** 44.

Eiserhardt *et al*. (2011) *Taxon* **60**(2), 485-498.

**Bignoniaceae**

Lohmann *et al*. (2013) *Bot. J. Linn. Soc.* **171**, 154–170.

**Caricaceae**

Carvalho & Renner (2012) *Mol. Phylogenet. Evol.* **65**, 46–53.

**Chloranthaceae**

Zhang *et al*. (2011) *J. Syst. Evol.* **49**(4), 315–329.

**Fabaceae**

Lavin *et al*. (2005) *Syst. Biol.* **54**(4), 575-594.

**Lauraceae**

Nie *et al*. (2007) *Plant Syst. Evol.* **267**, 191-203.

**Monimiaceae**

Renner *et al*. (2010) *J. Biogeogr.* **37**, 1227-1238.

**Myrtaceae**

Biffin *et al*. (2010) *Ann. Bot-London* **106**, 79-93.

**Primulaceae**

Yesson *et al*. (2009) *J. Biogeogr.* **36**, 1234–1252.

**Proteaceae**

Mast *et al*. (2008) *Am. J. Bot.* **95**(7), 843–870.

Sauquet *et al*. (2009) *Mol. Phylogenet. Evol.* **51**, 31–43

**Rhamnaceae**

Richardson *et al*. (2004) *Phil. Trans. R. Soc. Lond. B* **359**, 1495-1508.

**Rosaceae**

Lo & Donoghue (2012) *Mol. Phylogenet. Evol.* **63**, 230-243.
